# New Bis-Cyclometalated Iridium(III) Complexes with β-Substituted Porphyrin-Arylbipyridine as the Ancillary Ligand: Electrochemical and Photophysical Insights

**DOI:** 10.3390/ijms23147606

**Published:** 2022-07-09

**Authors:** Nuno M. M. Moura, Vanda Vaz Serra, Alexandre Bastos, Juliana C. Biazotto, Kelly A. D. F. Castro, Maria Amparo F. Faustino, Carlos Lodeiro, Roberto S. da Silva, Maria da Graça P. M. S. Neves

**Affiliations:** 1 LAQV-REQUIMTE, Department of Chemistry, University of Aveiro, 3810-193 Aveiro, Portugal; faustino@ua.pt (M.A.F.F.); gneves@ua.pt (M.d.G.P.M.S.N.); 2 Centro de Química Estrutural, Institute of Molecular Sciences, Instituto Superior Técnico, Universidade de Lisboa, Av. Rovisco Pais 1, 1049-001 Lisboa, Portugal; 3 CICECO, Department of Materials and Ceramic Engineering, University of Aveiro, 3810-193 Aveiro, Portugal; acbastos@ua.pt; 4 Department of Biomolecular Sciences, Faculty of Pharmaceutical Sciences of Ribeirão Preto, University of São Paulo, São Paulo 14040-903, Brazil; jmoraes@fcfrp.usp.br (J.C.B.); kedc2000@yahoo.com.br (K.A.D.F.C.); silva@usp.br (R.S.d.S.); 5 BIOSCOPE Group, LAQV-REQUIMTE, Chemistry Department, Faculty of Science and Technology, University NOVA of Lisbon, 2829-516 Caparica, Portugal; cle@fct.unl.pt; 6 ProteoMass Scientific Society, Madan Park, Rua dos Inventores, 2825-182 Caparica, Portugal

**Keywords:** coordination chemistry, porphyrin, cyclometalated iridium(III), PET, singlet oxygen

## Abstract

An efficient synthetic access to new cationic porphyrin-bipyridine iridium(III) bis-cyclometalated complexes was developed. These porphyrins bearing arylbipyridine moieties at β-pyrrolic positions coordinated with iridium(III), and the corresponding Zn(II) porphyrin complexes were spectroscopically, electrochemically, and electronically characterized. The features displayed by the new cyclometalated porphyrin-bipyridine iridium(III) complexes, namely photoinduced electron transfer process (PET), and a remarkable efficiency to generate ^1^O_2_, allowing us to envisage new challenges and opportunities for their applications in several fields, such as photo(catalysis) and photodynamic therapies.

## 1. Introduction

The access to new iridium(III) cyclometalated complexes is receiving much attention within the scientific community due to their promising applications in several fields [1,2,3,4]; their use in electron transfer arrays, photoelectrochemistry, (photo)catalysis, and electroluminescence, namely in organic light emitting diodes (OLEDs), are certainly of great significance [5,6,7,8,9,10,11,12]. More recently, this type of compounds shows promising features to be used as alternatives to the platinum-based anti-tumor drugs, like cisplatin, which have demonstrated several disadvantages such as neurotoxicity, elevated blood pressure, induce hearing, kidney damage, nauseas, among others. However, development of cancer cell resistance is the major drawback related to the used of platinum-based drugs [13,14,15,16,17,18]. 

Although the exploitation of organo-Ir(III) based complexes in medicine is still in an early stage of development, the studies already done showed their potential as biological probes, protein inhibitors, antimicrobial, and anticancer drugs [19,20,21,22,23,24]. Compared with other transition-metal complexes, cyclometalated iridium(III) complexes with d^6^ electronic structure have attractive photophysical features, namely tunable excitation and emission wavelengths, high Stokes shift, strong spin-orbit coupling of the iridium ion, high luminescence quantum yields, and relatively long phosphorescence lifetimes [1,2,21,22,23,25]. The relative inertness of Ir(III) complexes, due to the stability induced by the coordinative bonds involving the metal, is also an advantage in drug design, allowing the active molecule to reach the desired target [26,27].

The use of the inner core of the porphyrin and analogues framework to coordinate iridium(III) was considered in different studies, affording Ir(III) porphyrinoids, which have emerged as new sources of metal catalysts [28,29,30,31,32,33,34,35,36,37,38], and also as complexes with distinct photoluminescent properties [39,40,41,42,43,44,45,46,47,48]. Alternatively, the functionalization of the porphyrin periphery with adequate metal binding groups (e.g., pyridines, terpyridines) affording assemblies with an external active metal center has also attracted a high interest from the scientific community [49,50,51]. In most of these studies the focus has been on the use of metals like Ru, Re, Ir, and Pt [49,52,53,54,55,56], but no report concerning the use of iridium as the external metal was considered at β-pyrrolic positions.

**Scheme 1:**
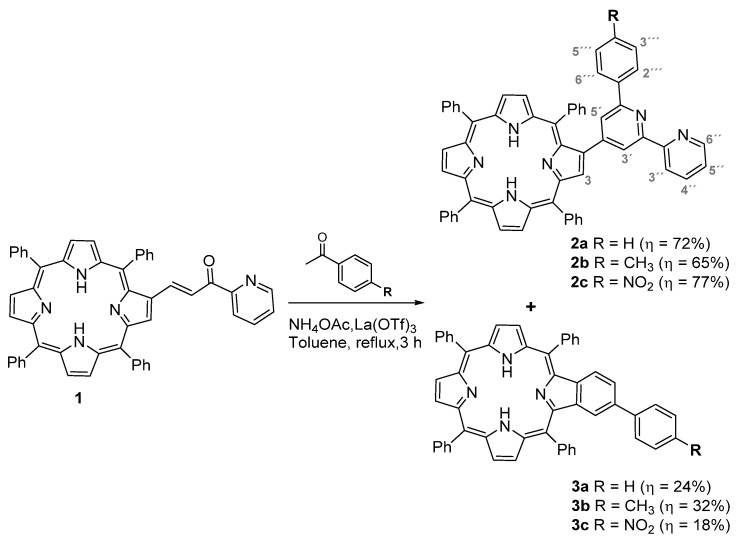
Synthetic route leading to porphyrin-bipyridine derivatives **2a–c**.

**Scheme 2:**
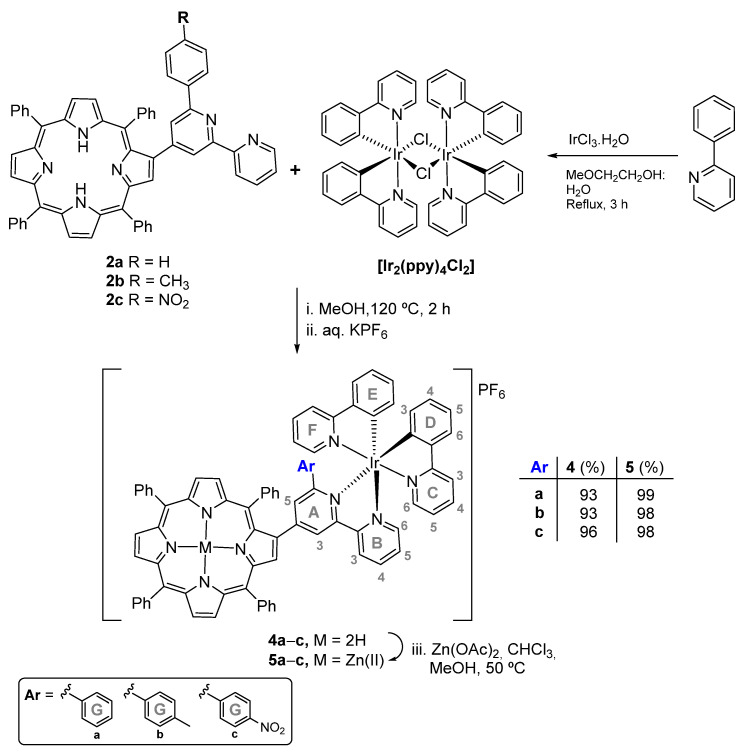
Synthetic route leading to porphyrinic iridium(III) complexes **4** and **5**.

Our interest is centered on the functionalization of β-pyrrolic positions of *meso*-tetraarylporphyrins aiming to prepare new porphyrinic derivatives with adequate properties to be used in different fields [57], namely as photosensitizers in Photodynamic Therapy (PDT) or antimicrobial PDT [13,58,59,60,61,62,63,64]. We then report here an efficient synthetic approach to obtain a novel series of mononuclear heteroleptic porphyrin-iridium(III) complexes **4** and **5** via porphyrin derivatives **2** bearing a bipyridine unit (see Scheme 1 and Scheme 2). The free base and Zn(II) complexes of porphyrin-iridium(III) derivatives were electrochemically, spectroscopically and electronically characterized, and their features were compared with the corresponding porphyrin-arylbipyridine derivatives **2**. The Zn(II) porphyrin-iridium(III) complexes showed thermodynamically favorable photoinduced electron transfer process (PET) and a remarkable ability to generate singlet oxygen (Φ_Δ_).

## 2. Results and Discussion

### 2.1. Synthesis and Structural Characterization

In a previous work we reported an efficient access to a porphyrin-chalcone type derivative **1** from reaction of 2-formyl-tetraphenylporphyrin (**TPP-CHO**) with 2-acetylpyridine (see Supplementary Information, Scheme S1) [65]. Additionally, we had verified that, in the presence of ammonium acetate and catalytic amounts of lanthanum triflate, the chalcone moieties reacted further, affording terpyridine units via Kröhnke type reaction [66].

These facts prompted us to envisage an easy synthetic approach to porphyrinic ligands of type **2** bearing a bipyridine unit (Scheme 1) that could be further used to replace the 2,20-bipyridine (bpy) in the archetypal [(ppy)_2_Ir(bpy)][PF_6_] complex.

In a typical experiment, a toluene solution of porphyrin **1**, acetophenone (5.0 equiv.), ammonium acetate (6.0 equiv.), and La(OTf)_3_ (20 mol%), was heated at reflux for 3 h. A TLC of the reaction mixture revealed the total consumption of the starting porphyrin and the formation of two new products. After the workup, the two new compounds were separated by column chromatography (silica gel). The minor and less polar compound isolated was identified by NMR and mass spectrometry as being the benzoporphyrin derivative **3a** (24%); the major compound isolated in 72% yield was identified as the desired porphyrin-bipyridine derivative **2a**. The formation of the porphyrin-phenyl pyridine derivatives follows the Kröhnke-type condensation, while the benzoporphyrinic side-products were obtained via an 1,6-Michael addition followed by intramolecular cyclization (see Scheme S2 at Supplementary Information). Briefly, a diketone intermediate is obtained by 1,6-Michael addition reaction of a carbanion to the beta-pyrrolic 3-position nearest the chalcone-type unit of derivative **1**, followed by an intramolecular aldol-type condensation and aromatization with benzaldehyde elimination.

When the described reaction conditions were extended to 4-methylacetophenone and 4-nitroacetophenone, compounds **2b** and **2c** were isolated in 65% and 77%, respectively, accompanied by the correspondent benzoporphyrins in 32% (**3b**) and 18% (**3c**).

The structures of derivatives **2** were unambiguously established by spectroscopic data, namely NMR spectroscopy (^1^H, ^13^C, and COSY) and mass spectrometry techniques (Figures S1–S14 in Supplementary Information). The asymmetry of the bipyridine moiety was confirmed by the ^1^H NMR spectra with two distinguishing duplets at ca. *δ* 7.5 and *δ* 8.5 ppm with a coupling constant of 1.2 Hz due to the resonance of the protons 3′ and 5′ from the tri-substituted pyridine. The ^1^H NMR spectra also show, in the low field region, signals corresponding to the resonance of β-pyrrolic protons and of protons 3″ and 6″ from the mono-substituted pyridine moiety; the resonances corresponding to the two remaining protons from this moiety appear for proton 4″ together with the resonances due to *ortho* protons from *meso*-phenyl groups as a multiplet at ca. *δ* 8.0–7.8 ppm and for proton 5″ as a multiplet at ca. *δ* 7.4–7.3 ppm.

The desired iridium(III) complexes **4a–c** were obtained by heating a suspension of porphyrin-bipyridine derivatives **2a–c** in methanol with [**Ir_2_(ppy)_4_Cl_2_**] (1.1 equiv.) in a sealed tube at 120 °C (Scheme 2). The monitorization of the reaction progress by TLC showed, after 2 h, the total consumption of the starting porphyrin-bipyridine derivatives **2**, with the formation of a more polar product. After the work-up, chromatographic purification, and crystallization (see details in Materials and Methods), compounds **4a–c** were isolated in excellent yields (93–96%) and their structures were confirmed by NMR spectroscopy (^1^H, ^13^C, and COSY) and mass spectrometry techniques (Figures S16–S39 in Supplementary Information).

Although the introduction of the [Ir(ppy)_2_] moiety induced a higher complexity in the NMR spectra, a characteristic pattern in the aromatic region, from ca. *δ* 9.2 to *δ* 5.4 ppm, assigned to the highly asymmetrical phenylpyridine moieties can be visualized (see Supplementary Information, Figures S16–S36). The molecular formula of **4a–c** were also unambiguously confirmed by high resolution mass spectrometry ESI(+)-MS analysis showing the peak corresponding to the respective [M]^+•^ ion; in all cases was also observed in ESI(+)-MS spectra, a peak at *m*/*z* 501 corresponding to the fragment [C_22_H_16_IrN_2_]^+•^ (see Supplementary Information).

Complexes **5a–c** were obtained in quantitative yields by metalation of the respective free base derivatives **4a–c** with zinc(II) acetate according to conventional procedures [67]. The ^1^H NMR of compounds **5a–c** showed the disappearance of the resonances of the inner *N*H at high fields but maintain the same profile observed for the proton resonances of the porphyrin-bipyridine groups and [Ir(ppy)]^+^ moieties (Supplementary Information, Figures S40–S51).

### 2.2. Electrochemistry

**Figure 1:**
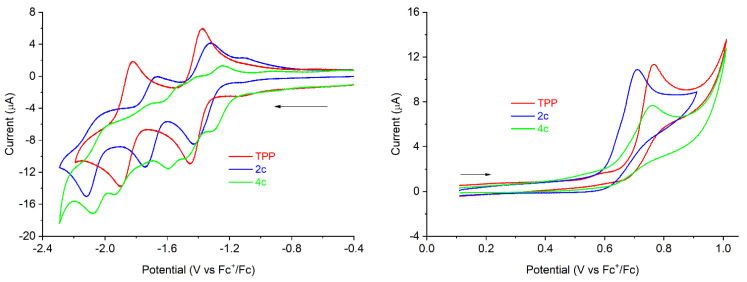
Cyclic voltammograms of **TPP**, **2c**, and **4c** (1 mM) in DMF with 0.1 M TBAPF_6_ as a supporting electrolyte obtained at a scan rate of 100 mV s^−1^.

The cyclic voltammograms of the synthesized porphyrins were performed using DMF as solvent (Figure S52). As an example, Figure 1 shows a typical cyclic voltammetry of **TPP** and of compounds **2c** and **4c**. The studied systems show multiple redox processes, and the peak potentials are dependent on the nature of the macrocycle substituents as well as of the presence of the metal ion in the macrocycle core (Table 1). It is known that the redox potentials are influenced by the π-extension of the aromatic ring [41].

Two reversible reduction processes are observed in **TPP**, at E^1/2^ = −1.41 V_Fc+/Fc_ and at E^1/2^ = −1.86 V_Fc+/Fc_, corresponding to the reduction of the porphyrinic π-system [41]. An oxidation process at 0.76 V_Fc+/Fc_ was also observed and was attributed to the oxidation of the porphyrin ring, leading to the formation of a π-radical cation [41]. In the **TPP** Zn(II) derivative (**ZnTPP**), the oxidation with the formation of a porphyrin π-radical cation occurs at lower potential, 0.51 V_Fc+/Fc_. A second one-electron oxidation process occurs on the porphyrin ring at higher potential. The coordination of Zn^2+^ on the **TPP** site shifts the reduction peaks to more negative values, respectively, −1.69 and −2.07 V_Fc+/Fc_. The change in the electrochemical potential of porphyrins with the Zn(II) metalation is consistent with the UV-visible spectrum change. The insertion of electroactive moieties on the porphyrins generates more complex cyclic voltammograms with an increased number of peaks. As an example, the cyclic voltammogram of **2c** shows an extra reduction at −2.07 V_Fc+/Fc_ that can be attributed to the process centered on the bipyridine ligand.

For the binuclear species **4** and **5**, the changes in the oxidation and reduction peaks are consistent with the electroactivity of the introduced iridium(III) moiety (Table 1 presents the potentials of the first oxidation and first reduction peaks). The introduction of the iridium(III) counterpart in the β-pyrrolic position causes noticeable changes in the redox processes, which are localized on porphyrin centers (**TPP** and **ZnTPP** nucleus for compounds **4a–c** and **5a–c**, respectively). The first oxidation potentials of porphyrin nucleus in **4a–c** are similar to that found for **TPP** (0.72 V_Fc+/Fc_ (**4a**), 0.74 V_Fc+/Fc_ (**4b**), 0.76 V_Fc+/Fc_ (**4c**), and 0.76 V_Fc+/Fc_ (**TPP**)), while their first reduction potentials are less negative (−1.26 V_Fc+/Fc_ (**4a**), −1.27 V_Fc+/Fc_ (**4b**), and −1.28 V_Fc+/Fc_ (**4c**), vs −1.41 V_Fc+/Fc_ (**TPP**)). The same trend is observed in the CV of the zinc(II) complexes (first reduction potentials of −1.34 V_Fc+/Fc_ (**5a**), −1.11 V_Fc+/Fc_ (**b**) and −1.10 V_Fc+/Fc_ (**5c**) compared to −1.69 V_Fc+/Fc_ (**ZnTPP**)). This means that the porphyrinic core is more easily reduced in the porphyrin-iridium(III) dyads. The energy gap is also narrower in the new synthesized porphyrins.

### 2.3. Spectroscopic and Photophysical Properties

**Figure 2:**
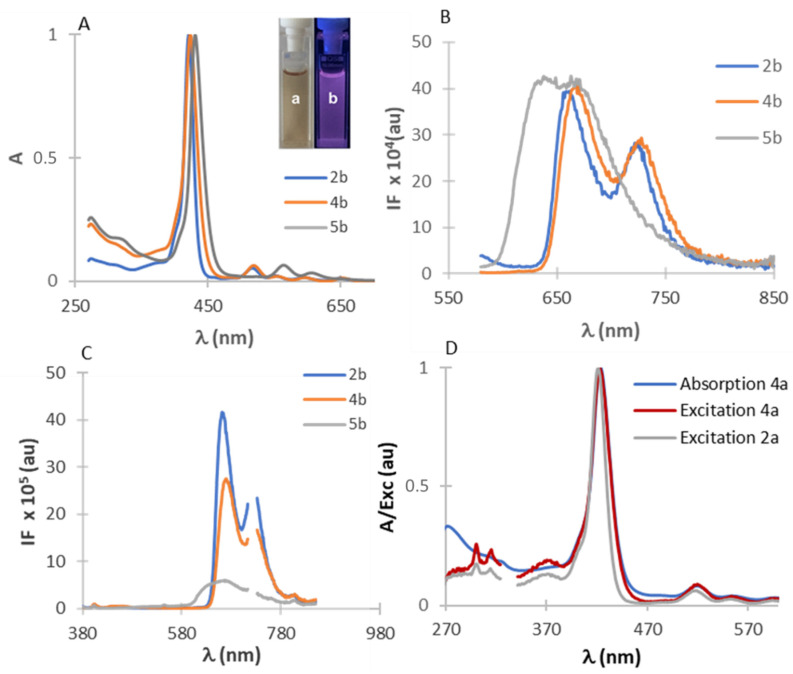
(**A**) UV-Vis absorption and (**B**,**C**) Fluorescence emission spectra of compounds **2b**, **4b**, and **5b** in DMF at room temperature (non-degassed solutions) at a concentration of approximately 6 × 10^−6^ M and λ_exc_ = 565 and 360 nm, respectively. (**D**) Absorption spectra of **4a** and Excitation spectra of diluted solutions of **2a** and **4a** in DMF at room temperature, λ_exc_ = 650 nm. Inset photographs **a** and **b**: solution of **4b** in DMF, respectively, under visible light and after excited at 365 nm. Raw data spectrum 2C are shown in Supplementary Information (Figure S55).

The absorption spectra of free base porphyrins **2a–c**, porphyrin-iridium(III) complexes **4a–c** and zinc(II) porphyrin-iridium(III) complexes **5a–c** were recorded in DMF at room temperature. A comparison of the UV-Vis spectra of porphyrin-iridium(III) complexes **4b** and **5b**, along with their parent porphyrin-bipyridine derivatives **2b**, is shown in Figure 2A (Table S1 for all compounds). These spectra were chosen as representatives of each type of compounds prepared. The absorption spectra of derivatives **2a–c** in DMF (room temperature) show the typical features of free base porphyrins—the highly intense Soret band at ca. 423 nm (due to the allowed π-π* transitions from S_0_–S_2_) and the four weak Q bands (from S_0_–S_1_ transitions). As expected, the UV-Vis spectra of porphyrin-iridium(III) complexes **4a–c** confirm porphyrin functionalization with an external [Ir(ppy)_3_] metal center as revealed by the presence of the typical bands of both allies; the intense band in the UV region 250–320 nm is assigned to the singlet–singlet ligand-centered ^1^LC band and the weaker absorption band 320–400 nm to metal-to-ligand charge transfer transitions (MLCT) in accordance with the well stablished absorption spectra of (Ir(C^N)2(N^N)) type complexes [42]. In addition, a Soret band with a maximum at 423 nm and four weaker Q bands account for the presence of porphyrin moieties in **4a–c**. The absorption profile of Zn(II)-porphyrin-iridium(III) complexes **5a–c** is very similar to that of **4a–c**. Nonetheless, as a result of symmetry changes owing to Zn(II) coordination on porphyrin inner core, red shift Soret (431 nm) and two Q bands were found as porphyrin moiety fingerprint [68]. The absorption spectra of porphyrins **4a–c** and **5a–c** are almost identical to the sum of the absorption spectra of the two separated moieties, supporting the idea that ground state inter- or intramolecular interaction between porphyrin and Ir(III) centers are absent under these experimental conditions, in line with other porphyrin-iridium(III) external complexes linked via axial coordination of the iridium complex pyridine to the porphyrinic zinc center [69].

Upon photoexcitation, derivatives **2a–c**, **4a–c**, and **5a–c** yield important NIR emission ranging from 600 to 800 nm (Figure 2B). In order to investigate the emission profiles of the new systems and to check for the possibility of iridium/porphyrin communication through photoinduced energy/electron transfer, derivatives **4a–c** and **5a–c** were studied by steady state spectroscopy upon excitation into the iridium(III) ^1^CT band (λ_exc_ = 360 nm) and also into porphyrin Q bands (λ_exc_ = 565 nm). The same measurements were performed for parent compounds **2a–c** (Figure 2B,C). The emission spectra of porphyrin-Ir(III) complexes **4a–c** obtained after excitation at ca. 565 nm present two bands centered at ca. 675 and ca. 727 nm (Figure 2B and Figure S53 in Supplementary Information). Incorporation of Zn(II) in the porphyrin core induced the expected blue shift in the emission bands of **5a–c** to 641 and 667 nm. Interestingly, upon excitation at 360 nm, where the absorption spectra of the porphyrin-iridium(III) complex is mainly due to the iridium moiety, the emission spectra of **4a–c** show a fairly strong emission at approximately 671 and 724 nm, which is characteristic of porphyrin units. A close inspection of the superposition of the fluorescence excitation spectra of compounds **4a–c** and **5a–c** with their correspondent absorption spectra (measured in diluted DMF solutions and normalized to the absorption spectra in the Q bands region where the iridium moiety does not absorb) reveals that it lacks Ir(ppy)_3_ typical bands (Figure 2D for **4a**). It might be worth noting that excitation spectra of **4a** is very similar to excitation spectra measured for porphyrin-bipyridine parent **2a** (Figure 2D), foreseeing that the absorption of light from the Ir(III) β-substituent does not contribute significantly for the observed fluorescence. Based on our experimental data, there is no clear evidence of Ir(III) to porphyrin singlet–singlet energy transfer. Nonetheless, fluorescence measured for **4a–c** and **5a–c** from excitation at 360 nm should be mainly due to the residual absorption of the porphyrin moiety and is also observed for reference **TPP** (see Figure S54 in Supplementary Information for details).

Fluorescence recorded for all compounds **2a–c**, **4a–c**, and **5a–c** (non-degassed DMF solutions) shows that porphyrin-iridium(III) and Zn(II)porphyrin-iridium(III) fluorescence is significantly quenched with respect to reference **TPP**. Fluorescence quantum yields were estimated by a comparative method with a reference compound (**TPP** in DMF, [Φ_Flu_] = 0.11) [70] and are reported in Table 2. Fluorescence from S1 is reduced to 45% for the porphyrin-iridium dyads **4a–c** and to only 27% for the corresponding zinc complexes **5a,b** (18% for **5c**) by comparison with model porphyrin **TPP** (See Table 1). Fluorescence lifetime measurements also confirm that porphyrins fluorescence is efficiently quenched by the β appended [Ir(ppy)_2_] moiety (Table 1).

The introduction of bipyridine and iridium(III) complexes as β-pyrrolic substituents increases the complexity of fluorescence lifetime decays. While a monoexponential function can be successfully used to fit experimental **TPP** and **ZnTPP** decays (11.0 ns and 2.0 ns, in accordance with literature data), monoexponential decays were never observed for **2a–c**, **4a–c**, and **5a–c** derivatives. In fact, all the decays were best fitted with a sum of two exponentials (Table 1).

For compounds **2a–c**, the two components are a longer-lived component at ~12 ns and a medium lived component at ~ 8 ns. The addition of the iridium(III) β-substituent results in a decrease of fluorescence lifetimes; fluorescence decays of compounds **4a–c** show contributions of a longer-lived component of 9.0–10.0 ns and a medium-lived component in 4.6–5.2 ns time range, with preexponential amplitudes similar to those of **2a–c**.

The interpretation of these complex decays is not straightforward. With basis on NMR data, the existence of a mixture of β-pyrrolic substituted/non-substituted porphyrins, concerning bipyridine and iridium(III) complexes counterparts, was refuted. A probable explanation for the observed multiexponential decay may arise from the possibility of these porphyrins to exist in solution as a mixture of tautomers,[71] different conformers, or may likely reflect the existence of Ir(III) structural isomers (Δ and Λ).

For compounds **5a–c**, the shorter-lived component (0.6–0.8 ns) now has a higher contribution (88–93%). A residual component with fluorescence lifetime resembling the one of **ZnTPP** was observed (2.0 ns). Time evolution of the fluorescence investigated by means of direct absorption spectroscopy (DAS) spectroscopy can be found in Supplemtary Information (Figure S56).

In DMF degassed solutions, the emission spectra of porphyrin-iridium(III) complexes **4a–c** and **5a–c** shows an additional broad and unresolved blue shifted band with a maximum between 498–506 nm, which is absent in the emission spectra of iridium free compounds **2a–c**. This band disappears in non-degassed solutions, as stated in Figure S57. Additionally, the solution emission lifetimes are in the microsecond range (*ca.* τ (**5a**) = 2.6 μs, Figure S58 and Table S2), which are substantially higher than those obtained in non-degassed solutions. Emission data are in accordance with the ones reported previously for Ir(ppy)_3_ complexes in degassed toluene and acetonitrile and are attributed to the phosphorescence of the Ir(III) peripheral subunit [72,73]. The porphyrin typical fluorescence bands observed between 600–800 nm are unchanged.

To understand the feasibility of photoinduced electron transfer process (PET), the free energy of this process (ΔG_PET_) was estimated using the following equation [74]

ΔG_PET_ = E_CT_(D-A) – E_0-0_(D) (1)

where E_CT_(D-A) is the energy of the charge transfer state of donor and acceptor obtained from the first oxidation and first reduction potentials determined by cyclic voltammetry (E_gap_ in Table 1) and E_0-0_(D) is the zero-to-zero transition of porphyrin donor determined by the intersection of the normalized absorption and emission spectra. The results are summarized in Table 3.

The calculated values predict that PET is thermodynamically favorable for Zn(II) complexes **5a–c**. Other photoinduced processes might be in the origin of porphyrin fluorescence quenching observed: (i) an increase in the porphyrin radiative rate constant; (ii) porphyrin enhanced non radiative return to the ground state; (iii) porphyrin improved intersystem crossing to the triplet state.

Recently, some Ir(III)-porphyrins and Ir(III)-corrole complexes obtained by iridium(III) coordination to the porphyrinoid central core were found to photosensitize molecular oxygen, showing singlet oxygen quantum yields (Φ_Δ_) ranging from 0.09–0.88 [41,44,75].

**Figure 3:**
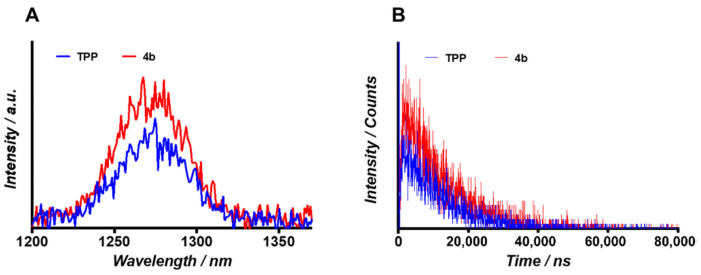
(**A**) Spectrally resolved direct detection of ^1^O_2_ generated by **TPP** and **4b** and (**B**) time-resolved direct detection of ^1^O_2_ generated by **TPP** and **4b**, λ_exc_ = 420 nm.

Singlet oxygen luminescence measurements at 1270 nm were performed for all compounds in order to determine Φ_Δ_ by a reference methodology, which uses **TPP** as standard in DMF (Φ_Δ_ = 0.65) [76,77]. The optical density of all the solutions was adjusted to 0.1 at 420 nm. As an example, the phosphorescence spectrum and a decay profile for complex **4b** is shown in Figure 3. The experimental results obtained for all compounds are reported in Table 4.

The experimental data clearly demonstrate that porphyrin-bipyridine iridium(III) **4a–c** and **5a–c** complexes are better ^1^O_2_ generators than the respective precursors **2a–c** (and also than reference **TPP**). For porphyrins **4a–c**, singlet oxygen quantum yields were found to be 9–14% higher than those determined for porphyrin free-bases **2a–c**. It is well known from the literature that the presence of a metal ion in the porphyrinic inner core of a porphyrin increases the spin orbital coupling and the intersystem, crossing to the triplet state in a classical phenomenon known as heavy atom effect [78]. Our results indicate that the presence of an external heavy iridium(III) moiety also shows a similar effect to that extent. An additional increase of 12–21% singlet oxygen quantum yield was found for **Zn(II)** porphyrins **5a–c** when compared to their precursors **4a–c**. This high ability to photogenerate ^1^O_2_ allows us to consider this series of compounds as potential candidates for light-driven therapy. Considering such an important increase in singlet oxygen quantum yield, porphyrin fluorescence quenching observed for porphyrin-bipyridine iridium(III) complexes **4a–c** and for the corresponding zinc(II) complexes **5a–c** is attributed to the presence of external and/or internal metal Ir/Zn centers, respectively.

## 3. Materials and Methods

### 3.1. General Remarks 

^1^H and ^13^C solution NMR spectra were recorded on Bruker Avance 300 (300.13 and 75.47 MHz, respectively), 500 (500.13 and 125.76 MHz, respectively), and 700 (700.13 MHz) spectrometers. CDCl_3_ was used as solvent and tetramethylsilane (TMS) as internal reference; the chemical shifts are expressed in δ (ppm) and the coupling constants (*J*) in Hertz (Hz).

Unequivocal ^1^H assignments were made using 2D COSY (^1^H/^1^H), while ^13^C assignments were made based on 2D HSQC (^1^H/^13^C) and HMBC (delay for long-range *J* C/H couplings were optimized for 7 Hz) experiments. Mass spectra were recorded using MALDI TOF/TOF 4800 Analyzer, Applied Biosystems MDS Sciex, with CHCl_3_ as solvent and without matrix. Electrospray ionization mass spectra were acquired with a Micromass Q-Tof 2 (Micromass, Manchester, UK), operating in the positive ion mode, equipped with a Z-spray source, an electrospray probe, and a syringe pump. Source and desolvation temperatures were 80 °C and 150 °C, respectively. Capillary voltage was 3000 V. The spectra were acquired at a nominal resolution of 9000 and at cone voltages of 30 V. Nebulization and collision gases were N_2_ and Ar, respectively. Porphyrin solutions in methanol were introduced at a 10 μL/min flow rate. Mass spectra HRMS-ESI(+) were recorded on a LTQ Orbitrap XL mass spectrometer (Thermo Fischer Scientific, Bremen, Germany) using CHCl_3_ as solvent. The UV-Vis spectra were recorded on an UV-2501PC Shimadzu spectrophotometer using DMF as solvent. Preparative thin-layer chromatography was carried out on 20 × 20 cm glass plates coated with silica gel (0.5 mm thick). Column chromatography was carried out using silica gel (35–70 mesh, Merck, Darmstadt, Germany). Analytical TLC was carried out on precoated sheets with silica gel (Merck 60, 0.2 mm thick).

All the chemicals were used as supplied. Solvents were purified or dried according to the literature procedures [79].

### 3.2. Synthesis

#### 3.2.1. Synthesis of the Starting Porphyrin **TPP-CHO**

The 2-formyl-5,10,15,20-tetraphenylporphyrin (**TPP-CHO**) was prepared from 5,10,15,20-tetraphenylporphyrinatocopper(II), *N,N’*-dimethylformamide (DMF) and phosphorus oxychloride (POCl_3_), according to literature procedures [76,80].

#### 3.2.2. Synthesis of the **2-[3-oxo-3-(pyridin-2-yl)prop-1-en-1-yl]-5,10,15,20-tetraphenylporphyrin, 1**

To a solution of 2-acetylpyridine (1.2 equiv.) in dry toluene (1 mL), piperidine (1.5 equiv.) was added, and the mixture was stirred for 30 min at room temperature. After this time, 2-formyl-5,10,15,20-tetraphenylporphyrin (**TPP-CHO**) and La(OTf)_3_ (20 mol%) were added and the resulting mixture was heated at reflux for 24 h. After cooling, the reaction mixture was washed with water and extracted with chloroform. The organic layer was separated, dried under Na_2_SO_4_, and the solvent was evaporated under reduced pressure. The crude mixture was purified by column chromatography (silica gel) using toluene-light petroleum (1:1) and toluene as the eluent. The compound isolated was then crystallized from CH_2_Cl_2_-hexane and fully characterized by NMR, mass and UV-Vis techniques. The structure of compound **1** is in accordance with the literature [65].

#### 3.2.3. Synthesis of the Porphyrin-Bipyridine Derivatives **2a–c**: General Procedure

To a solution of the appropriate acetophenone (5.0 equiv.) in dry toluene (1 mL), ammonium acetate was added (6.0 equiv.), and the mixture was stirred for 30 min at room temperature. After this time, porphyrin-chalcone type derivative **1** and La(OTf)_3_ (20 mol%) were added to the mixture and heated at reflux for 3 h. After cooling, the reaction mixture was washed with water and extracted with dichloromethane. The organic phase was dried (Na_2_SO_4_) and the solvent was evaporated under reduced pressure. The crude mixture was submitted to column chromatography (silica gel) using CH_2_Cl_2_ as eluent. The fractions obtained were fully characterized by NMR, mass, UV-Vis, and fluorescence techniques. The reactional conditions and yields are summarized in Scheme 1.

The characterization of compounds **3a–c** were performed by UV-Vis, ^1^H NMR, and mass spectrometry, and all the experimental data are in agreement with the described literature data [76].

**2-(6-phenyl-[2,2′-bipyridin]-4-yl)-5,10,15,20-tetraphenylporphyrin, 2a**.

**^1^H NMR** (300 MHz, CDCl_3_): *δ* 8.88–8.76 (7H, m, H-β), 8.69–8.66 (2H, m, H-6″ and H-3″), 8.45 (1H, d, *J* = 1.2 Hz, H-3′), 8.29–8.23 (6H, m, H-*o*-Ph), 8.08 (2H, d, *J* = 7.0 Hz, H-2‴ and H-6‴), 7.99–7.87 (3H, m, H-*o*-Ph, and H-4″), 7.82–7.69 (9H, m, H-*m*, *p*-Ph), 7.57 (1H, d, *J* = 1.2 Hz, H-5′), 7.53–7.41(3H, m, H-3‴, H-4‴ and H-5‴), 7.39–7.31 (1H, m, H-5″), 7.06 (3H, m, H-*m*, *p*-Ph), -2.63 (2H, s, *N*-H) ppm. **^13^C NMR** (75 MHz, CDCl_3_): *δ* 156.6, 155.1, 154.5, 149.0, 142.2, 141.8, 140.4, 139.5, 136.8, 134.6, 134.5, 132.0–130.2 (C-β), 128.7, 128.6, 127.9, 127.8, 127.0, 126.8, 126.7, 123.5, 122.5, 121.6, 121.0, 120.8, 120.4, 120.3, 120.2 ppm. **MS** (MALDI): *m*/*z* 844.3 [M]^+^^•^. **HRMS-ESI**(+): *m*/*z* calculated for C_60_H_41_N_6_ [M+H]^+^ 845.33927; found 845.33829. **UV-Vis** (DMF): λ_max_ (log ε) 420 (5.75), 515 (4.44), 550 (4.01), 590 (3.91), 645 (2.25) nm.

**2-(6-(*p*-tolyl)-[2,2′-bipyridin]-4-yl)-5,10,15,20-tetraphenylporphyrin, 2b**.

**^1^H NMR** (300 MHz, CDCl_3_): *δ* 8.88–8.81 (5H, m, H-β), 8.79 and 8.78 (2H, AB system, *J* = 5.0 Hz, H-β), 8.68–8.66 (2H, m, H-6″, and H-3″), 8.42 (1H, d, *J* = 1.2 Hz, H-3′), 8.25–8.22 (6H, m, H-*o*-Ph), 7.84 (4H, m, H-*o*-Ph, and H-2‴, 6‴), 7.93–7.85 (2H, m, H-*o*-Ph, and H-4″), 7.78–7.73 (9H, m, H-*m*, *p*-Ph), 7.53 (1H, d, *J* = 1.2 Hz, H-5′), 7.36–7.33 (1H, m, H-5″), 7.30 (2H, d, *J* = 8.1 Hz, H-3‴ and H-5‴), 7.12–7.02 (3H, m, H-*m*, *p*-Ph), 2.43 (3H, s, Ph-CH_3_), -2.63 (2H, s, *N*-H) ppm. **^13^C NMR** (75 MHz, CDCl_3_) *δ* 156.7, 155.1, 154.4, 149.0, 148.6, 142.2, 141.9, 140.4, 138.7, 136.8, 136.7, 134.6, 134.5, 132.1–130.4 (C- β), 129.3, 127.9, 127.83, 127.77, 126.9, 126.8, 126.7, 123.5, 122.2, 121.6, 121.0, 120.5, 120.4, 120.3, 120.1, 21.3 ppm. **MS** (MALDI): *m*/*z* 858.3 [M]^+^^•^. **HRMS-ESI**(+): *m*/*z* calculated for C_61_H_43_N_6_ [M+H]^+^ 859.35492; found 859.35504. **UV-Vis** (DMF): λ_max_ (log ε) 422 (5.23), 518 (3.95), 553 (3.55), 593 (3.44), 649 (3.26) nm.

**2-(6-(4-nitrophenyl)-[2,2′-bipyridin]-4-yl)-5,10,15,20-tetraphenylporphyrin, 2c**.

**^1^H NMR** (300 MHz, CDCl_3_): *δ* 8.90–8.80 (6H, m, H-β), 8.77 (1H, d, *J* = 4.9Hz, H-β), 8.71 (1H, d, *J* = 4.1 Hz, H-6″), 8.64 (1H, d, *J* = 8.0 Hz, H-3″), 8.59 (1H, d, *J* = 1.1 Hz, H-3′), 8.35 (2H, d, *J* = 8.9 Hz, H-3‴ and H-5‴), 8.26–8.23 (8H, m, H-*o*-Ph, H-2‴ and H-6‴), 8.03–7.91 (3H, m, H-*o*-Ph and H-4″), 7.79–7.74 (9H, m, H-*m*, *p*-Ph), 7.61 (1H, d, *J* = 1.1 Hz, H-5′), 7.41–7.37 (1H, m, H-5″), 7.12–7.00 (3H, m, H-*m*, *p*-Ph), -2.63 (2H, s, *N*-H) ppm. **^13^C NMR** (75 MHz, CDCl_3_) *δ* 156.0, 155.1, 152.4, 149.2, 148.0, 145.4, 142.2, 142.1, 141.7, 140.6, 137.0, 134.6, 134.5, 132.4–130.0 (C-β), 127.9, 127.8, 127.7, 126.8, 126.7, 123.9, 123.1, 122.0, 121.5, 120.7, 120.6, 120.3. **MS** (MALDI): *m*/*z* 889.2 [M]^+^^•^. **HRMS-ESI**(+): *m*/*z* calculated for C_60_H_40_N_7_O_2_ [M+H]^+^ 890.32435; found 890.32475. **UV-Vis** (DMF): λ_max_ (log ε) 423 (5.41), 519 (4.13), 554 (3.77), 594 (3.73), 650 (3.58) nm.

**[Ir(ppy)_4_Cl]_2_** was synthesized following the procedures previously described and characterized by ^1^H NMR spectroscopy. The recorded data are in accordance with the literature [81].

**^1^H NMR** (300 MHz, CDCl_3_): *δ* 9.24 (4H, d, *J* = 5.8 Hz), 7.87 (4H, d, *J* = 7.8 Hz), 7.73 (4H, td, *J* = 7.8, 1.5 Hz), 7.49 (4H, dd, *J* = 7.8, 1.2 Hz), 6.81–6.70 (9H, m), 6.56 (4H, td, *J* = 7.7, 1.5 Hz), 5.93 (4H, dd, *J* = 7.8, 1.2 Hz) ppm.

#### 3.2.4. Synthesis of Compounds **4a–c**: General Procedure

In a sealed tube, the dichloro-bridged diiridium complex **[Ir(ppy)_4_Cl]_2_** (1.1 equiv.) was added to the appropriate porphyrin-bipyridine **2a–c** in methanol (1 mL). Then, the resulting suspension was heated at 120 °C for 2 h. After cooling, an excess of an aqueous solution of KPF_6_ was added to the mixture and the resulting precipitate was filtered, washed several times with water, and then with diethyl ether. After being dried under vacuum, the crude mixture was submitted to column chromatography (silica gel) using CH_2_Cl_2_/MeOH (97:3) as eluent. Compounds **4** were obtained after crystallization from CH_2_Cl_2_/MeOH in 93% (**4a** and **4b**) and 96% (**4c**) (see Scheme 2). The compounds obtained were fully characterized by NMR, mass, UV-Vis, and fluorescence techniques.

**Bis[2-(2pyridinyl-*N*)phenyl-*C*][** **2-(6-phenyl-[2,2′-bipyridin]-4-yl)-5,10,15,20-tetraphenylporphyrin-*N*^1^,*N*^1′^]iridium(III) hexafluorophosphate, 4a.**

**^1^H NMR** (500 MHz, CDCl_3_): δ 9.22 (1H, d, *J* = 5.3 Hz, H-6^F^), 8.90 and 8.89 (2H, AB system, *J* = 5.0 Hz, H-β), 8.86 (1H, s, H-3), 8.83 (1H, d, *J* = 4.9 Hz, H-β), 8.80 (1H, d, *J* = 4.9 Hz, H-β), 8.78 and 8.76 (2H, AB system, *J* = 4.8 Hz, H-β), 8.39 (d, 1H, *J* = 8.0 Hz, H-3^B^), 8.35 (1H, s, H-3^A^), 8.32–8.28 (2H, m, H-*o*-Ph), 8.22–8.13 (6H, m, H-*o*-Ph, and H-4^B^), 7.92–7.68 (17H, m, H-*o*-Ph, H-*m*, *p*-Ph, H-3^F^, H-4^F^, H-6^B^, and H-3^G^, 5^G^), 7.47–7.43 (3H, m, H-*m*, *p*-Ph, H-3^C^ and H-3^E^), 7.35 (1H, t, *J* = 6.6 Hz, H-5^B^), 7.28 (1H, s, H-5^A^), 7.26–7.23 (1H, m, H-6^D^), 6.91–6.86 (2H, m, H-4^G^, and H-4^C^), 6.79 (1H, t, *J* = 7.4 Hz, H-5^C^), 6.74–6.69 (4H, m, H-5^F^, H-4^E^, and H-2^G^, 6^G^), 6.59–6.53 (2H, m, H-5^E^, and H-4^D^), 6.33 (1H, td, *J* = 7.5 and 0.8 Hz, H-5^D^), 5.93–5.90 (2H, m, H-6^C^ and 6^E^), 5.48 (1H, d, *J* = 7.5 Hz, H-6^D^), -2.64 (2H, s, *N*-H) ppm. **^13^C NMR** (125 MHz, CDCl_3_): δ 168.8, 168.5, 167.5, 163.8, 156.1, 155.3, 151.7, 151.4, 150.8, 150.3, 147.4, 145.3, 143.7, 142.4, 142.0, 141.8, 141.6, 139.8, 137.96, 137.6, 136.6, 136.2, 134.9, 134.8, 134.7, 134.0–132.5 (C-β), 131.3, 131.1, 130.6, 130.5, 130.3, 129.8, 129.1, 128.8, 128.0, 127.9, 127.49, 127.46, 127.0, 126.9, 126.8, 126.6, 125.3, 124.5, 124.2, 123.7, 122.9, 122.8, 122.7, 122.1, 121.3, 120.9, 120.8, 120.59, 120.55, 119.7, 119.4, 118.4 ppm. **MS** (ESI(+)): *m*/*z* 1345.5 [M]^+^^•^. **HRMS-ESI(+)**: *m*/*z* calculated for C_82_H_56_IrN_8_ 1345.42533 [M]^+^^•^; found 1345.43040. **UV-Vis** (DMF): λ_max_ (log ε) 268 (3.57), 426 (5.27), 523 (4.20), 556 (3.66), 598 (3.64), 656 (3.52) nm.

**Bis[2-(2pyridinyl-*N*)phenyl-*C*][2-(6-(*p*-tolyl)-[2,2′-bipyridin]-4-yl)-5,10,15,20-tetraphenylporphyrin-*N*^1^,*N*^1′^]iridium(III) hexafluorophosphate, 4b.**

**^1^H NMR** (500 MHz, CDCl_3_): δ 9.21 (1H, d, *J* = 6.4 Hz, H-6^F^), 8.89 and 8.88 (2H, AB system, *J* = 4.9 Hz, H-β), 8.85 (1H, s, H-3), 8.83 (1H, d, *J* = 4.9 Hz, H-β), 8.79 (1H, d, *J* = 4.9 Hz, H-β), 8.78 and 8.76 (2H, AB system, *J* = 4.8 Hz, H-β), 8.38 (1H, d, *J* = 7.9 Hz, H-3^B^), 8.33 (1H, s, H-3^A^), 8.31–8.27 (2H, m, H-*o*-Ph), 8.21–8.17 (5H, m, H-*o*-Ph), 8.12 (1H, t, *J* = 8.3 Hz, H-4^B^), 7.83–7.66 (18H, m, H-*o*-Ph, H-*m*, *p*-Ph, H3^F^, H4^F^, H-6^B^, and H-2^G^, 6^G^), 7.46–7.43 (3H, m, H-*m*, *p*-Ph, H-3^C^, and H-3^E^), 7.33 (1H, t, *J* = 6.6 Hz, H-5^B^), 7.30 (1H, s, H-5^A^), 7.25–7.23 (1H, m, H-6^D^), 6.89 (1H, td, *J* = 7.5 and 0.8 Hz, H-4^C^), 6.79 (1H, t, *J* = 7.5 Hz, H-5^C^), 6.73–6.70 (2H, m, H-5^F^ and 4^E^), 6.64 (1H, t, *J* = 7.5 Hz, H-4^D^), 6.54 (1H, td, *J* = 7.4, H-5^E^), 6.49–6.43 (2H, m, H-3^G^, 5^G^), 6.32 (1H, td, *J* = 7.4 and 0.8 Hz, H-5^D^), 5.92–5.90 (2H, m, H-6^C^ and H-6^E^), 5.45 (1H, d, *J* = 7.5 Hz, H-6^D^), 2.10 (3H, s, Ph-C*H*_3_), -2.64 (2H, s, N-*H*) ppm. **^13^C NMR** (125 MHz, CDCl_3_) δ 168.8, 168.5, 167.5, 163.9, 156.2, 155.1, 151.7, 150.8, 150.3, 147.4, 145.3, 143.7, 142.5, 142.4, 142.0, 141.8, 141.6, 139.8, 138.2, 137.96, 136.6, 136.2, 135.2, 134.9, 134.8, 134.7, 133.8–132.5 (C-β), 131.5, 131.1, 130.6, 130. 5, 130.3, 129.6, 129.1, 128.3, 128.0, 127.94, 127.89, 127.7, 127.4, 127.1, 127.0, 126.9, 126.8, 126.6, 125.2, 124.5, 124.4, 124.1, 123.7, 122.9, 122.7, 122.2, 121.3, 120.9, 120.8, 120.6, 120.2, 119.78, 119.7, 119.4, 118.4, 21.3 (Ph-CH_3_) ppm. **MS** (ESI(+)): *m*/*z* 1359.4 [M]^+^^•^. **HRMS-ESI(+)**: *m*/*z* calculated for C_83_H_58_IrN_8_ 1359.44118 [M]^+^^•^; found 1359.44390. **UV-Vis** (DMF): λ_max_ (log ε) 269 (3.81), 425 (5.27), 522 (4.17), 557 (3.36), 597 (3.24), 655 (3.12) nm.

**Bis[2-(2pyridinyl-*N*)phenyl-*C*][2-(6-(4-nitrophenyl)-[2,2′-bipyridin]-4-yl)-5,10,15,20-tetraphenylporphyrin-*N*^1^,*N*^1′^]iridium(III) hexafluorophosphate, 4c.**

**^1^H NMR** (500 MHz, CDCl_3_): 9.20 (1H, d, *J* = 5.6 Hz, H-6^F^), 8.93 (1H, s, H-3), 8.90 and 8.89 (2H, AB system, *J* = 4.9 Hz, H-β), 8.81 (1H, d, *J* = 4.9 Hz, H-β), 8.78–8.75 (3H, m, H-β), 8.55 (1H, s, H-3^A^), 8.51 (1H, d, *J* = 8.2 Hz, H-3^B^), 8.31–8.29 (2H, m, H-*o*-Ph), 8.21–8.12 (5H, m, H-*o*-Ph and H-4^B^), 8.00–7.71 (18H, m, H-*o*-Ph, H-*m*, *p*-Ph, H-6^B^, H3^F^,H-2^G^, 6^G^ and H-3^G^, 5^G^), 7.68–7.65 (1H, m, H4^F^), 7.60–7.53 (1H, m, H3^C^), 7.46–7.43 (3H, m, H-*m*, *p*-Ph, and H-3^E^), 7.34 (1H, t, *J* = 7.6 Hz, H-5^B^), 7.24–7.23 (2H, m, H-4^C^ and H-3^D^), 7.20 (1H, s, H-5^A^), 6.90 (1H, t, *J* = 7.6 Hz, H-4^E^), 6.78 (1H, t, *J* = 7.6 Hz, H-5^E^), 6.73–6.69 (1H, m, H5^F^), 6.58 (1H, t, *J* = 7.3 Hz, H-4^D^), 6.54 (1H, t, *J* = 7.4 Hz, H-5^C^), 6.30 (1H, t, *J* = 7.3 Hz, H-5^D^), 5.91 (1H, d, *J* = 7.4 Hz, H-6^C^), 5.86 (1H, d, *J* = 7.6 Hz, H-6^E^), 5.45 (1H, d, *J* = 7.3 Hz, H-6^D^), -2.63 (2H, s, *N*-H) ppm. **^13^C NMR** (125 MHz, CDCl_3_) δ 168.2, 167.3, 160.9, 155.9, 155.6, 151.6, 151.0, 150.3, 149.8, 149.1, 149.0, 147.6, 146.7, 142.6, 142.3, 141.9, 141.8, 141.5, 139.9, 138.34, 138.26, 136.9, 136.2, 134.9, 134.7, 134.6, 133.92–132.57 (C-β), 131.7, 131.2, 130.1, 129.8, 129.7, 128.7, 128.14, 128.06, 127.96, 127.6, 126.99, 126.91, 126.85, 126.5, 125.7, 125.1, 124.6, 124.2, 123.7 123.3, 123.2, 122.98, 122.85, 122.1, 121.3, 121.1, 120.9, 120.7, 120.6, 119.9, 119.5, 119.3, 118.4 ppm. **MS** (ESI(+)): *m*/*z* 1390.5 [M]^+^^•^. **HRMS-ESI(+)**: *m*/*z* calculated for C_82_H_55_IrN_9_O_2_ 1390.41176 [M]^+^^•^; found 1390.41662. **UV-Vis** (DMF): λ_max_ (log ε) 269 (3.95), 424 (5.25), 523 (4.36), 558 (4.11), 597 (4.09), 657 (4.07) nm.

#### 3.2.5. Metalation of the Free Base Derivatives **4a–c**: General Procedure

A solution of the appropriate porphyrinic derivative **4a–c** (25.0 mg) in CHCl_3_/MeOH (3:1) was stirred in the presence of Zn(OAc)_2_·H_2_O, (1.5 equiv.) for 10 min at 50 °C. The reaction was followed by TLC and UV-Vis until total consumption of the starting porphyrin. After cooling, the reaction mixture was washed with water and extracted with dichloromethane. The organic phase was dried (Na_2_SO_4_), and the solvent was evaporated under reduced pressure. The resulting residues were crystallized from CH_2_Cl_2_/hexane, and the desired compounds **5a–c** were obtained in almost quantitative yields (see Scheme 2).

**{Bis[2-(2pyridinyl-*N*)phenyl-*C*][2-6-(phenyl)-[2,2′-bipyridin]-4-yl)-5,10,15,20-tetraphenylporphyrinato-*N*^1^,*N*^1′^]zinc(II)}iridium(III) hexafluorophosphate, 5a.**

**^1^H NMR** (300 MHz, CDCl_3_): 9.16 (1H, d, *J* = 5.2 Hz, H-6^F^), 8.90 (1H, s, H-3), 8.85–8.83 (2H, m, H- β), 8.80 (1H, d, *J* = 4.8 Hz, H-β), 8.77–8.73 (2H, m, H- β), 8.68 (1H, d, *J* = 4.8 Hz, H-β), 8.44 (d, 1H, *J* = 8.2 Hz, H-3^B^), 8.35 (1H, s, H-3^A^), 8.22–8.03 (8H, m, H-*o*-Ph, and H-4^B^), 7.90–7.64 (17H, m, H-*o*-Ph, H-*m*, *p*-Ph, H-3^F^, H-4^F^, H-6^B^, and H-3^G^, 5^G^), 7.44–7.40 (3H, m, H-*m*, *p*-Ph, H-3^C^, and H-3^E^), 7.35 (1H, s, H-5^A^), 7.30–7.22 (2H, m, H-5^B^, and H-6^D^), 6.88–6.81 (2H, m, H-4^G^, and H-4^C^), 6.76–6.64 (4H, m, H-5^C^, H-5^F^, H-4^E^, and H-2^G^, 6^G^), 6.59–6.53 (2H, m, H-5^E^, and H-4^D^), 6.29 (1H, td, *J* = 7.6 and 1.2 Hz, H-5^D^), 6.00 (1H, d, *J* = 7.5 Hz, H-6^C^), 5.85 (1H, d, *J* = 7.1 Hz, 6^E^), 5.48 (1H, d, *J* = 7.4 Hz, H-6^D^) ppm. **MS** (ESI(+)): *m*/*z* 1407.3 [M]^+^^•^. **HRMS-ESI(+)**: *m*/*z* calculated for C_82_H_54_IrN_8_Zn 1407.34017 [M]^+^^•^; found 1407.34302. **UV-Vis** (DMF): λ_max_ (log ε) 268 (3.89), 431 (5.19), 565 (3.79), 608 (3.39) nm.

**{Bis[2-(2pyridinyl-*N*)phenyl-*C*][2-6-(*p*-tolyl)-[2,2′-bipyridin]-4-yl)-5,10,15,20-tetraphenylporphyrinato-*N*^1^,*N*^1′^]zinc(II)}iridium(III) hexafluorophosphate, 5b.**

**^1^H NMR** (300 MHz, CDCl_3_): 8.89 (1H, s, H-3), 8.84–8.83 (3H, m, H-β), 8.79 (1H, d, *J* = 4.8 Hz, H-β), 8.75 and 8.68 (2H, AB system, *J* = 4.8 Hz, H-β), 8.44 (1H, d, *J* = 8.2 Hz, H-3^B^), 8.34 (1H, s, H-3^A^), 8.21–8.03 (8H, m, H-*o*-Ph and H-4^B^), 7.81–7.65 (17H, m, H-*o*-Ph, H-*m*, *p*-Ph, H-3^F^, H-4^F^, H-6^B^, and H-2^G^, 6^G^), 7.48–7.42 (2H, m, H-3^C^, and H-3^E^), 7.38 (1H, s, H-5^A^), 7.29–7.22 (3H, m, H-*m*, *p*-Ph, H-5^B^, and H-6^D^), 6.87–6.83 (2H, m, H-4^C^ and H-5^C^), 6.76–6.67 (2H, m, H-5^F^, and 4^E^), 6.67–6.53 (2H, m, H-4^D^, and H-5^E^), 6.44–6.42 (2H, m, H-3^G^, 5^G^), 6.29 (1H, td, *J* = 7.7 and 1.2 Hz, H-5^D^), 6.00 (1H, d, *J* = 8.0 Hz, H-6^C^), 5.86 (2H, d, J = 7.7 Hz, and H-6^E^), 5.43 (1H, d, *J* = 7.3 Hz, H-6^D^), 2.06 (3H, s, Ph-C*H*_3_) ppm. **MS** (ESI(+)): *m*/*z* 1421.5 [M]^+^^•^. **HRMS-ESI(+)**: *m*/*z* calculated for C_83_H_56_IrN_8_Zn 1421.35441 [M]^+^^•^; found 1421.35801. **UV-Vis** (DMF): λ_max_ (log ε) 269 (3.99), 431 (5.27), 565 (4.10), 606 (3.70) nm.

**{Bis[2-(2pyridinyl-*N*)phenyl-*C*][2-6-(4-nitrophenyl)-[2,2′-bipyridin]-4-yl)-5,10,15,20-tetraphenylporphyrinato-*N*^1^,*N*^1′^]zinc(II)}iridium(III) hexafluorophosphate, 5c.**

**^1^H NMR** (300 MHz, CDCl_3_): 8.93 (1H, s, H-3), 8.89–8.83 (3H, m, H-β), 8.79 (1H, d, *J* = 4.8 Hz, H-β), 8.75 and 8.68 (2H, AB system, *J* = 4.8 Hz, H-β), 8.62–8.61 (H-3^A^ and H-3^B^), 8.30–8.11 (9H, m, H-*o*-Ph, and H-4^B^), 8.02–7.69 (17H, m, H-*m*, *p*-Ph, H-6^B^, H3^F^, H-2^G^, 6^G^, and H-3^G^, 5^G^), 7.60–7.48 (4H, m, H4^F^, H3^C^, H-*m*, *p*-Ph, and H-3^E^), 7.35–7.29 (5H, m, H-5^B^, H-4^C^, H-3^D^, H-5^A^, and H-4^E^), 7.01–9.91 (2H, m, H-5^E^ and H5^F^), 6.86–6.78 (2H, m, H-4^D^ and H-5^C^), 6.62 (1H, td, *J* = 7.4 and 0.8 Hz, H-5^D^), 6.33 (1H, td, *J* = 7.4 and 0.8 Hz, H-6^C^), 5.88 (1H, d, *J* = 7.7 Hz, H-6^E^), 5.50 (1H, d, *J* = 7.5 Hz, H-6^D^) ppm. **MS** (ESI(+)): *m*/*z* 1452.5 [M]^+^^•^. **HRMS-ESI(+)**: *m*/*z* calculated for C_82_H_53_IrN_9_O_2_Zn 1452.32390 [M]^+^^•^; found 1452.32703. **UV-Vis** (DMF): λ_max_ (log ε) 271 (3.88), 431 (5.19), 565 (3.79), 608 (3.39) nm.

### 3.3. Cyclic Voltammetry Measurements

Cyclic voltammetry was performed using an AUTOLAB PGSTAT 30 potentiostat with a three-electrode cell consisting of a Pt counter electrode, an Ag/AgCl reference electrode, and a glassy carbon working electrode (3 mm diameter). Solutions with 1.0 mM porphyrins were prepared in DMF with 0.1 M Bu_4_NPF_6_ (TBAP) as supporting electrolyte. The voltammograms were obtained with a 0.1 V s^−1^ scan rate. Ferrocene in DMF (+0.39 V versus Ag/AgCl) was employed as internal standard.

### 3.4. Spectrophotometric and Spectrofluorimetric Measurements

Absorption spectra were recorded on a PerkinElmer Lambda spectrophotometer. Corrected fluorescence measurements were recorded in a SPEX Fluorolog spectrophotometer (Horiba Jobin Yvon). Excitation at 445 nm was achieved using a NanoLED (fwhm < 1.0 ns) at a repetition rate of 1 MHz. The spectrophotometric characterizations were performed by preparing a stock solution of the compound in DMF (ca. 10^−4^ M) in a 5 mL volumetric flask. The studied solutions were prepared by appropriate dilution of the stock solution to 6 × 10^−6^ M. Luminescence quantum yields of the studied compounds **2a–c**, **4a–c**, and **5a–c** were measured in 1 × 1 cm quartz optical cells on a F4500–Hitachi spectrofluorimeter using a solution of 5, 10, 15, 20-tetraphenylporphyrin (**TPP**) in DMF as standard ([Φ_Flu_] = 0.11) [70,82,83]. All the measurements were performed at 298 K.

### 3.5. Singlet Oxygen Generation

Singlet oxygen measurements were performed in a specially designed Edinburgh F900 instrument (Edinburgh, UK) consisting of a Rainbow OPO (Quantel Laser-France) 10 Hz, 2 mJ/pulse, which was pumped by a Brilliant NdYAG laser (Quantel Laser-France) and equipped with a cuvette holder, a silicon filter, monochoromator, a liquid nitrogen-cooled NIR PMT (R5509) (Hamamatsu Co., Bridgewater, NJ, USA), and a fast multiscaler analyzer card with 5 ns/channel (MSA-300; Becker and Hickl, Berlin, Germany). All samples have the same OD (about 0.1) at the excitation wavelength, as revealed by an UV-Vis Spectrometer (Shimadzu 2400, Kioto, Japan). A volume of 3 mL of each solution was put into quartz cells (1 cm × 1 cm) and stored in the dark. The samples were irradiated at 420 nm inside a fluorescence quartz cuvette. Photosensitized steady-state singlet oxygen luminescence was measured at 1270 nm. The intensities of emission peak at 1270 nm are correlated with the amount of ^1^O_2_ generated. Kinetic curves of the ^1^O_2_ luminescence were measured using the method of time-correlated single photon counting. To calculate the singlet oxygen quantum yield (φ_Δ_), **TPP** in DMF was used as reference (φ_Δ_ = 0.65) [76].

## 4. Conclusions

In summary, this study allowed us to develop a straightforward synthetic pathway to prepare, for the first time, a new series of porphyrins bearing arylbipyridine units coordinated with iridium(III) at a β-pyrrolic position. The free base porphyrin-iridium(III) derivatives, as well as the corresponding Zn(II) complexes, were successfully synthesized with excellent yields (≥93%).

The photophysical/photochemical characterization showed that the introduction of the cyclometaled [Ir(ppy)_2_] units at the porphyrin-arylbipyridine led to conjugates with superior and outstanding ability to generate singlet oxygen compared to **TPP**. The overall structural, electrochemical, and photophysical properties of **4** and **5** are encouraging and further studies will be performed to investigate these complexes in photoinduced and photodynamic processes, namely as photocatalysts and as photosensitizers agents.

## Figures and Tables

**Table 1:** Summary of the electrochemical data of porphyrinic derivatives **TPP**, **ZnTPP**, **2a–c**, **4a–c**, and **5a–c** on a glassy carbon electrode in DMF, 0.1 M TBAPF_6_, v = 100 mV.s^−1^.

Compound	E^1/2^_1st ox_(V_Fc+/Fc_)	E^1/2^_1st red_ (V_Fc+/Fc_)	E_gap_^(1)^ (eV)
**TPP**	0.76	−1.41	2.17
**ZnTPP**	0.51	−1.69	2.20
**2a**	0.71	−1.34	2.05
**2b**	0.74	−1.34	2.08
**2c**	0.71	−1.38	2.09
**4a**	0.72	−1.26	1.98
**4b**	0.74	−1.27	2.01
**4c**	0.76	−1.28	2.04
**5a**	0.49	−1.34	1.83
**5b**	0.58	−1.11	1.69
**5c**	0.82	−1.1	1.92

^(1)^ E_gap_ = E_LUMO_ – E_HOMO_ = *e*(E^1/2^_first reduction_ – E^1/2^ _first oxidation_).

**Table 2:** Fluorescence lifetimes (τ_i_), respective pre-exponential factors (A_i_), and fluorescence quantum yields (Φ_F_) measured for porphyrins **2**, **4**, and **5 a–c** in DMF (air equilibrated samples).

Compound	τ_1_ ns (A_1_%)	τ_2_ ns(A_2_%)	χ^2^	Φ_F_^(c)^
**TPP ^(a)^**	11.0 (100)	---	1.02	0.11
**2a ^(a)^**	11.9 (24.1)	7.8 (75.9)	0.93	0.08
**2b ^(a)^**	12.0 (25.6)	7.6 (74.4)	1.03	0.09
**2c ^(a)^**	12.0 (24.4)	7.6 (75.6)	1.00	0.08
**4a ^(b)^**	10.0 (16.8)	5.2 (83.2)	1.01	0.05
**4b ^(b)^**	9.7 (23.3)	5.2 (76.7)	1.05	0.05
**4c ^(b)^**	9.0 (29.3)	4.6 (70.7)	1.07	0.05
**ZnTPP ^(a)^**	2.0 (100)	---	1.02	0.03
**5a ^(a)^**	2.0 (7.9)	0.8 (92.1)	1.00	0.03
**5b ^(a)^**	2.0 (12.5)	0.8 (87.5)	1.00	0.03
**5c ^(a)^**	2.0 (6.6)	0.6 (93.4)	0.93	0.02

λ_exc_ = 594 nm ^(**a**)^ λ_em_ = 660 nm ^(**b**)^ λ_em_ = 670 nm ^(**c**)^ estimated by comparison with **TPP** ([Φ_F_] = 0.11) [70].

**Table 3:** Zero-to-zero transition and free energy data for **TPP**, **ZnTTP**, **2a–c**, **4a–c**, and **5a–c**.

Compound	E_0-0_^(1)^ (eV)	ΔG_PET_^(2)^ (eV)
**TPP**	1.92	0.25
**ZnTPP**	2.09	0.11
**2a**	1.90	0.15
**2b**	1.89	0.19
**2c**	1.89	0.20
**4a**	1.88	0.10
**4b**	1.95	0.06
**4c**	1.95	0.09
**5a**	1.99	-0.16
**5b**	1.99	-0.30
**5c**	1.99	-0.07

^(1)^ obtained from the optical gap, ^(2)^ determined by Equation (1).

**Table 4:** Singlet oxygen quantum yield (Φ_Δ_) of porphyrins in DMF.

Compound ^(a)^	Φ_Δ_^(b)^
**2a**	0.65
**2b**	0.68
**2c**	0.66
**4a**	0.71
**4b**	0.78
**4c**	0.72
**5a**	0.79
**5b**	0.92
**5c**	0.80

^(a)^ Optical density of all samples was 0.1 at 420 nm; ^(b)^ using **TPP** as reference in DMF (Φ_Δ_ = 0.65).

## Data Availability

Not applicable.

## Supplementary Materials

The following supporting information can be downloaded at: https://www.mdpi.com/article/10.3390/ijms23147606/s1.
