# Supplementary material for: New Bis-Cyclometalated Iridium(III) Complexes with β-Substituted Porphyrin-Arylbipyridine as the Ancillary Ligand: Electrochemical and Photophysical Insights
Source: Int J Mol Sci. 2022 Jul 9;23(14):7606. doi: 10.3390/ijms23147606 (PMC9319630; doi:10.3390/ijms23147606)
Supplement: Supplementary file 1 [file ijms-23-07606-s001.zip › ijms-1768757-SI.pdf]

Electronic Supplementary Information (SI)

New bis-cyclometalated iridium(III) complexes with β-substituted porphyrin-arylbipyridine as the ancillary ligand: electrochemical and photophysical insights

Nuno M. M. Moura [1,*], Vanda Vaz Serra [2,*], Alexandre Bastos [3], Juliana C. Biazotto [4], Kelly A. D. F. Castro [4], Maria Amparo F. Faustino [1], Carlos Lodeiro [5,6], Robeerto S. da Silva [4] and Maria Graça P. M. S. Neves [1]

[1] *LAQV-REQUIMTE, Department of Chemistry, University of Aveiro, 3810-193 Aveiro, Portugal.*

[2] *Centro de Química Estrutural, Institute of Molecular Sciences, Instituto Superior Técnico, Universidade de Lisboa, Av. Rovisco Pais, 1049-001 Lisboa, Portugal.*

[3] *CICECO, University of Aveiro, 3810-193 Aveiro, Portugal.*

[4] *Department of Biomolecular Sciences, Faculty of Pharmaceutical Sciences of Ribeirão Preto, University of São Paulo, SP, Brazil.*

[5] *BIOSCOPE Group, LAQV-REQUIMTE, Chemistry Department, Faculty of Science and Technology, University NOVA of Lisbon, 2829-516 Caparica, Portugal.*

[6] *ProteoMass Scientific Society, Madan Park, Rua dos Inventores, 2825-182 Caparica, Portugal.*

Table of contents

I **-** *Synthetic route of porphyrin-chalcone type derivative* **1**……………..... S3

II **-** *Mechanistic pathway to compounds* **3a-c**.. S3

III **-** *Photophysical data for compounds* ***2a-c***, ***4a-c*** *and* ***5a-c***.................... S4

III **-** *NMR and mass spectra*... S4

IV - *Cyclic voltammograms*…………..……………………………………… S30

V - *Absorption and emission spectra* ……………………………………… S31

I - Synthetic route of porphyrin-chalcone type derivative **1**

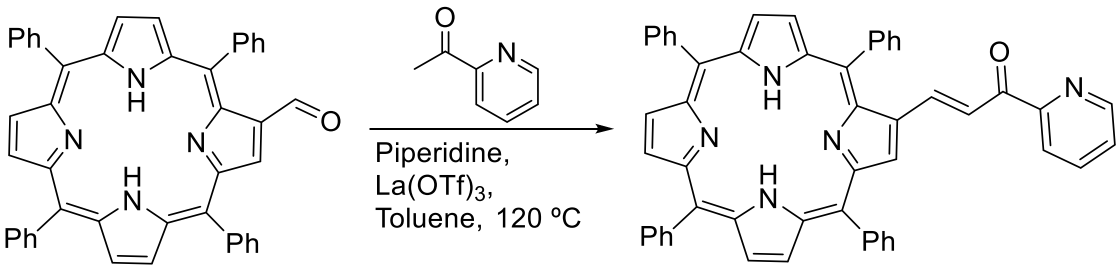

Scheme S1. Synthetic route of porphyrin-chalcone type derivative **1**.

II - Mechanistic pathway to compounds 3a-c

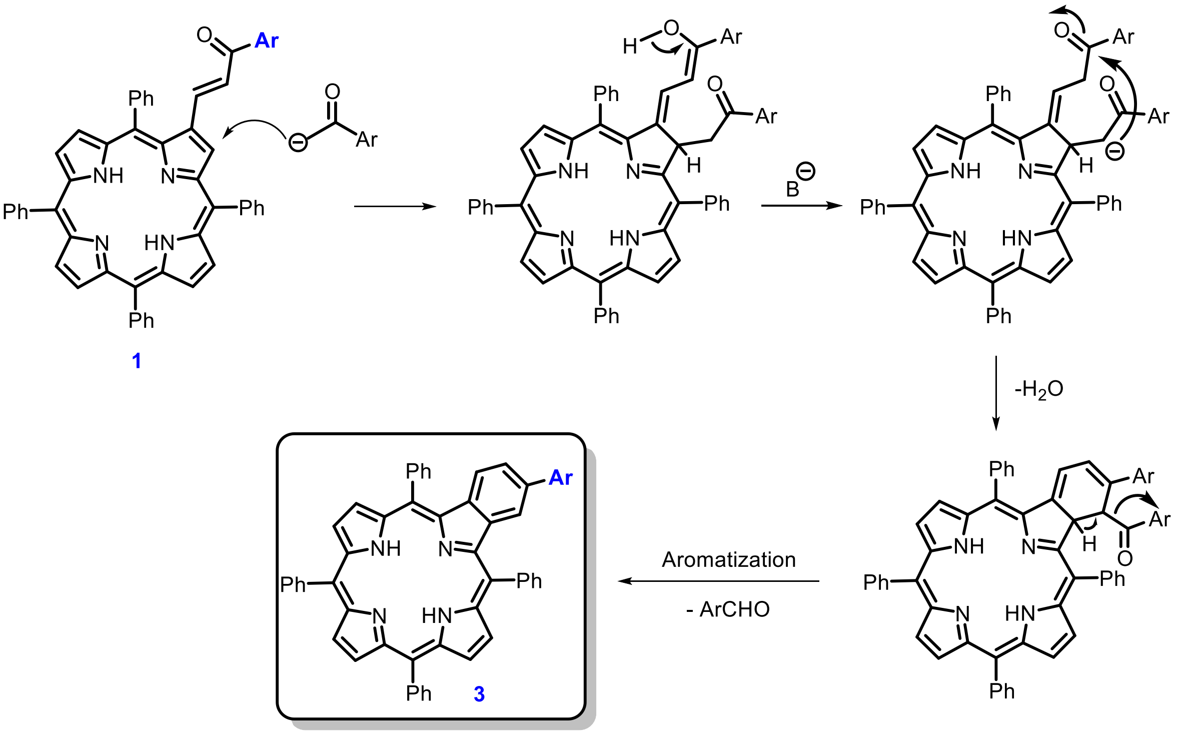

Scheme S2. Proposed mechanistic pathway to obtain benzoporphyrin derivatives **3a-c**.

III - Photophysical data for compounds **2a-c**, **4a-c** and **5a-c**

Table S1. Absorption and emission data of compounds **2a-c**, **4a-c** and **5a-c** in DMF at 298 K.

Compound	λ_{max}(nm) : log ε	λ_{em} (nm)
**2a**	420 (5.75), 515 (4.44), 550 (4.01), 590 (3.91) 645 (2.25)	661, 719
**2b**	422 (5.23), 518 (3.95), 553 (3.55), 593 (3.44), 649 (3.26)	661, 717
**2c**	423 (5.41), 519 (4.13), 554 (3.77), 594 (3.73), 650 (3.58)	661, 719
**4a**	268 (3.57), 426 (5.27), 523 (4.20), 556 (3.66), 598 (3.64), 656 (3.52)	674, 727
**4b**	269 (3.81), 425 (5.27), 522 (4.17), 557 (3.36), 597 (3.24), 655 (3.12)	673, 727
**4c**	269 (3.95), 424 (5.25), 523 (4.36), 558 (4.11), 597 (4.09), 657 (4.07)	676, 728
**5a**	268 (3.89), 431 (5.27), 565 (3.66), 606 (3.52)	641, 667
**5b**	269 (3.99), 431 (5.27), 565 (4.10), 606 (3.70)	641, 667
**5c**	271 (3.88), 431 (5.19), 565 (3.79), 608 (3.39)	641, 667

III - NMR and mass spectra

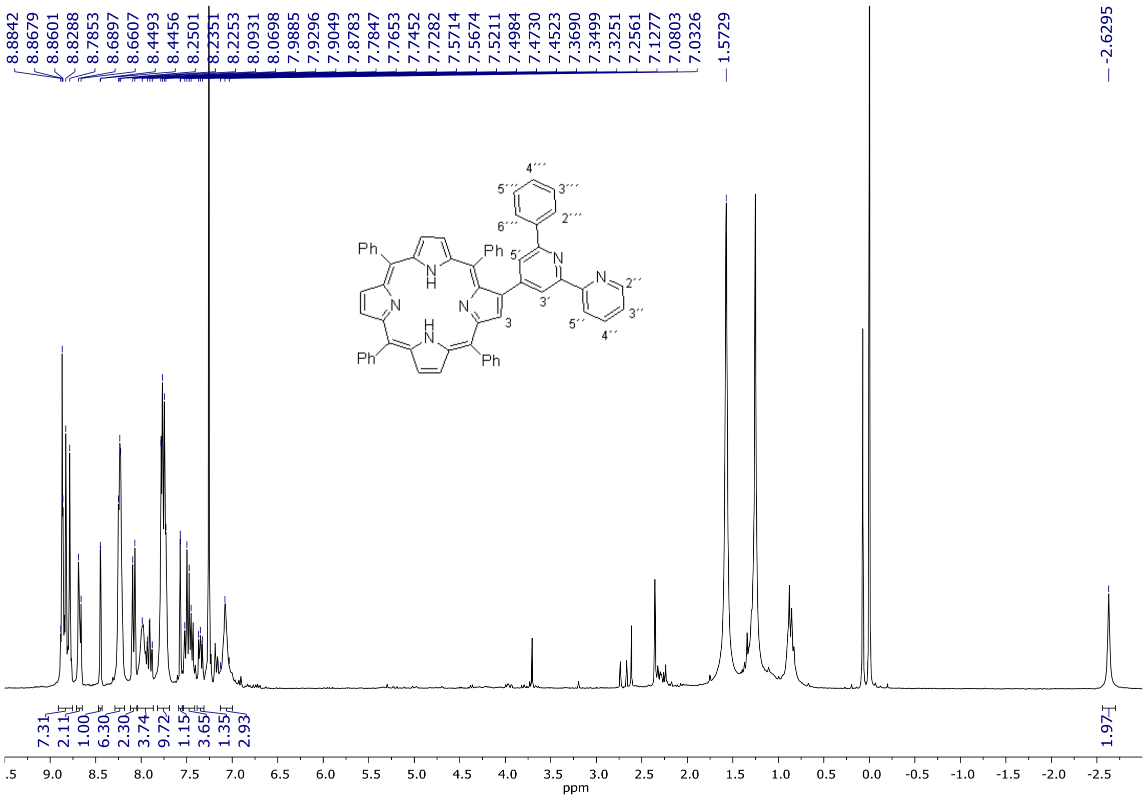

Figure S1. ^{1}H NMR spectrum of compound **2a** in $CDCl_3$.

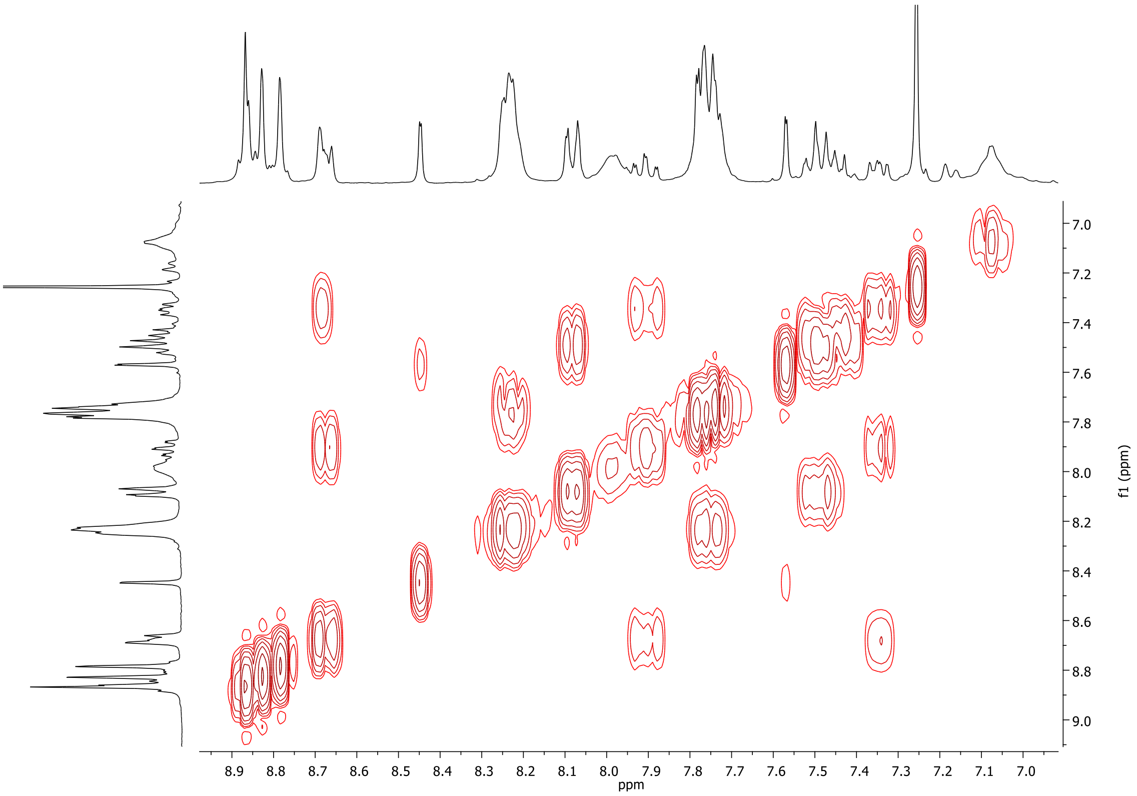

Figure S2. Partial COSY ($^{1}H/^{1}H$) spectrum of Compound **2a** in $CDCl_3$.

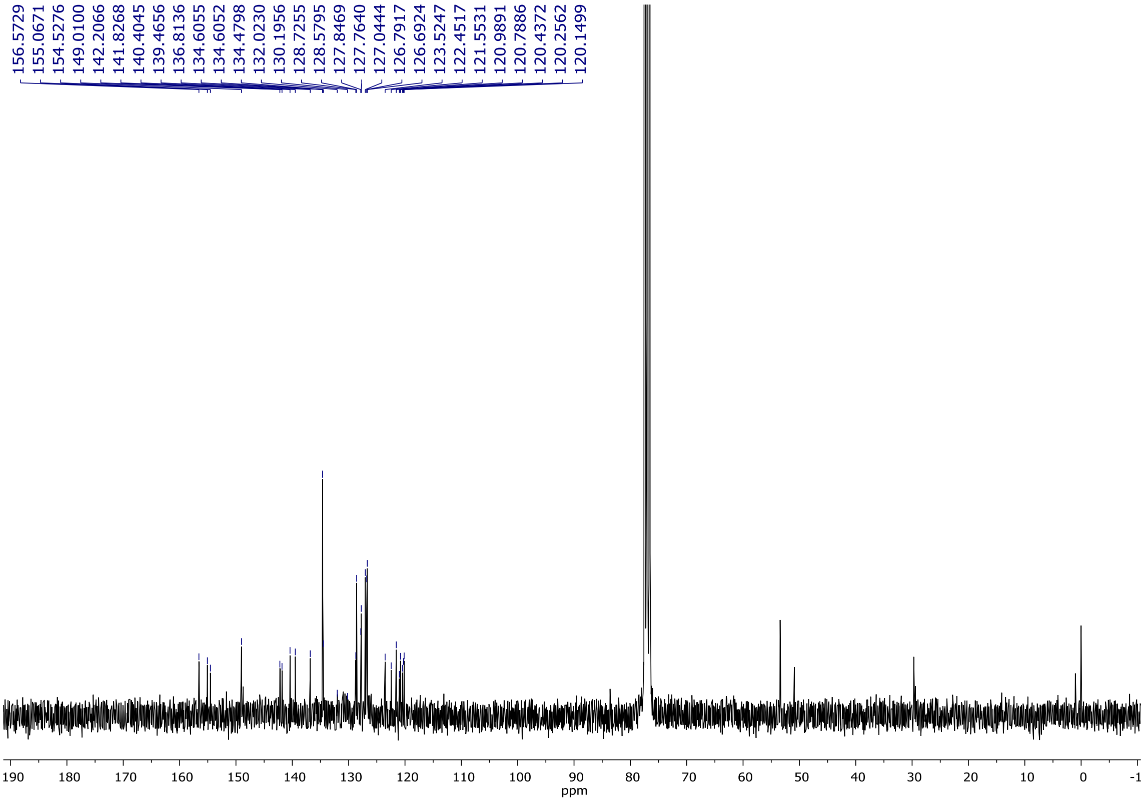

Figure S3. ^{13}C NMR spectrum of compound **2a** in $CDCl_3$.

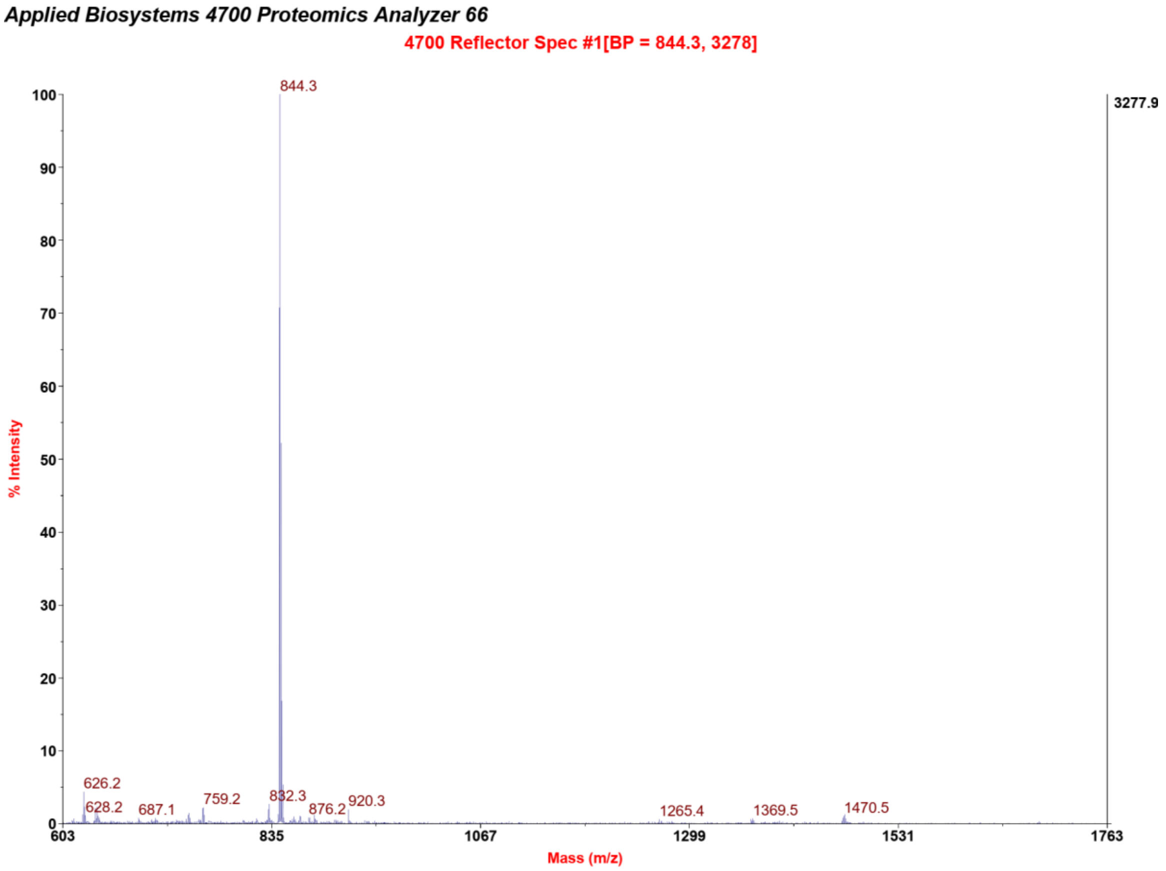

Figure S4. MS MALDI spectrum of compound **2a**.

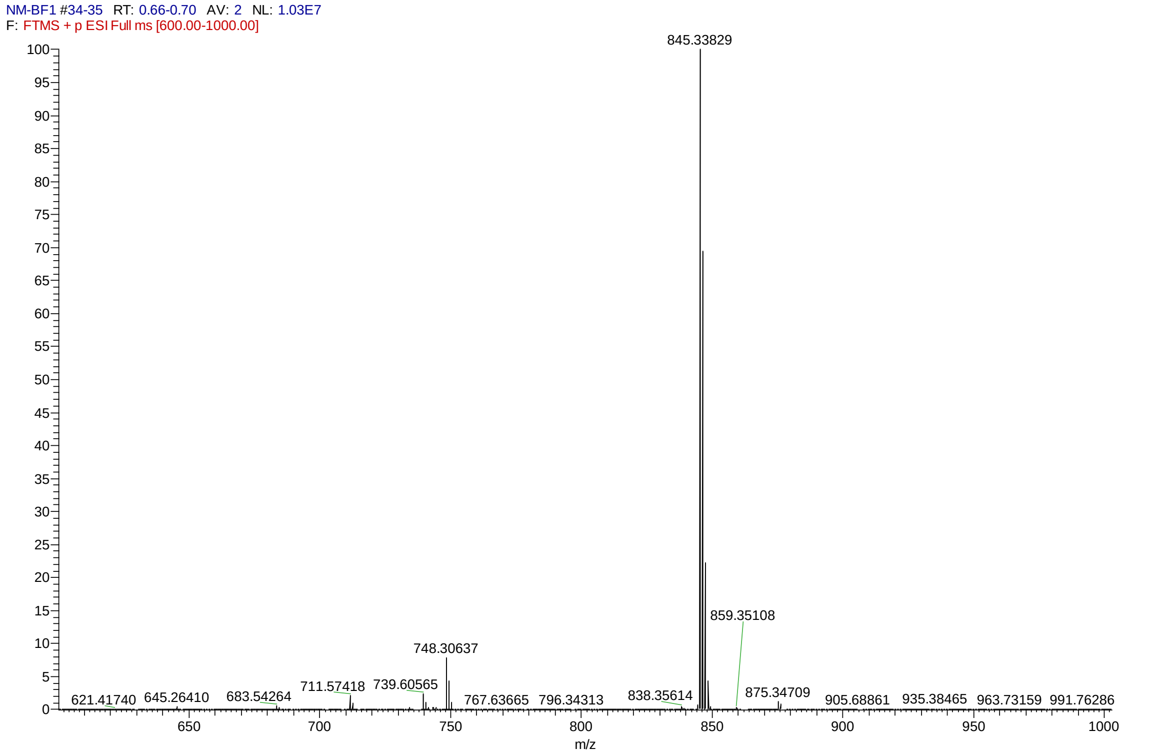

Figure S5. HRMS-ESI(+) spectrum of compound **2a**.

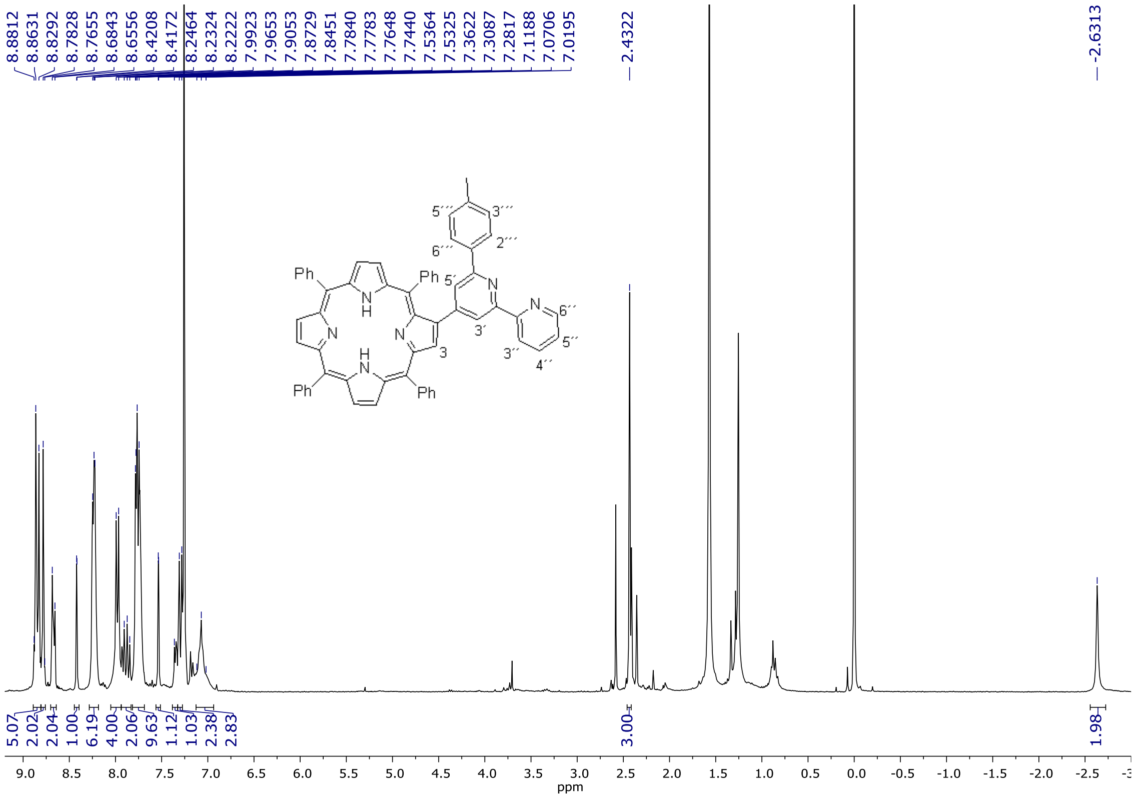

Figure S6. ^{1}H NMR spectrum of compound **2b** in $CDCl_3$.

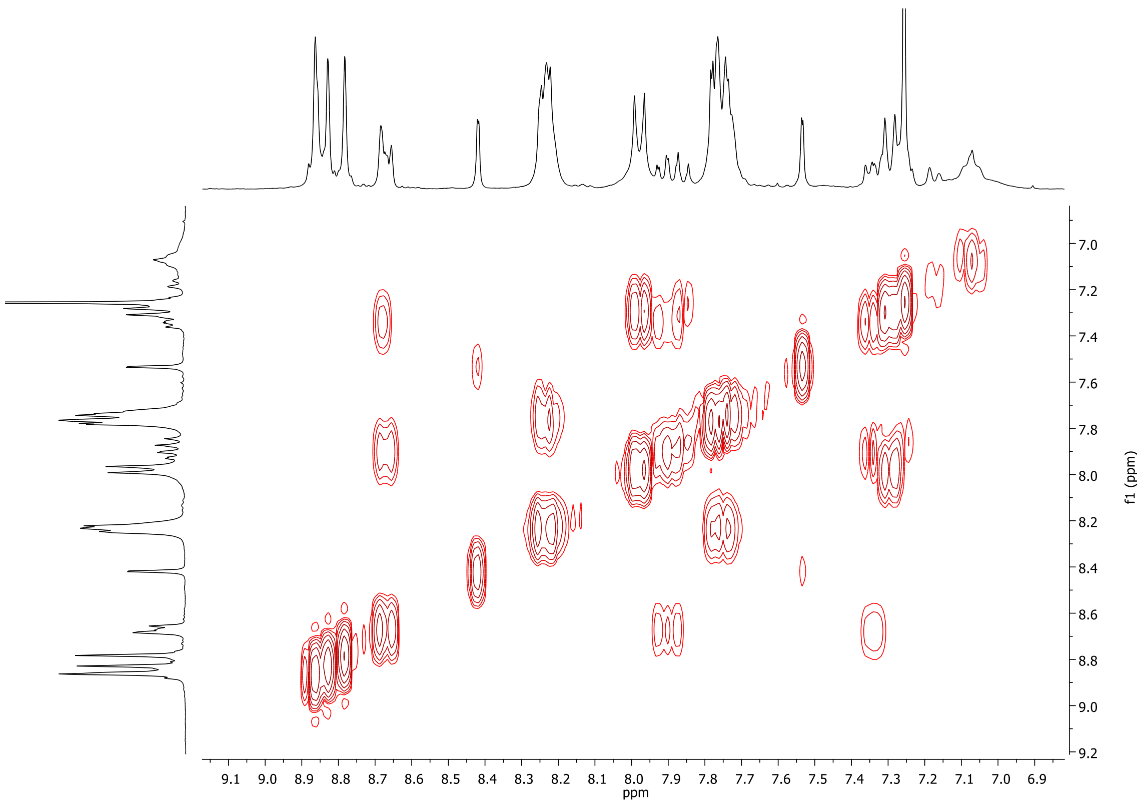

Figure S7. Partial COSY (^{1}H/^{1}H) spectrum of Compound **2b** in $CDCl_3$.

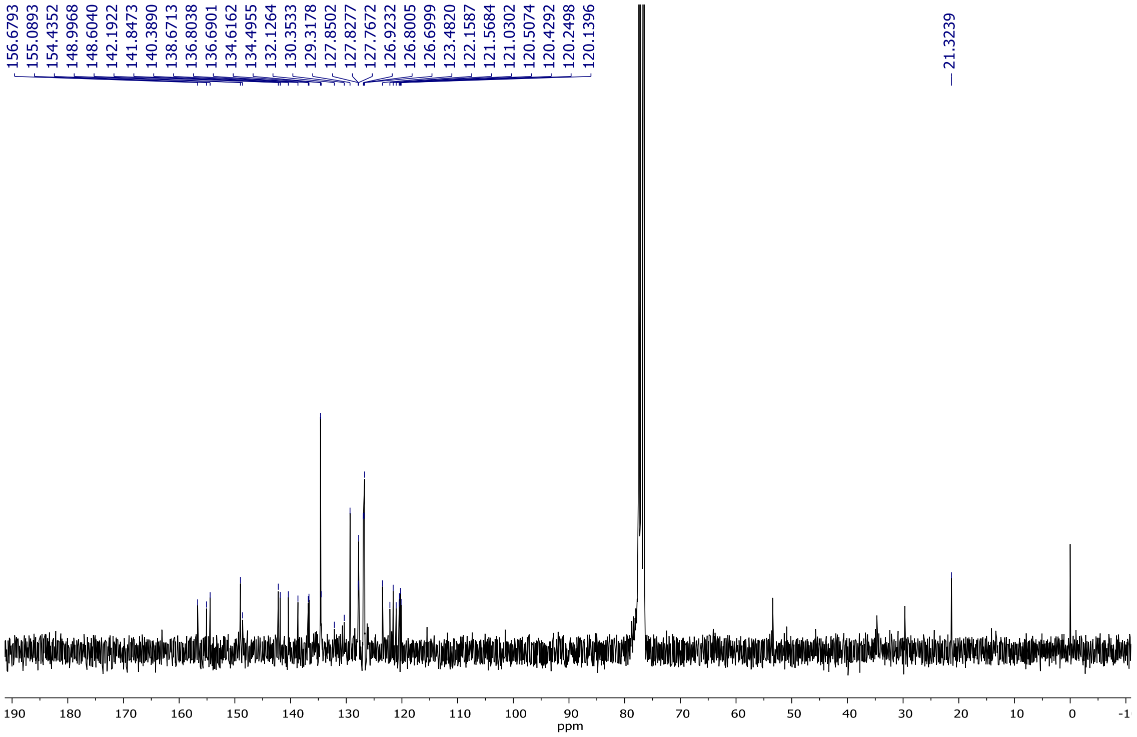

Figure S8. ^{13}C NMR spectrum of compound **2b** in $CDCl_3$.

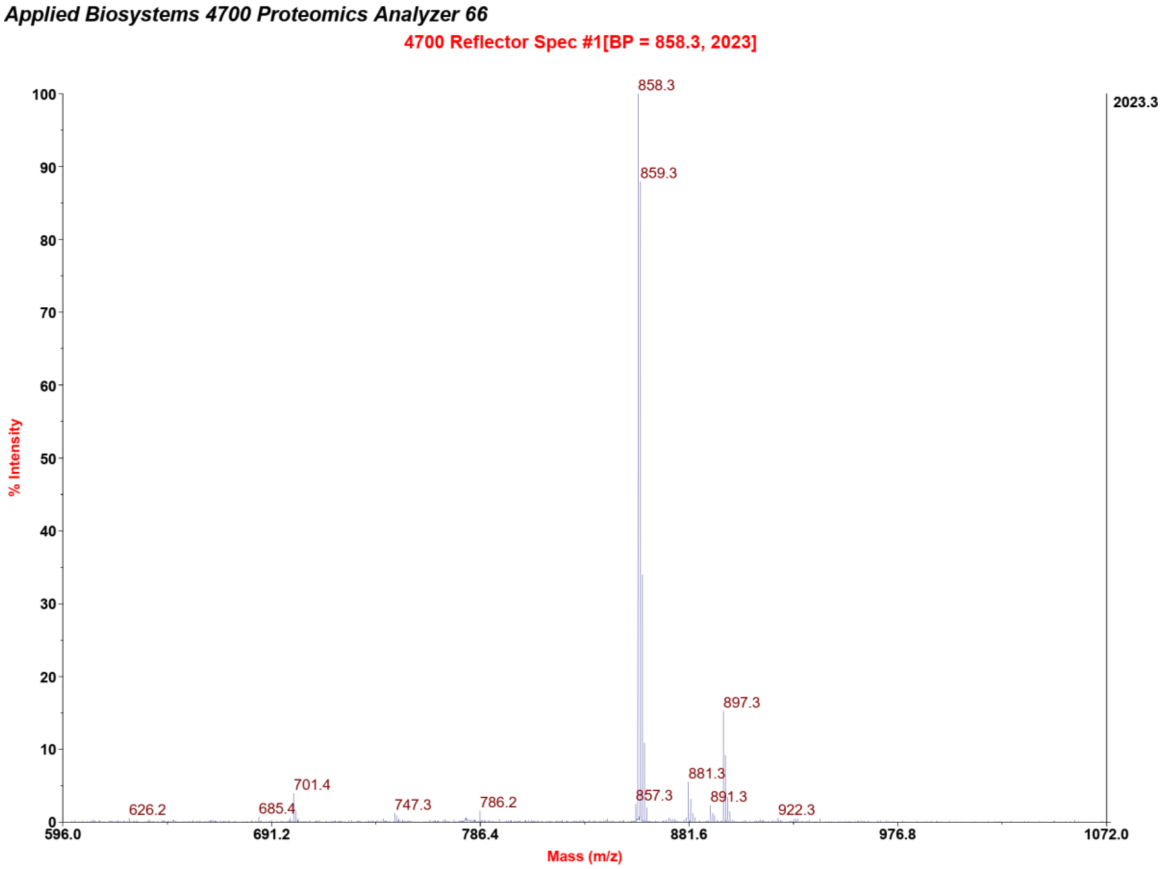

Figure S9. MS MALDI spectrum of compound **2b**.

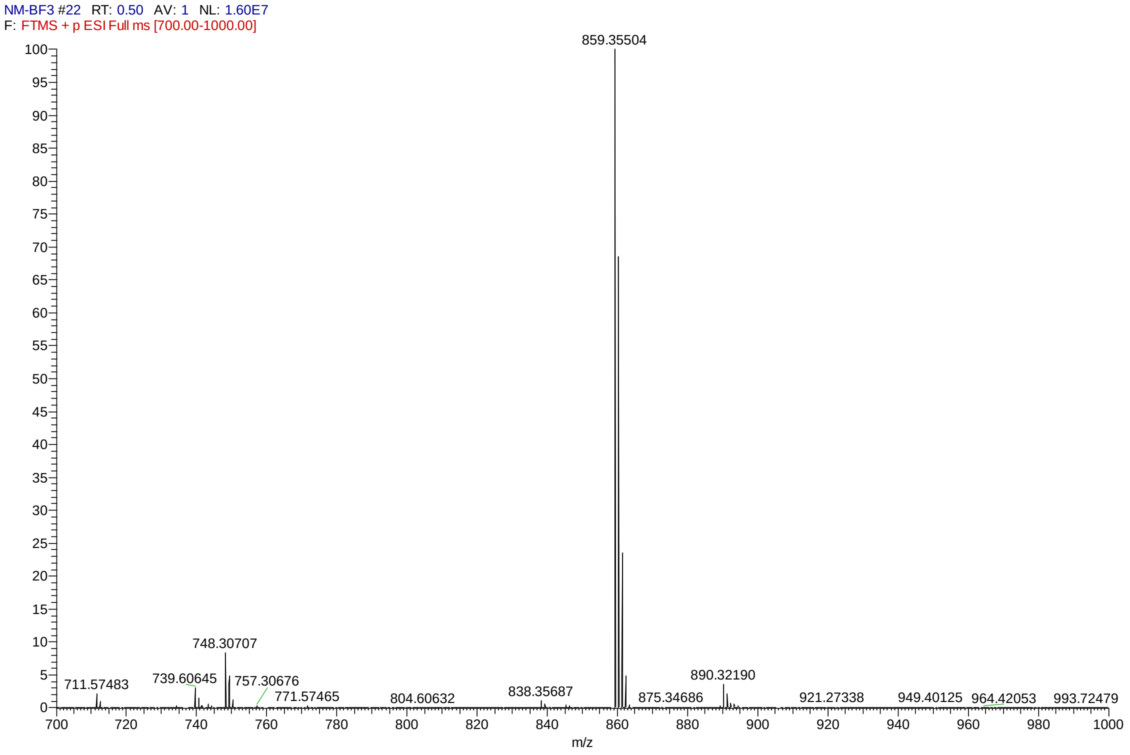

Figure S10. HRMS-ESI(+) spectrum of compound **2b**.

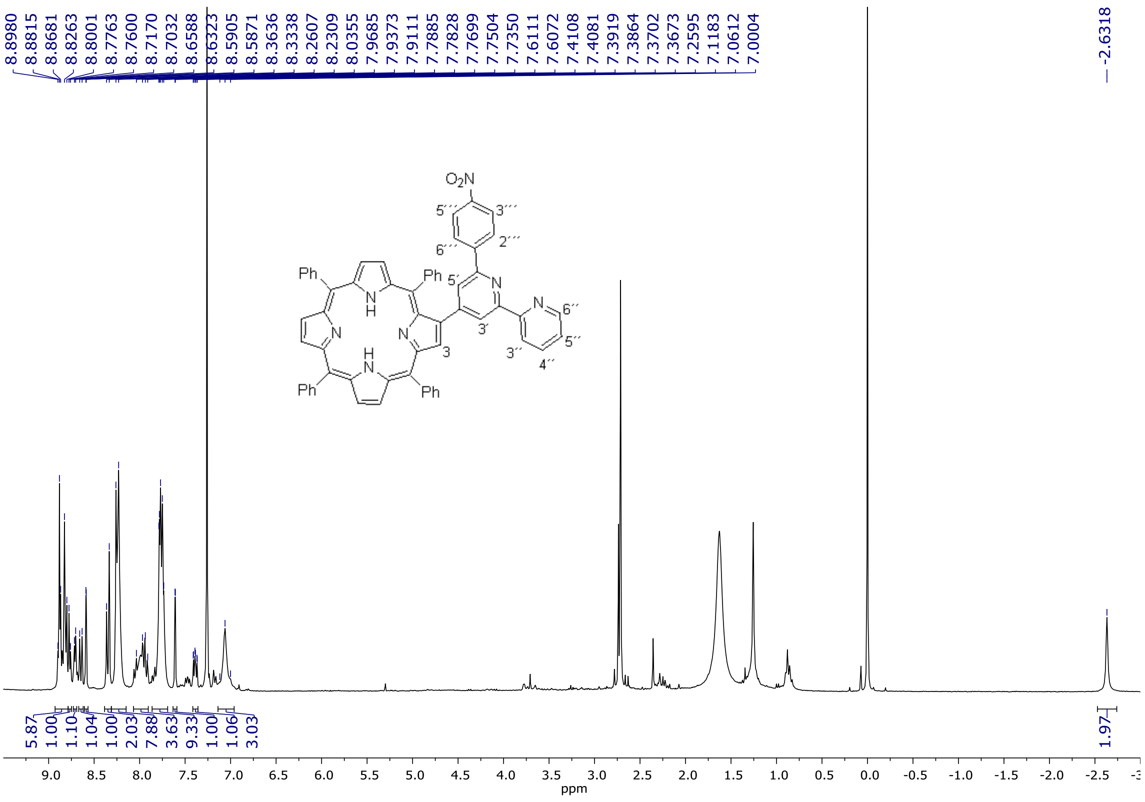

Figure S11. ^{1}H NMR spectrum of compound **3c** in $CDCl_3$.

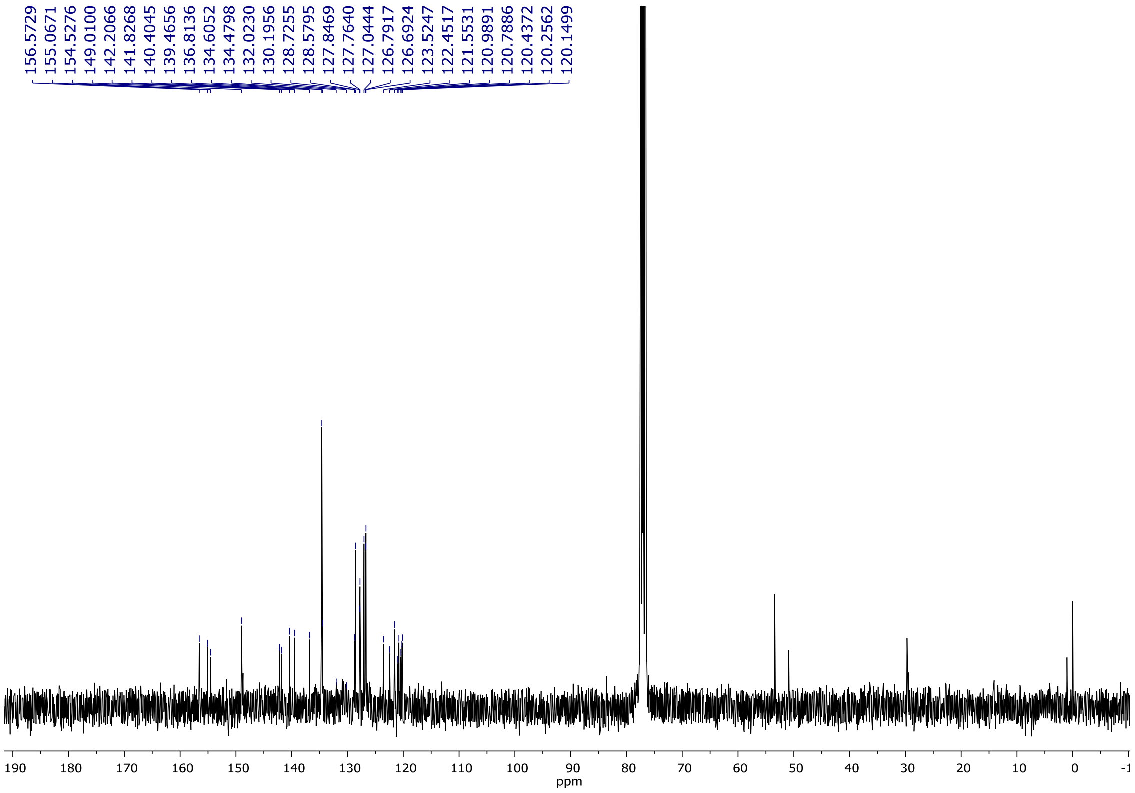

Figure 12. ^{13}C NMR spectrum of compound **2c** in $CDCl_3$.

Applied Biosystems 4700 Proteomics Analyzer 66

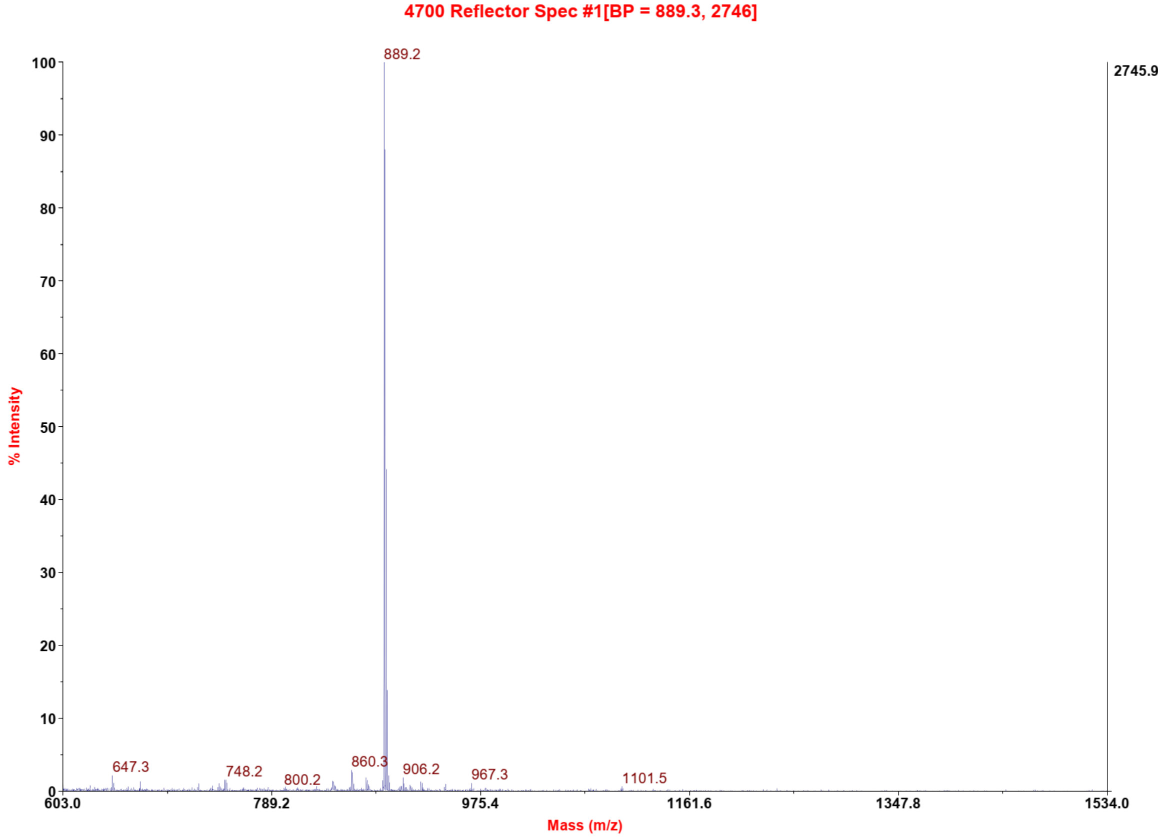

Figure S13. MS MALDI spectrum of compound **2c**.

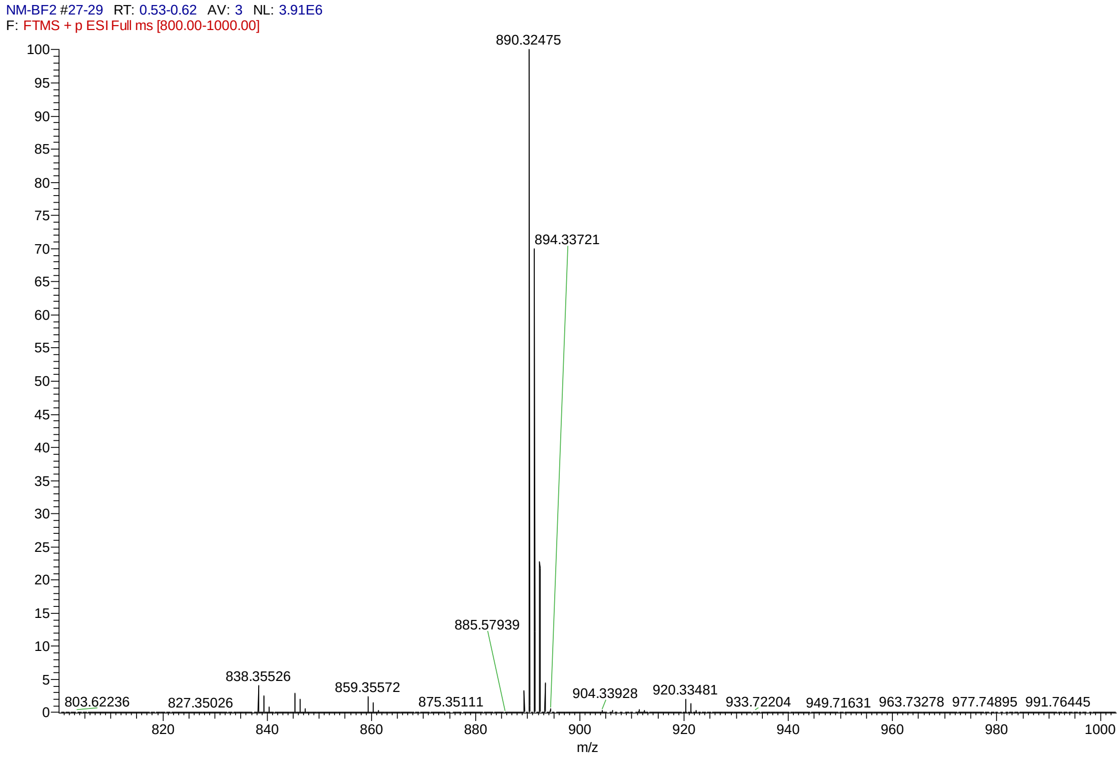

Figure S14. HRMS-ESI(+) spectrum of compound **2c**.

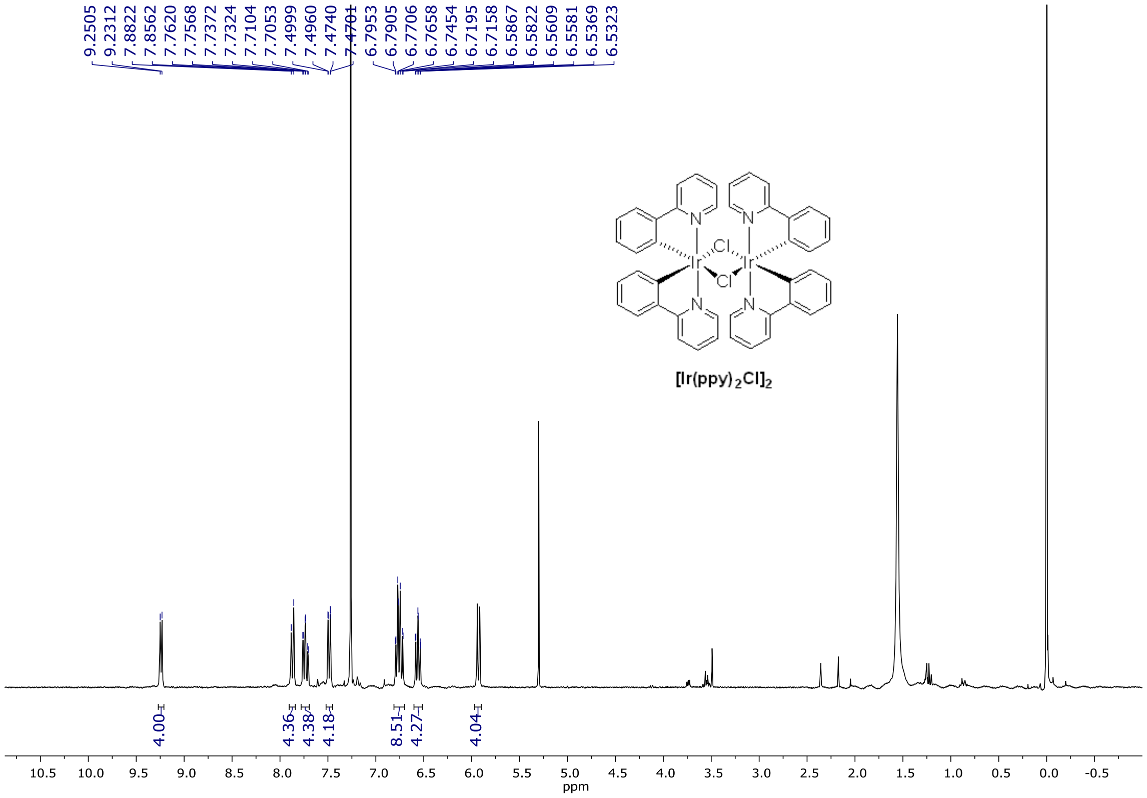

Figure S15. ^{1}H NMR spectrum of $[Ir(ppy)_2Cl]_2$ in $CDCl_3$.

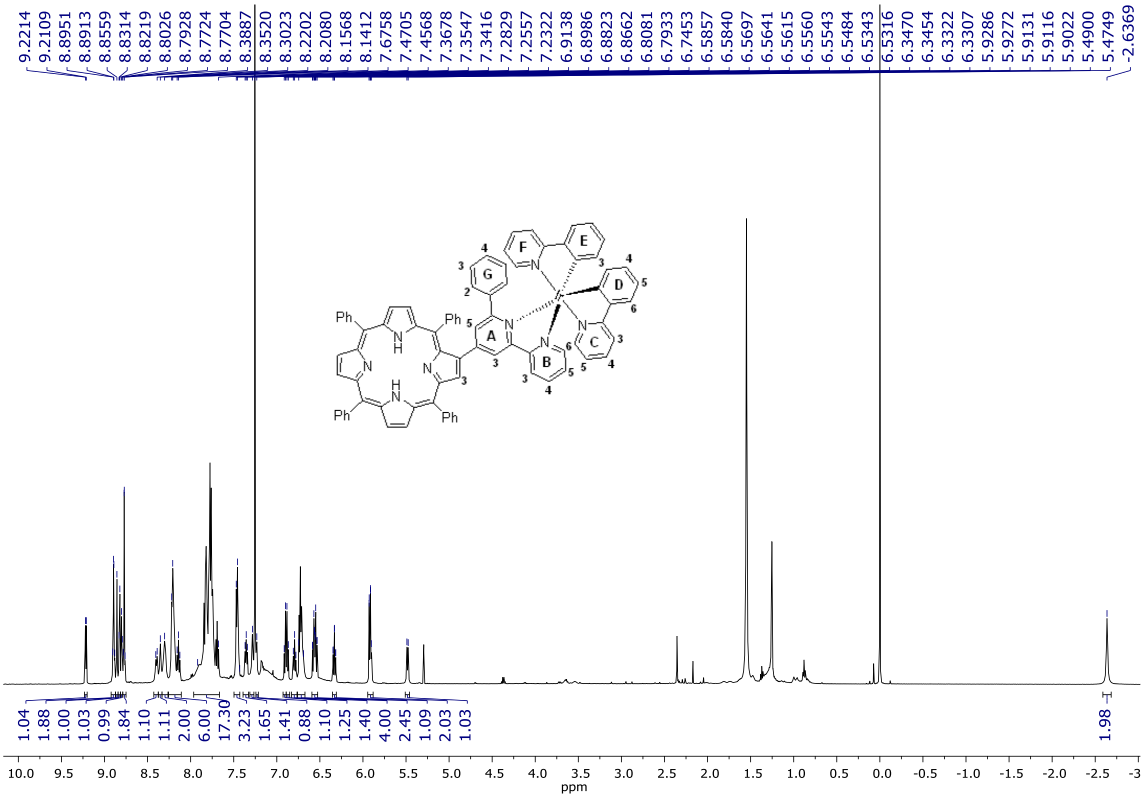

Figure S16. ^{1}H NMR spectrum of compound **4a** in $CDCl_3$.

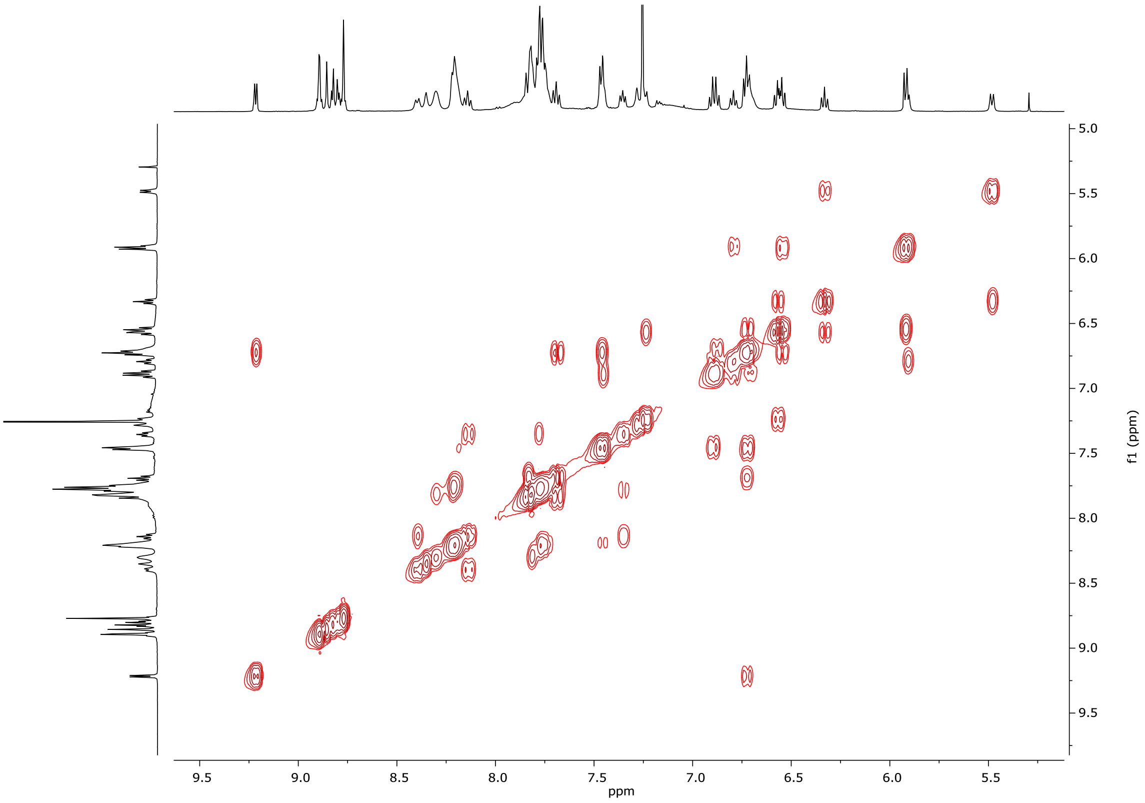

Figure S17. Partial COSY ($^{1}H/^{1}H$) spectrum of Compound **4a** in $CDCl_3$.

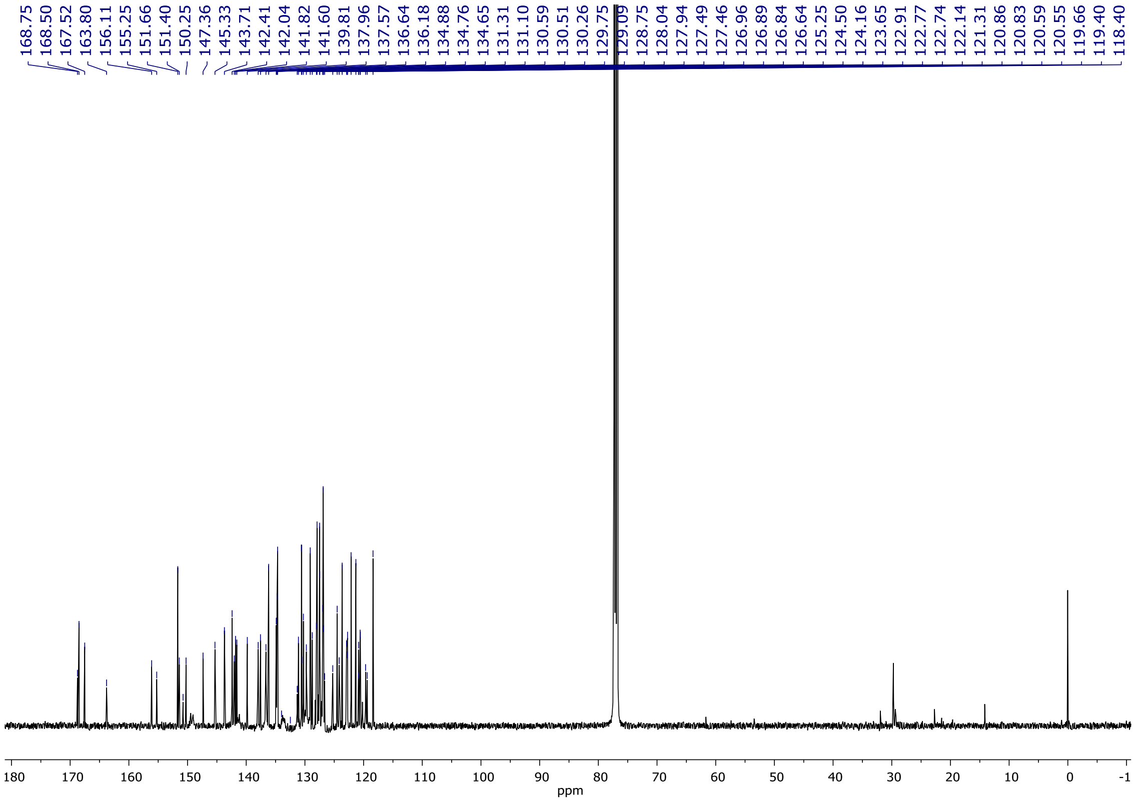

Figure S18. ^{13}C NMR spectrum of compound **4a** in $CDCl_3$.

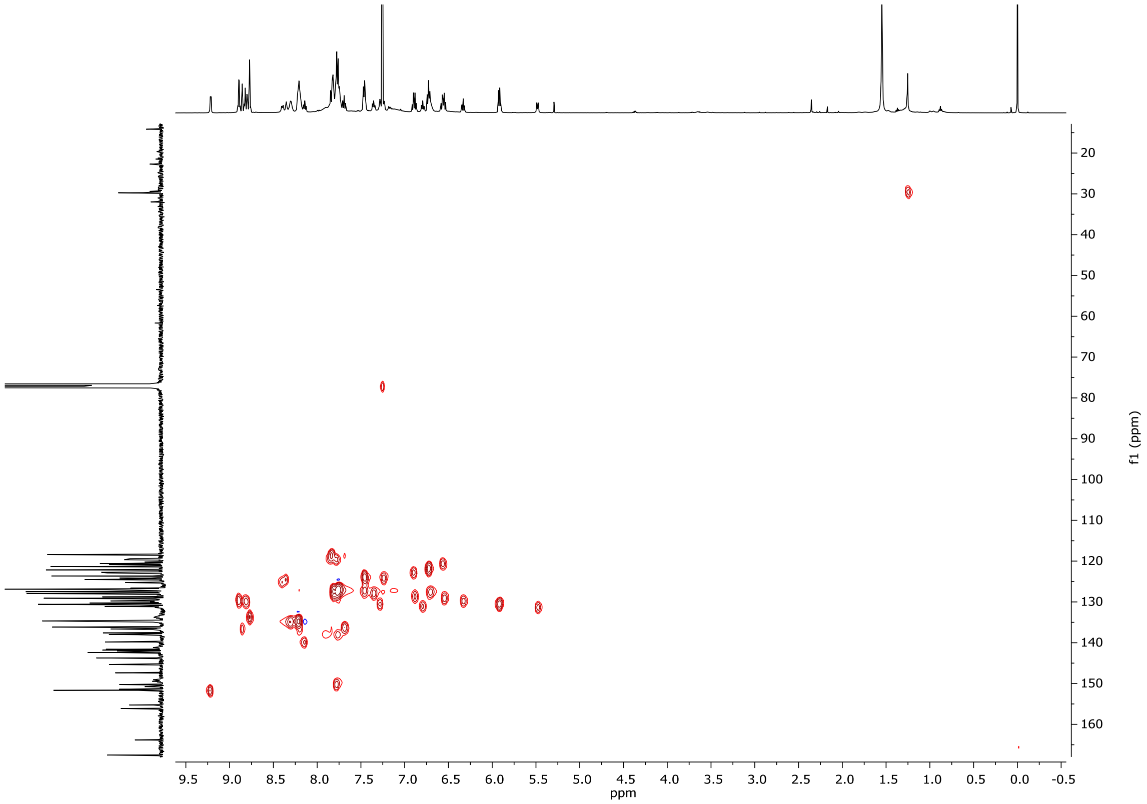

Figure S19. HSQC (^{1}H/^{13}C) spectrum of compound **4a** in $CDCl_3$.

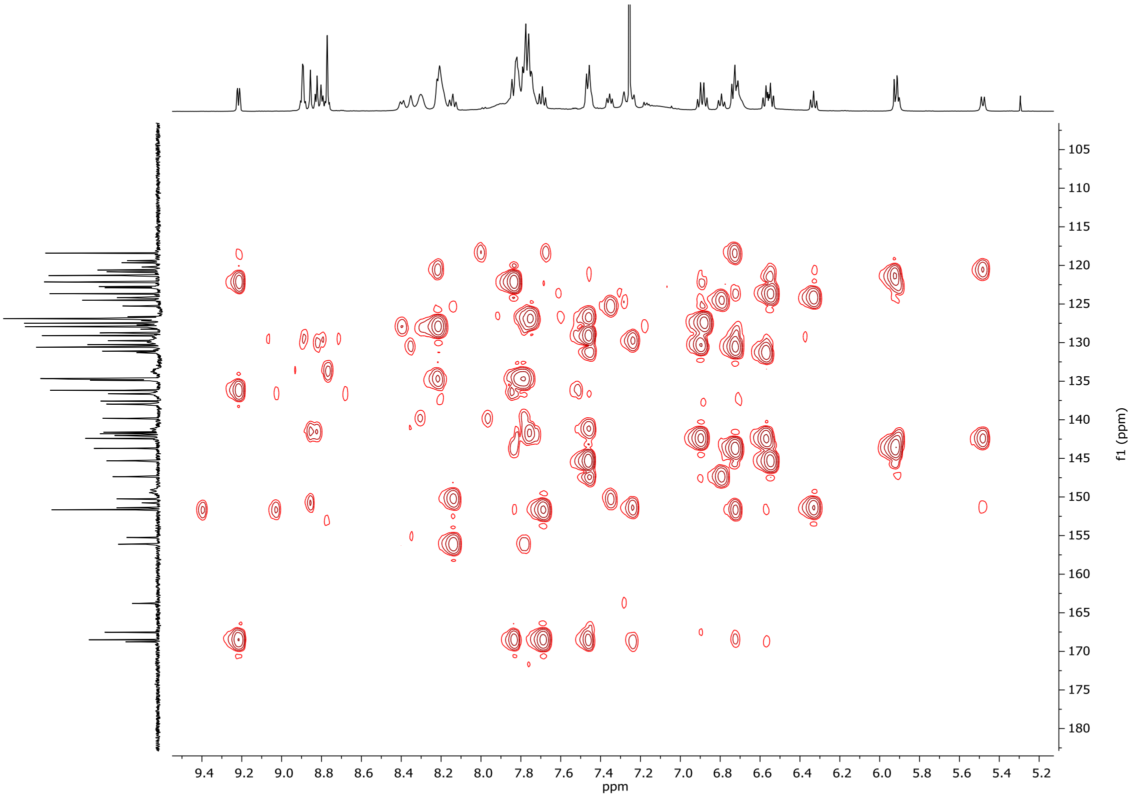

Figure S20. Partial HMBC ($^{1}H/^{13}C$) spectrum of compound **4a** in $CDCl_3$.

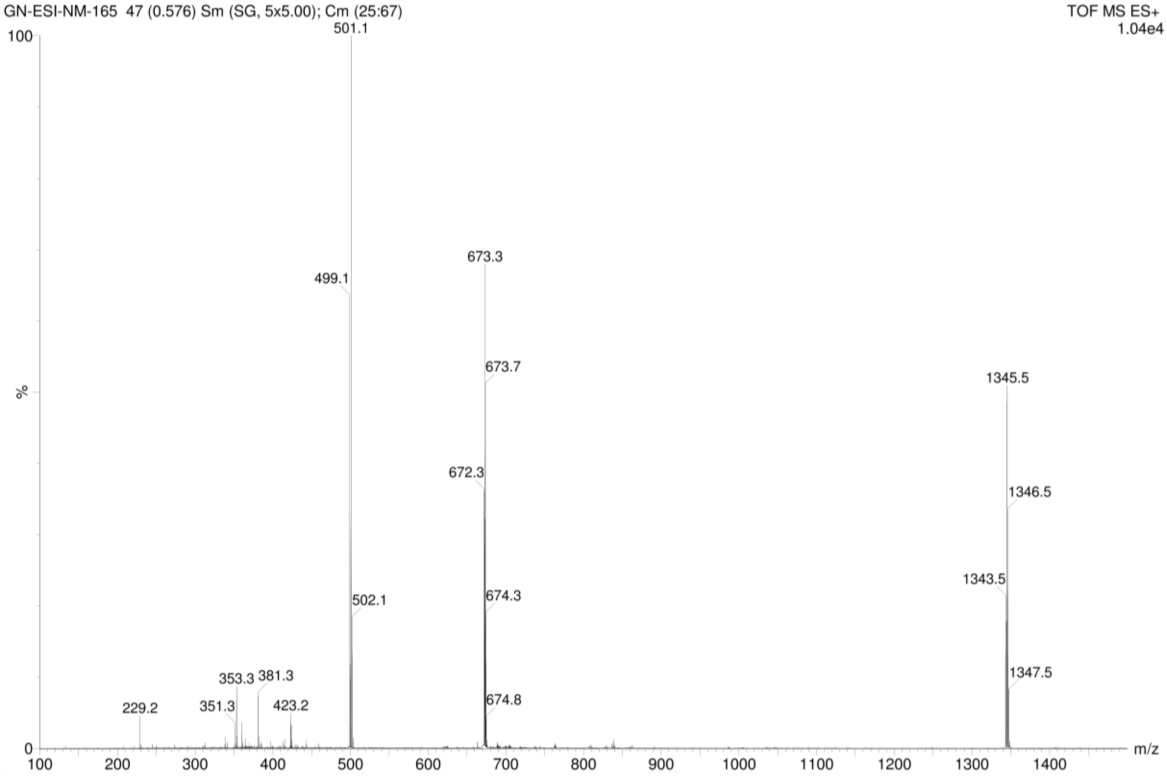

Figure S21. ESI(+)-MS spectrum of compound **4a**.

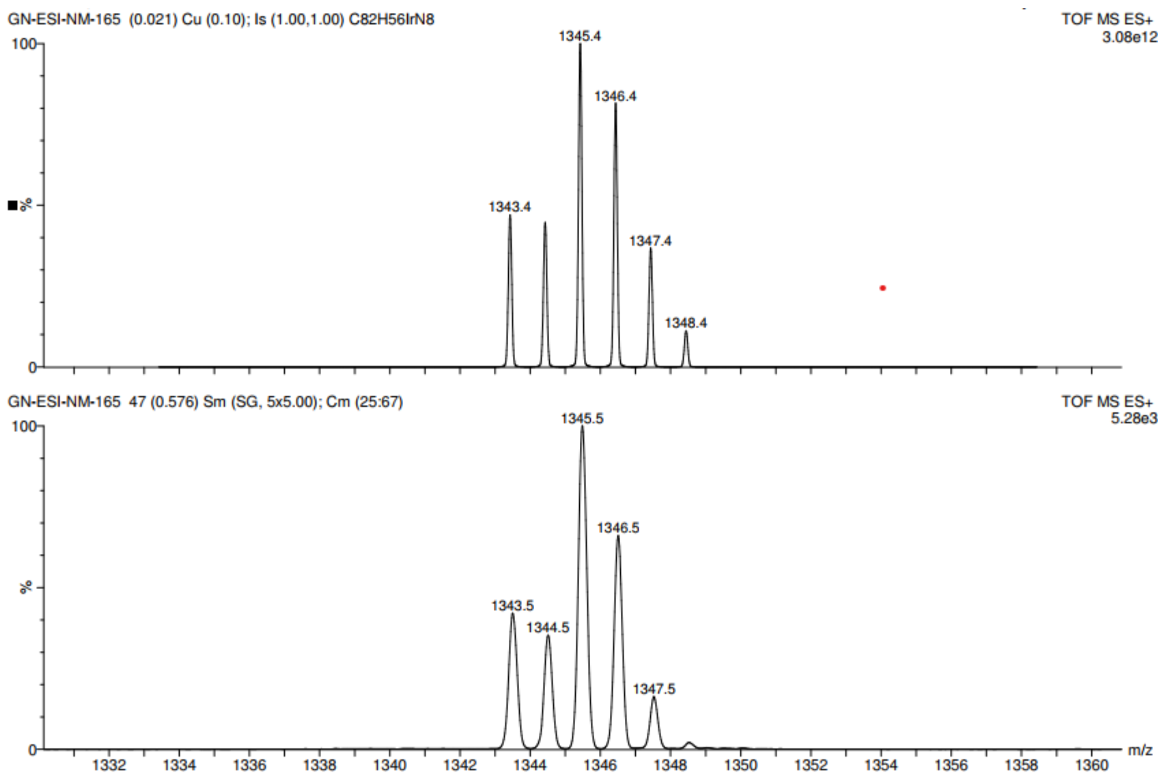

Figure S22. Isotopic pattern corresponding to the $[M]^{+\bullet}$ ion of compound **4a**.

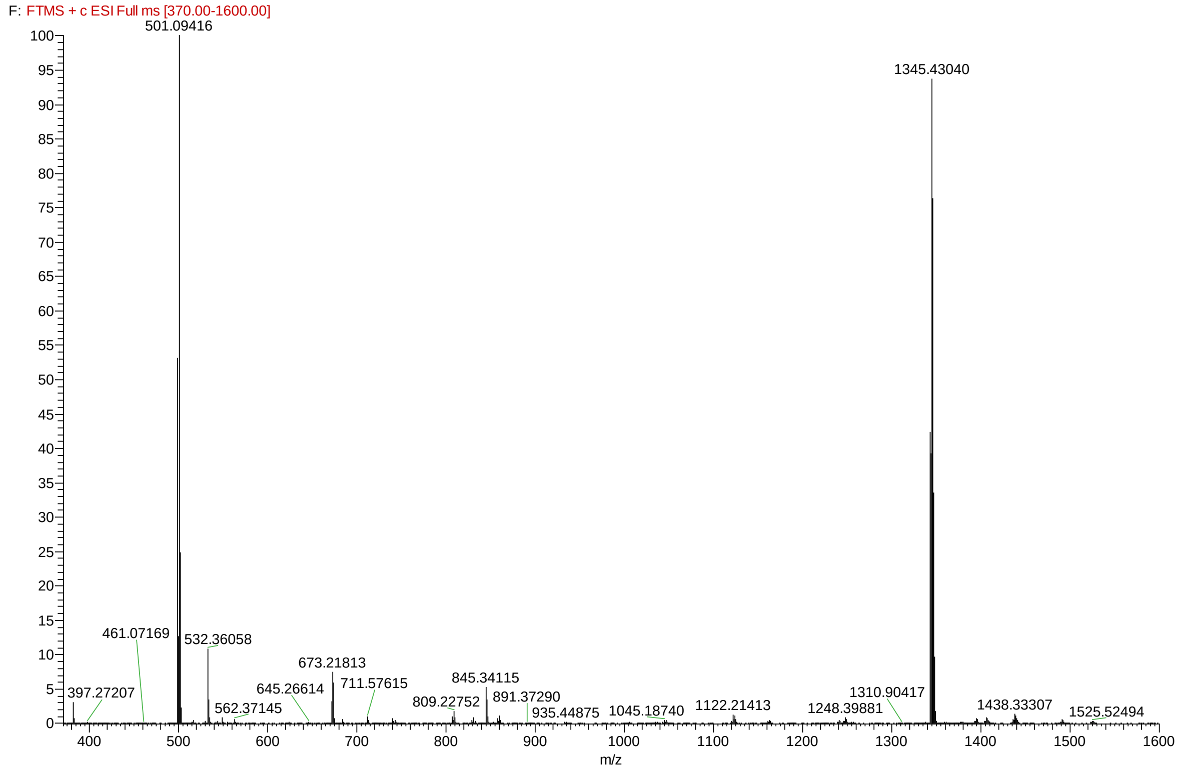

Figure S23. HRMS-ESI(+) spectrum of compound **4a**.

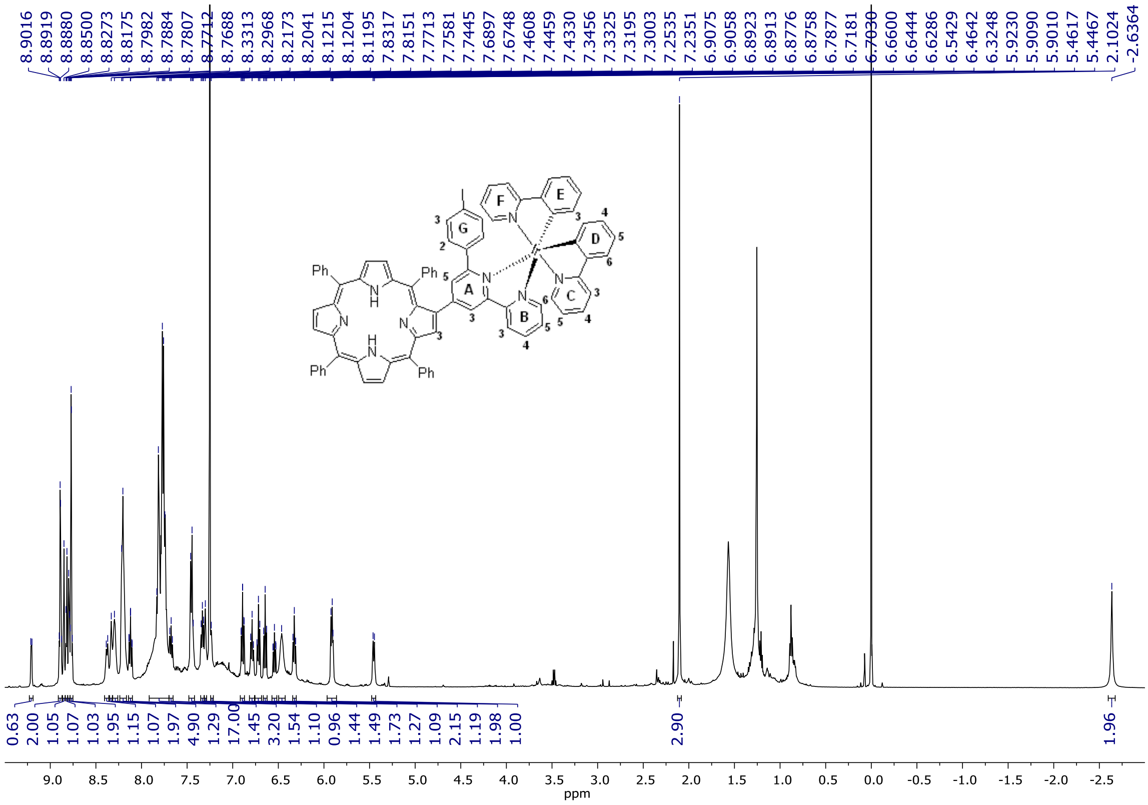

Figure S24. ^{1}H NMR spectrum of compound **4b** in $CDCl_3$.

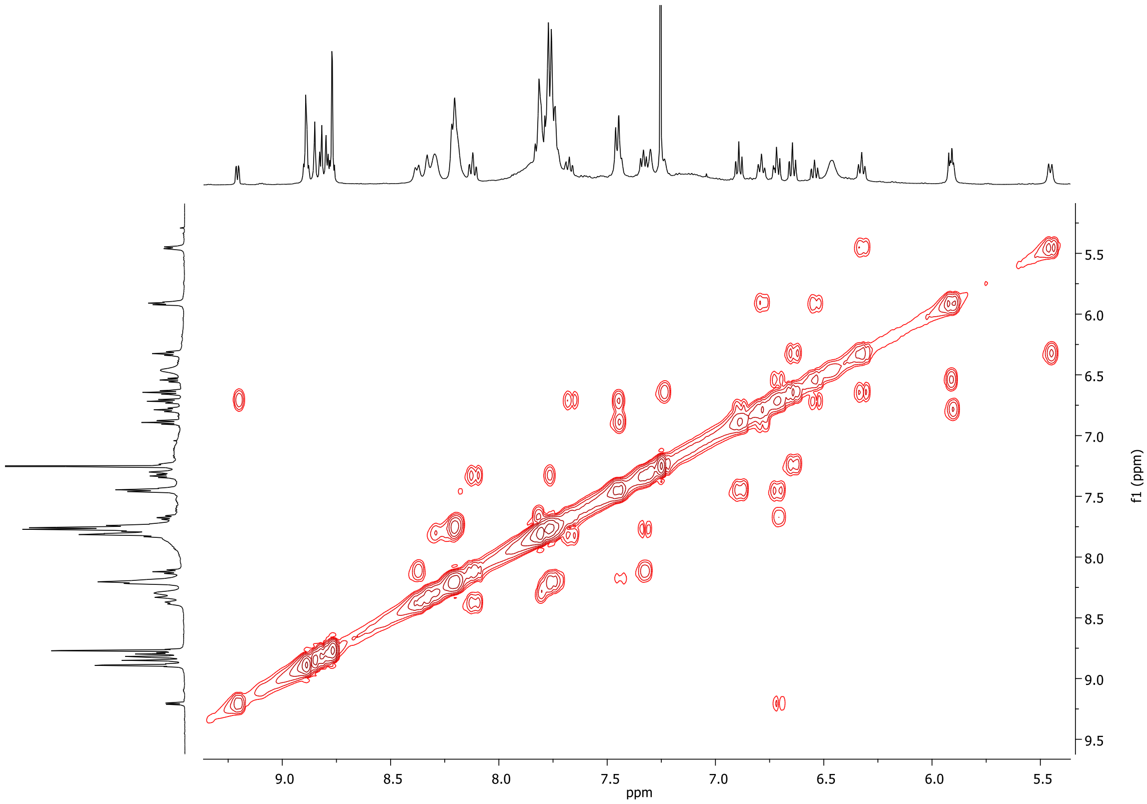

Figure S25. Partial COSY ($^{1}H/^{1}H$) spectrum of compound **4b** in $CDCl_3$.

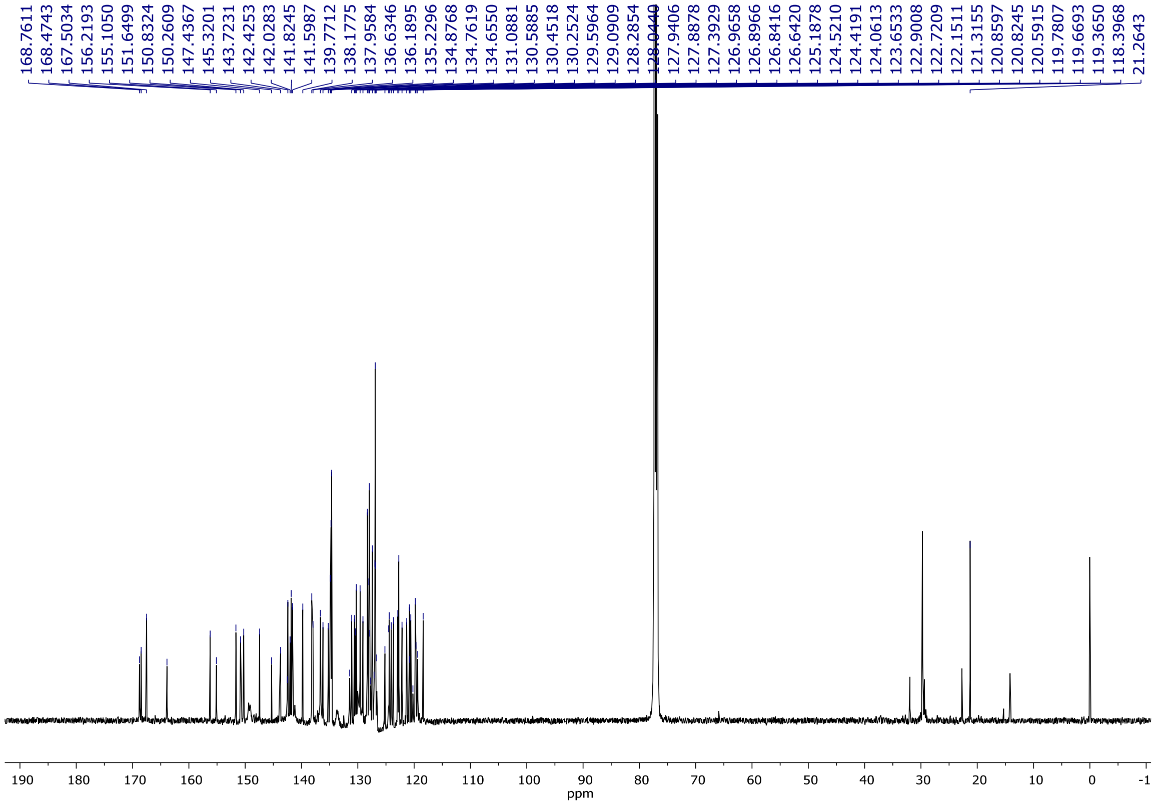

Figure S26. ^{13}C NMR spectrum of compound **4b** in $CDCl_3$.

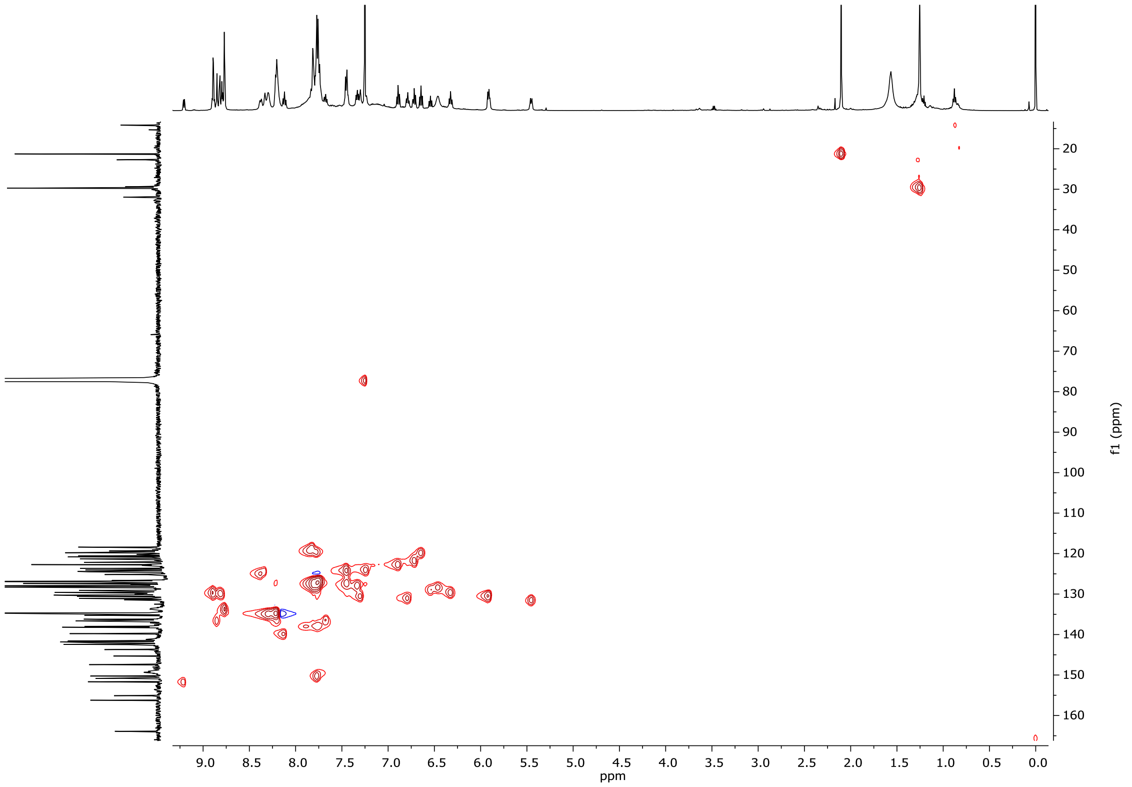

Figure S27. HSQC ($^{1}H/^{13}C$) spectrum of compound **4b** in $CDCl_3$.

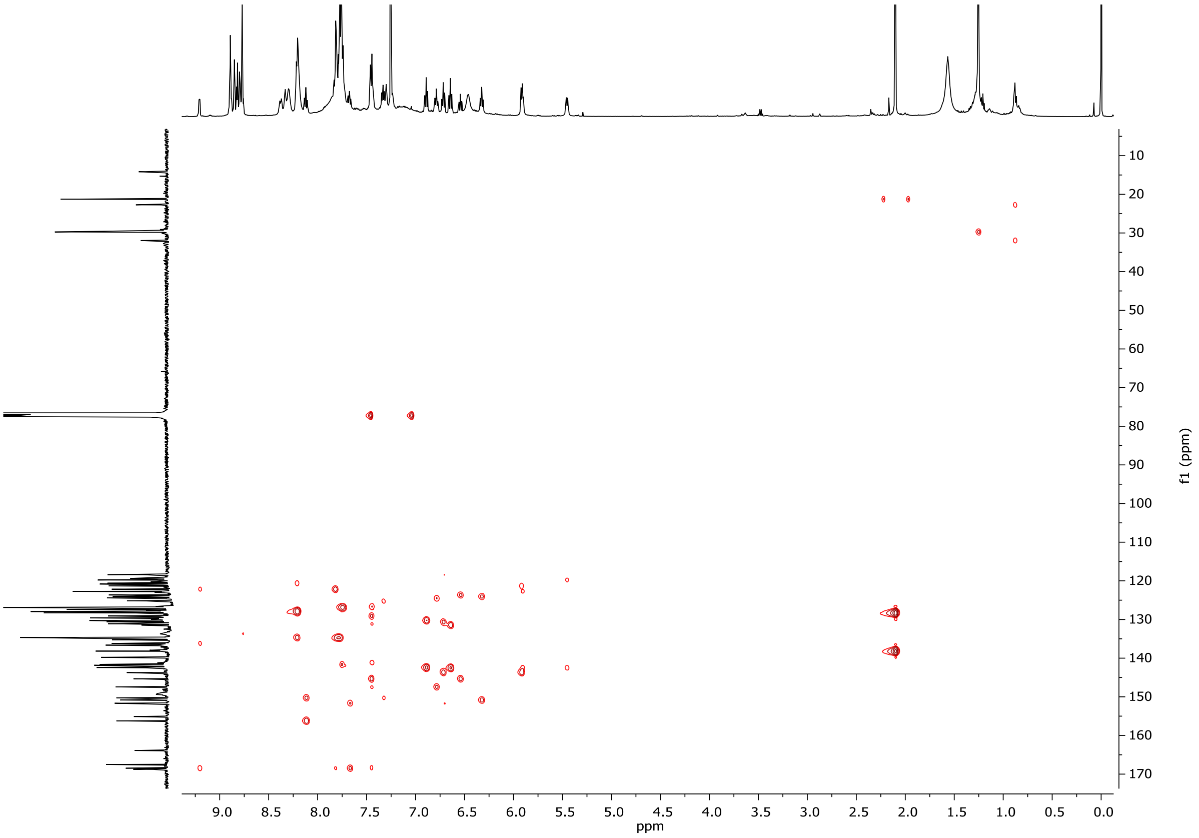

Figure S28. HMBC ($^1H/^{13}C$) spectrum of compound **4b** in $CDCl_3$.

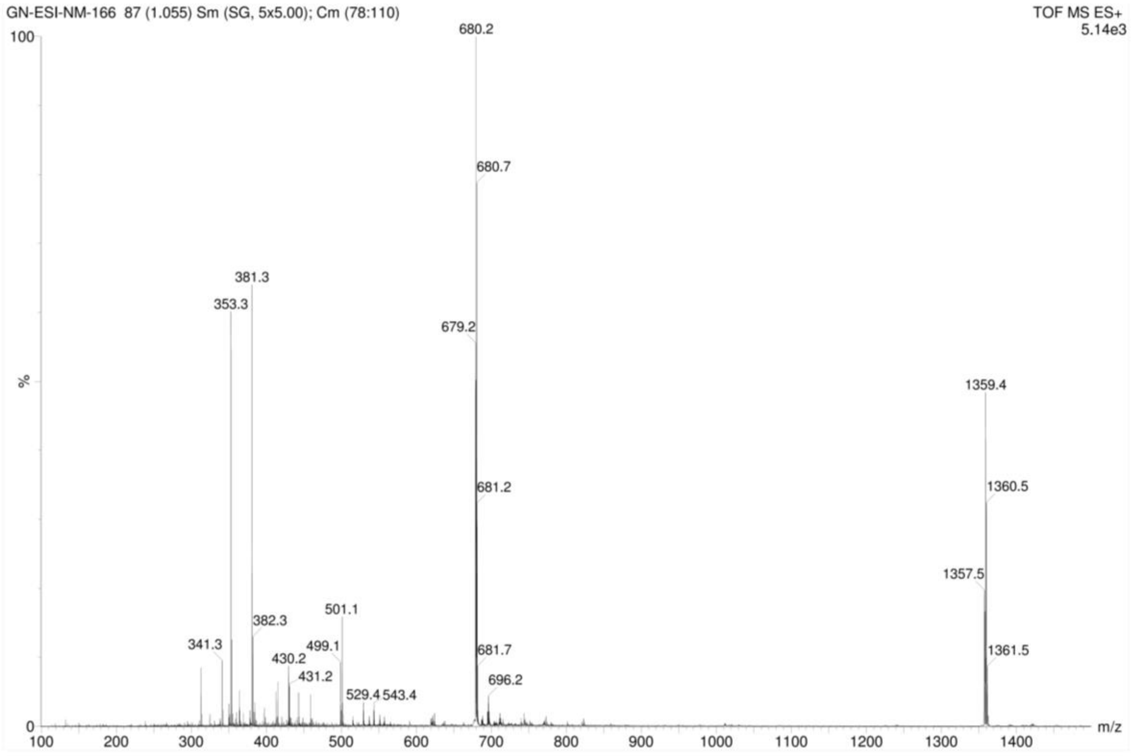

Figure S29. ESI(+)-MS spectrum of compound **4b**.

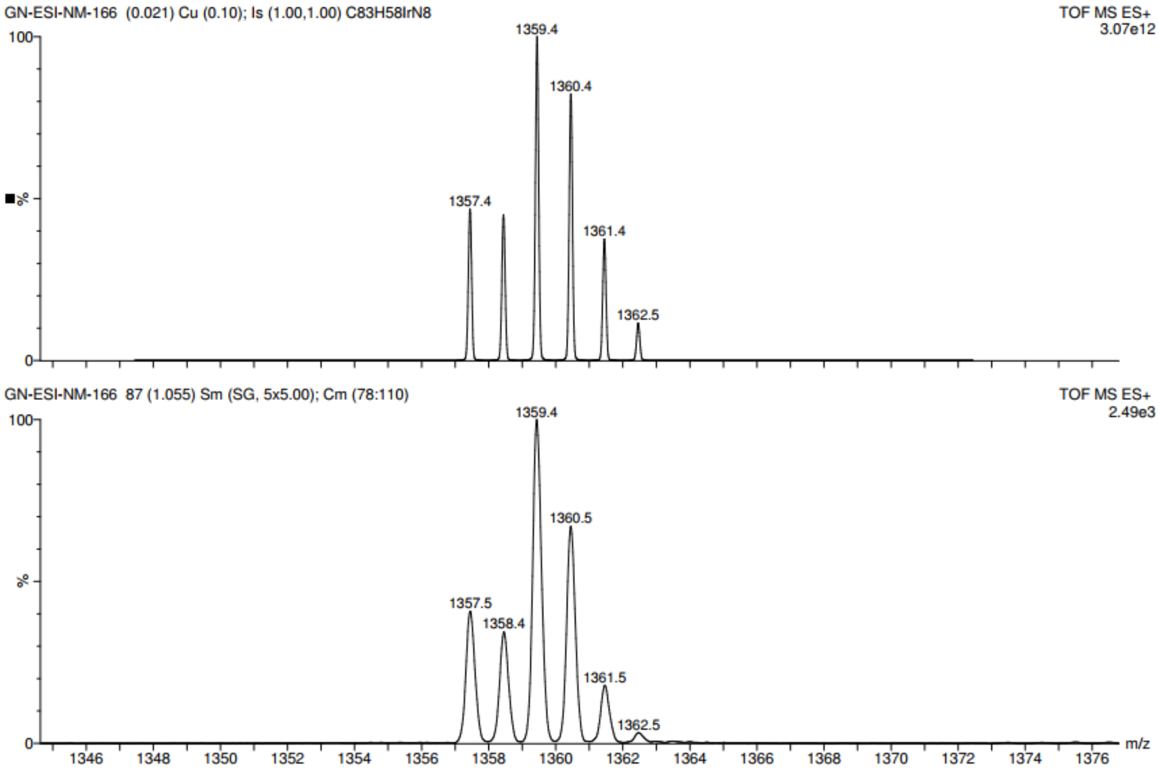

Figure S30. Isotopic pattern corresponding to the $[M]^{+\bullet}$ ion of compound **4b**.

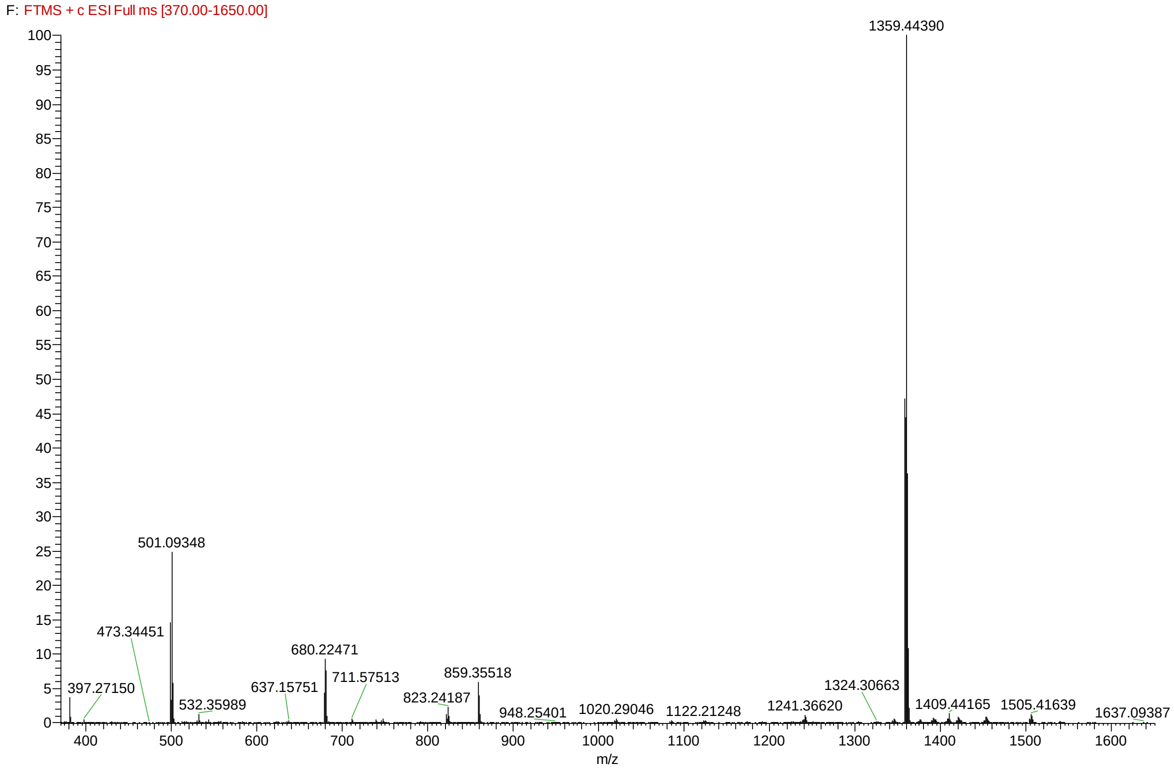

Figure S31. HRMS-ESI(+) spectrum of compound **4b**.

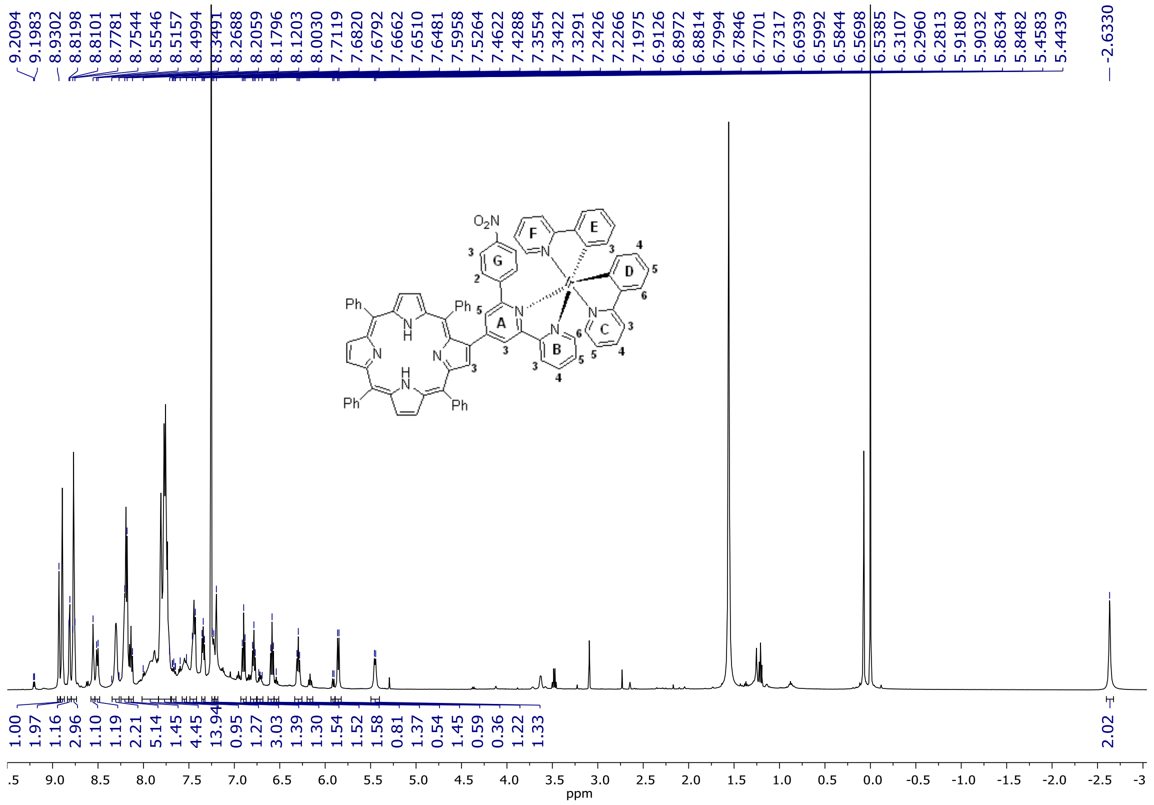

Figure S32. ^{1}H NMR spectrum of compound **4c** in $CDCl_3$.

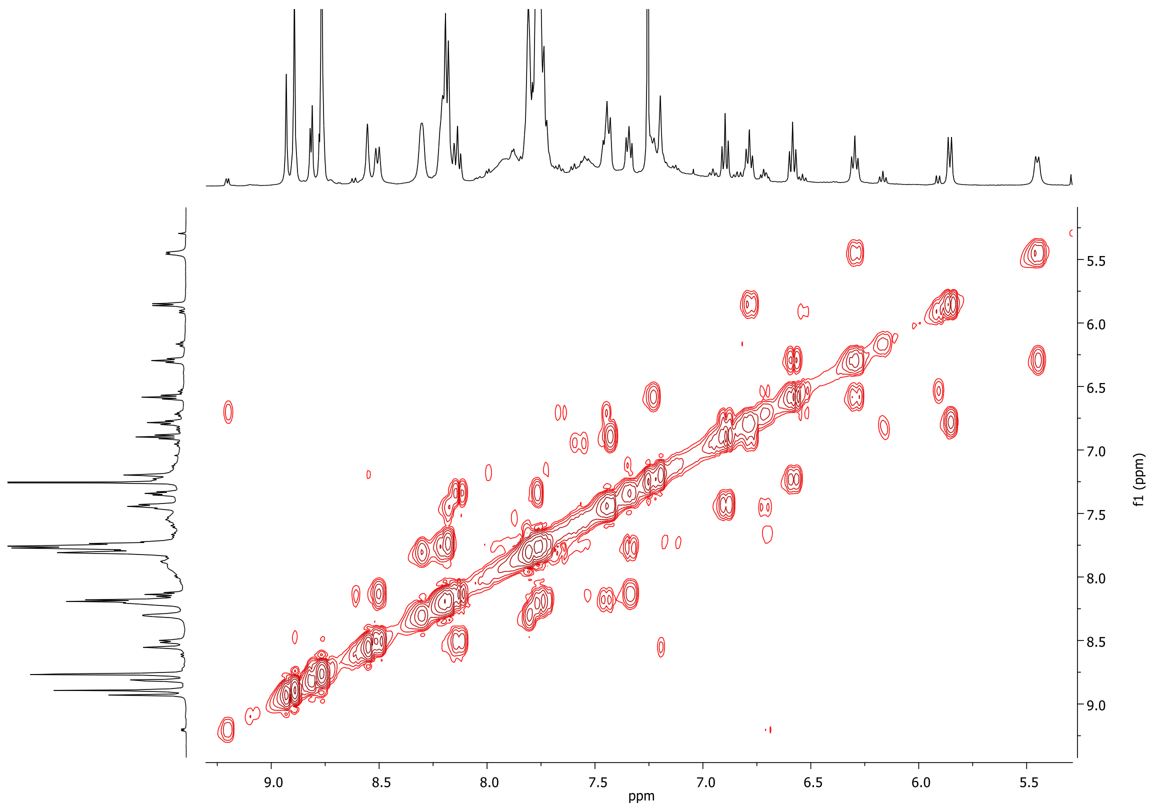

Figure S33. Partial COSY ($^{1}H/^{1}H$) spectrum of compound **4c** in $CDCl_3$.

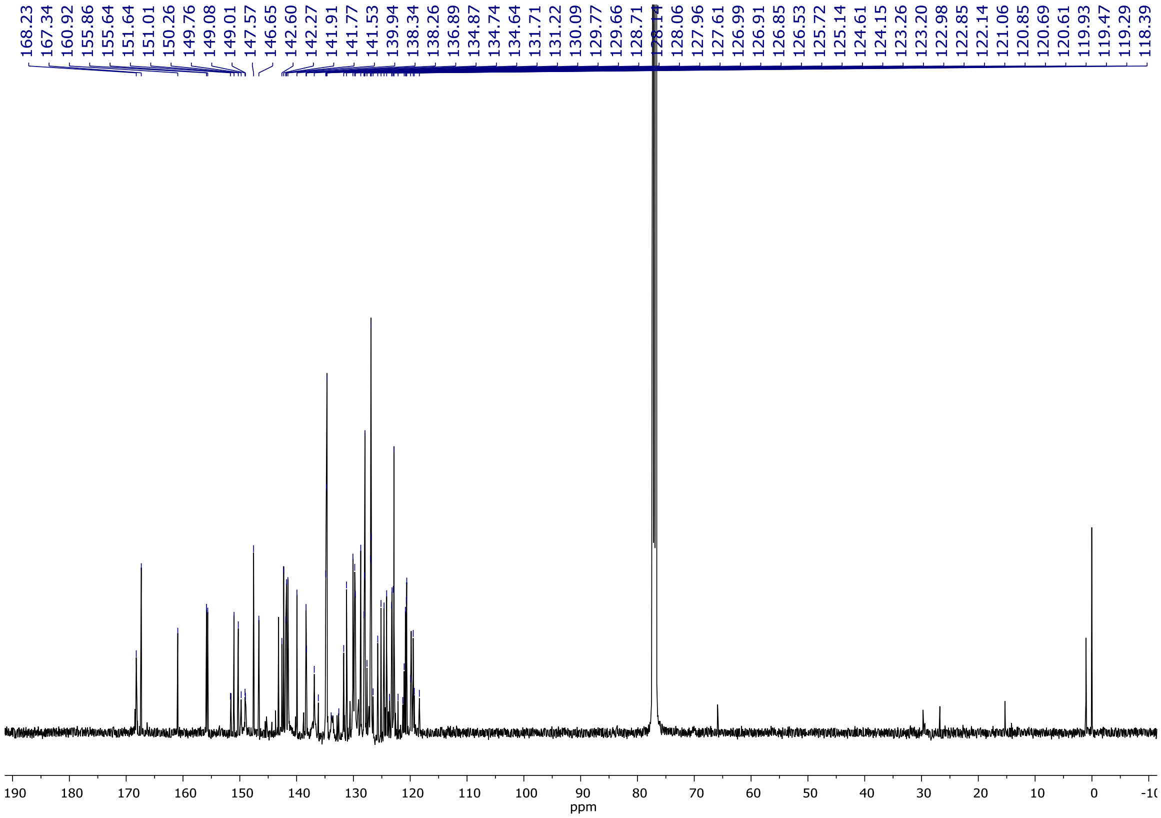

Figure S34. ^{13}C NMR spectrum of compound **4c** in $CDCl_3$.

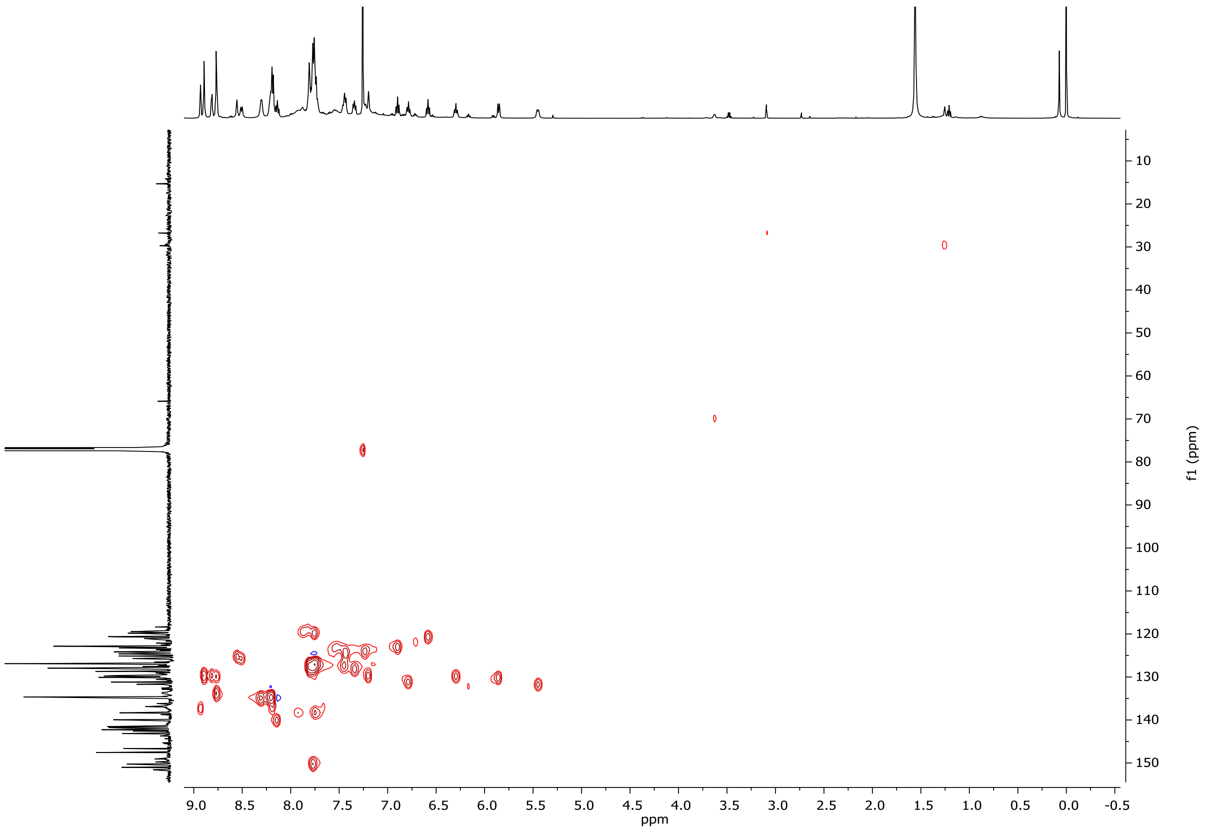

Figure S35. HSQC ($^{1}H/^{13}C$) spectrum of compound **4c** in $CDCl_3$.

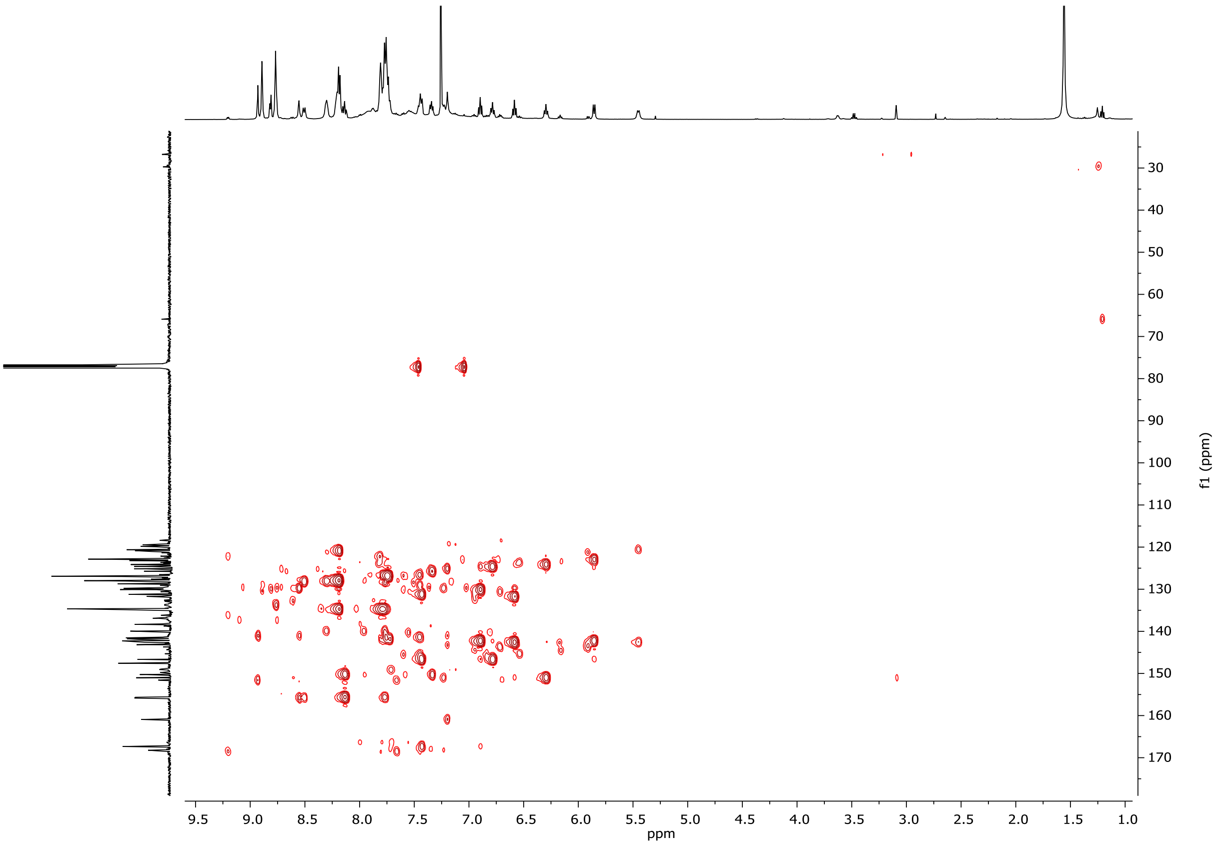

Figure S36. HMBC ($^1H/^{13}C$) spectrum of compound **4c** in $CDCl_3$.

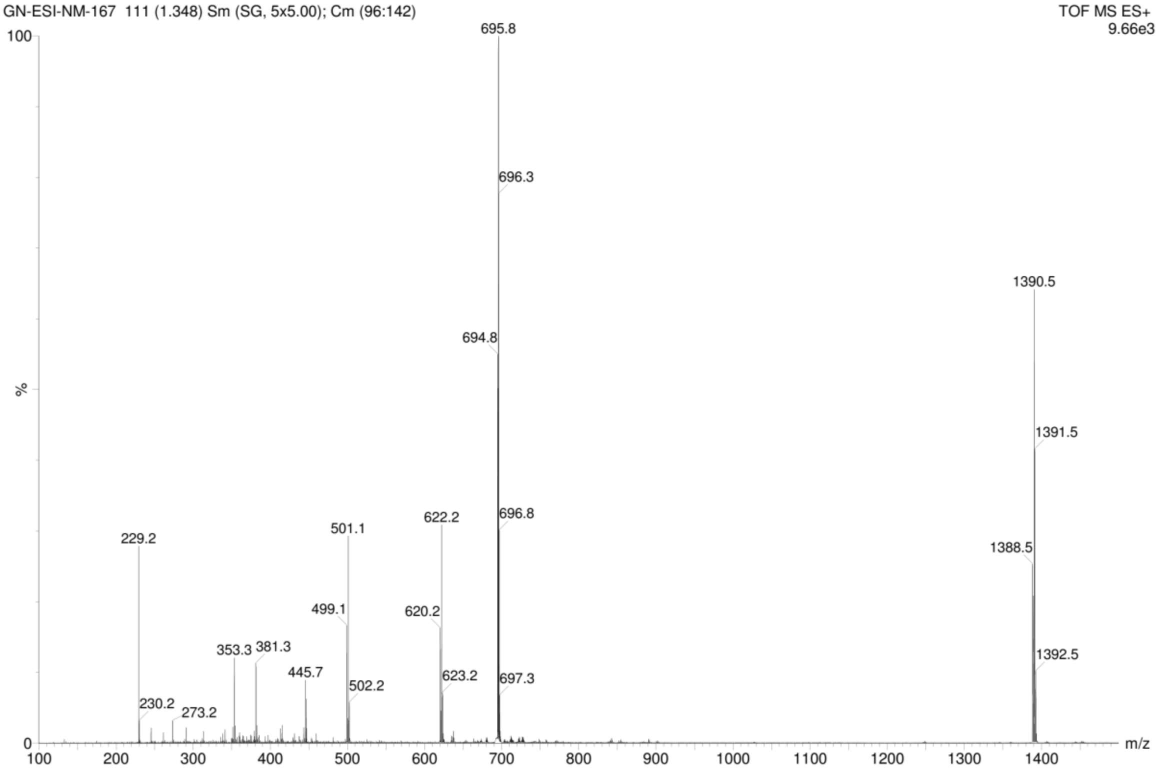

Figure S37. ESI(+)-MS spectrum of compound **4c**.

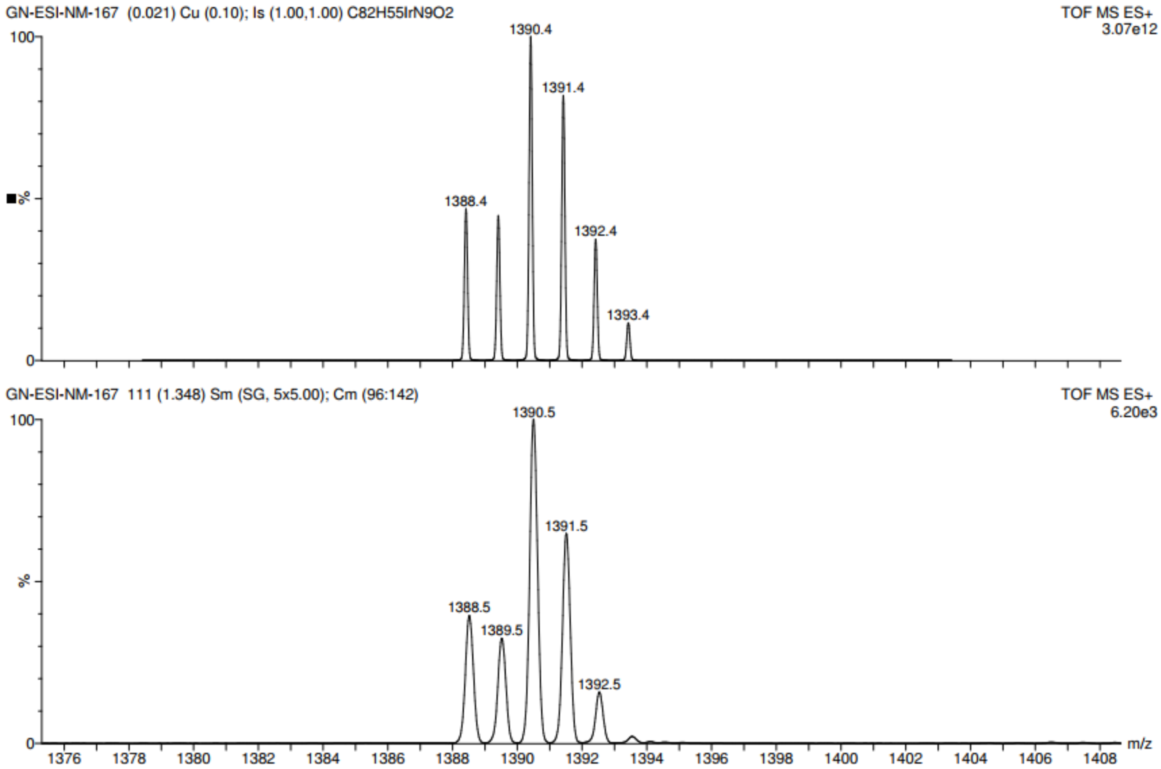

Figure S38. Isotopic pattern corresponding to the $[M]^{+\bullet}$ ion of compound **4c**.

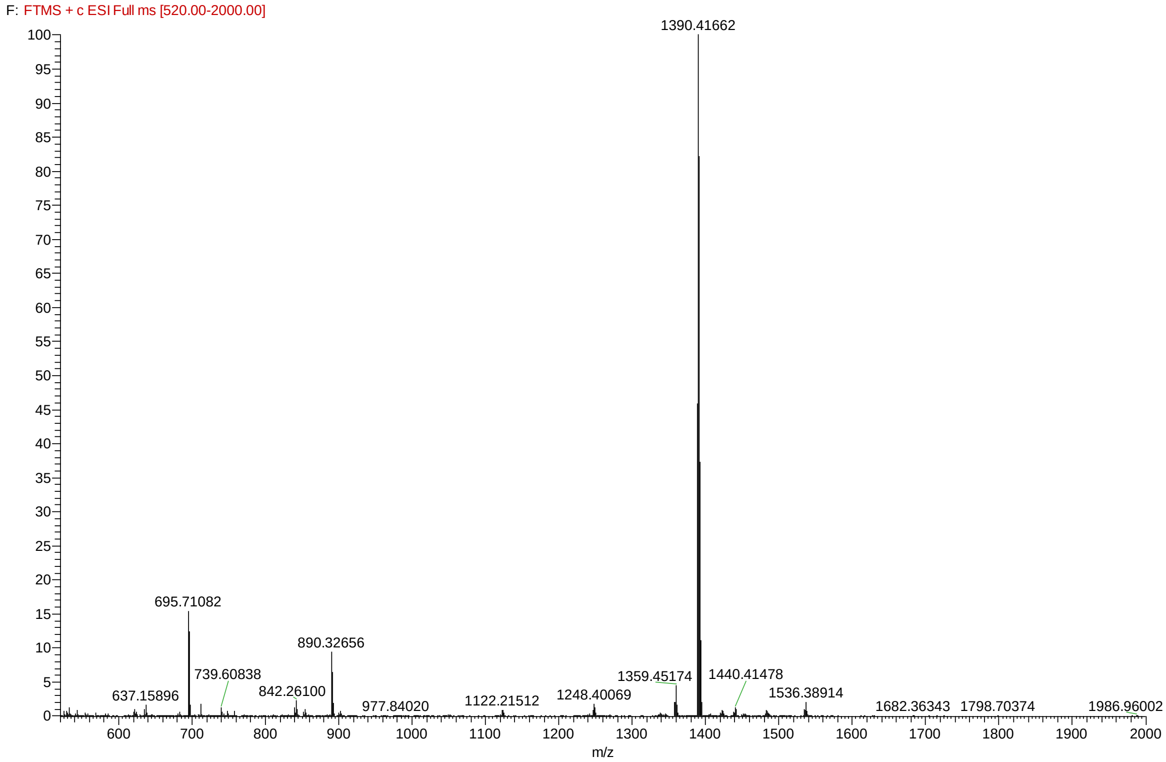

Figure S39. HRMS-ESI(+) spectrum of compound **4c**.

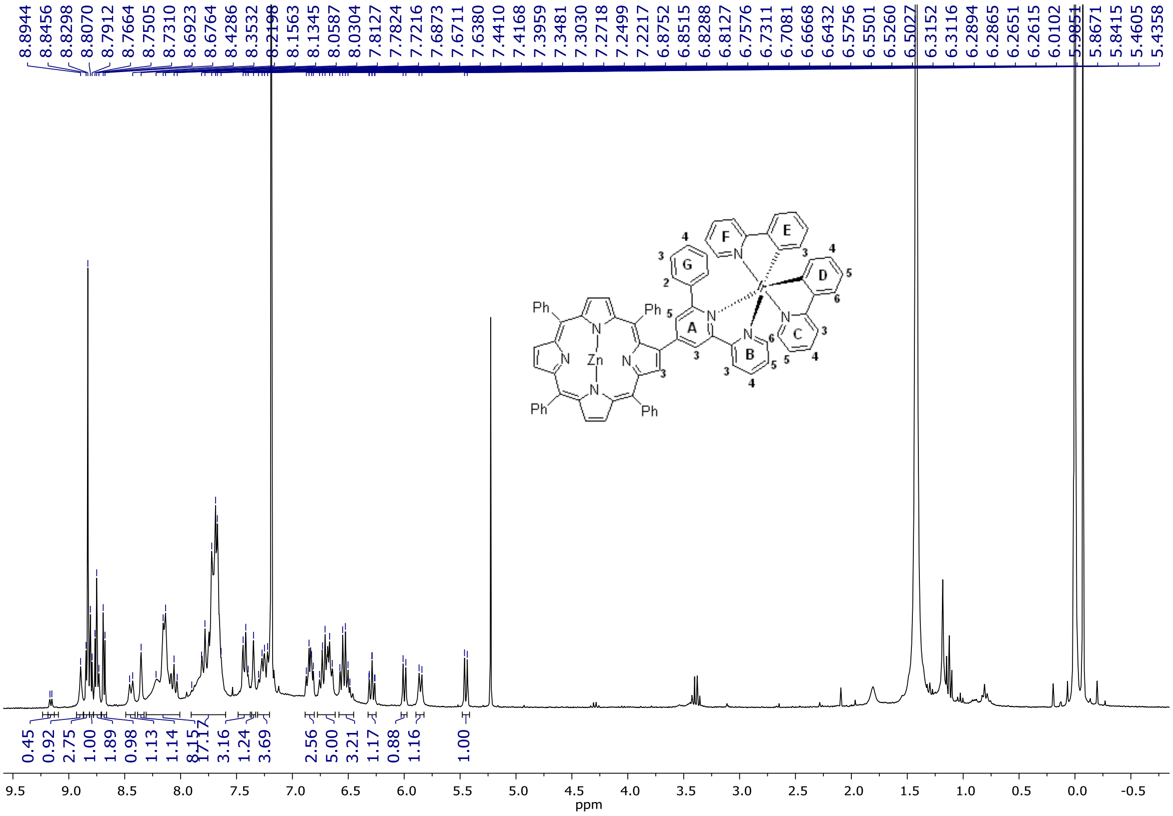

Figure S40. ^{1}H NMR spectrum of compound **5a** in $CDCl_3$.

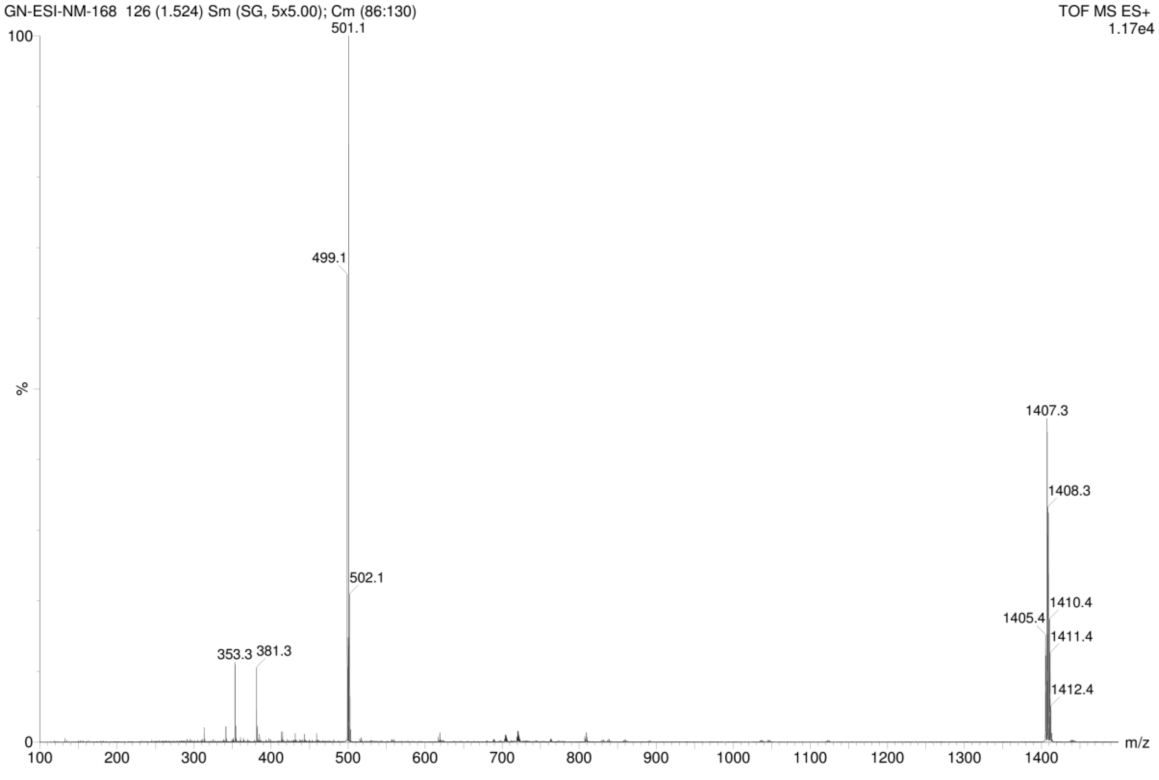

Figure S41. ESI(+)-MS spectrum of compound **5a**.

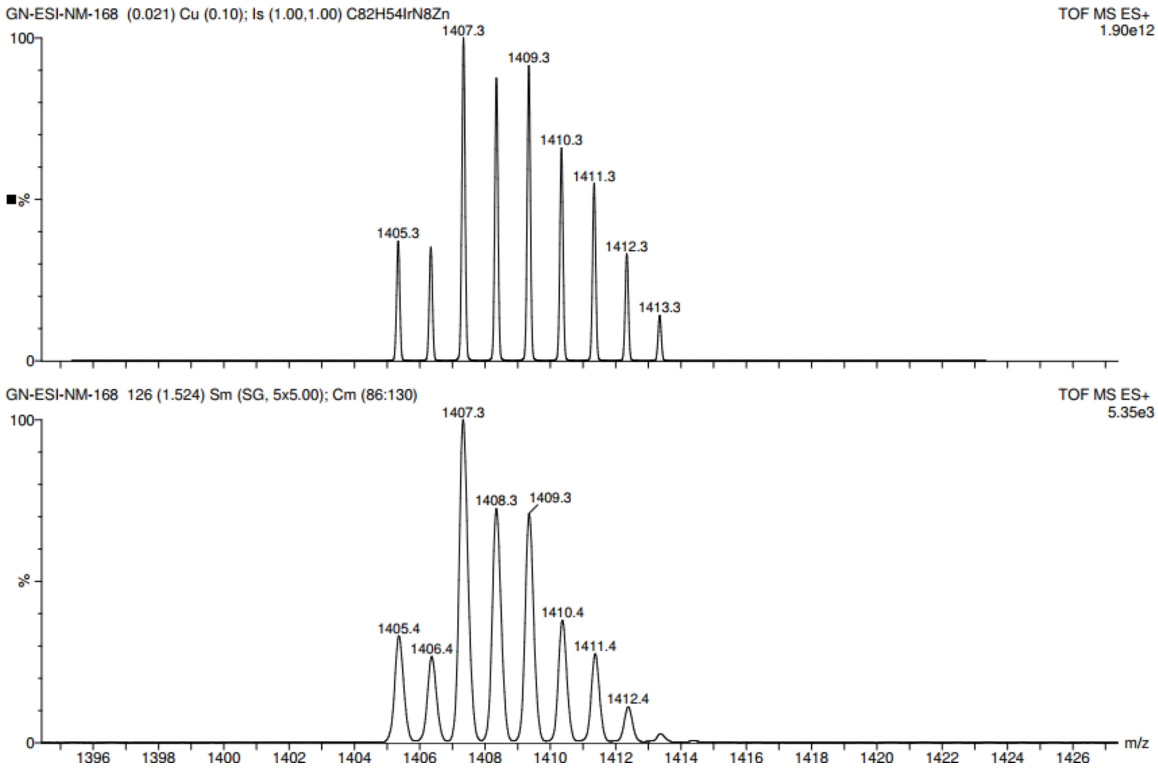

Figure S42. Isotopic pattern corresponding to the [M]$^{+•}$ ion of compound **5a**.

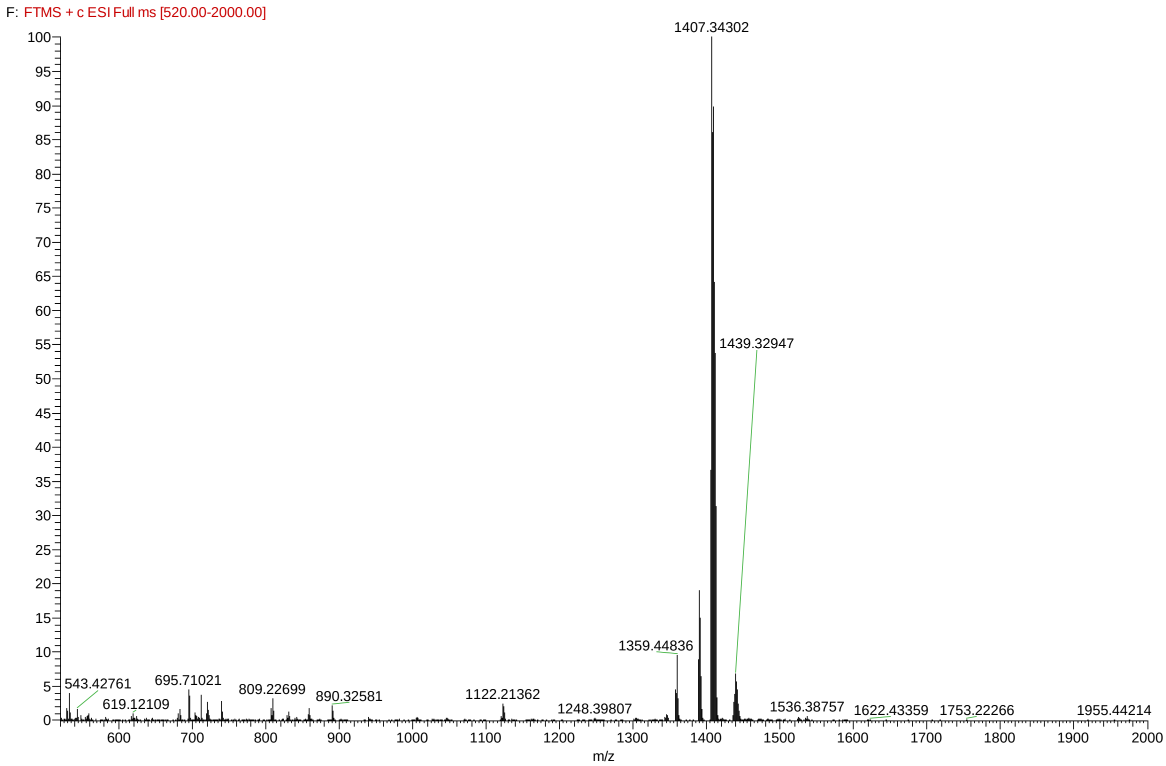

Figure S43. HRMS-ESI(+) spectrum of compound **5a**.

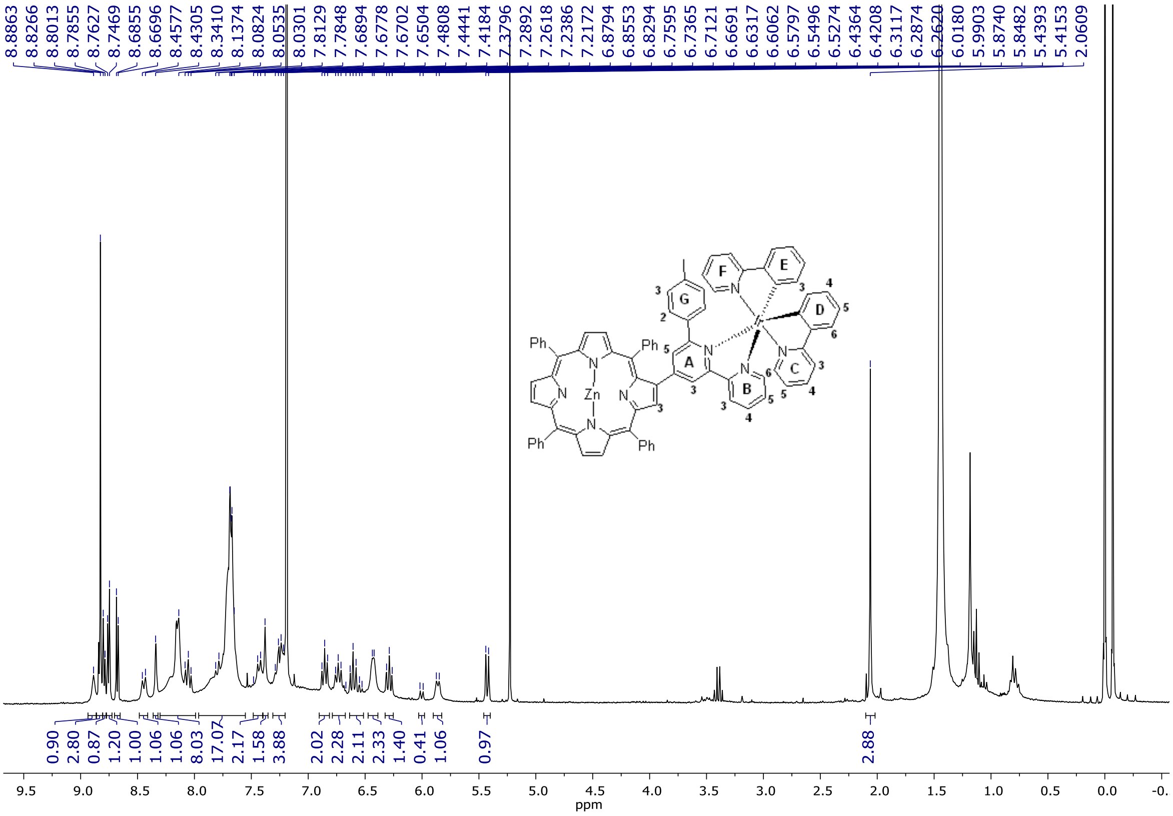

Figure S44. ^1H NMR spectrum of compound **5b** in $CDCl_3$.

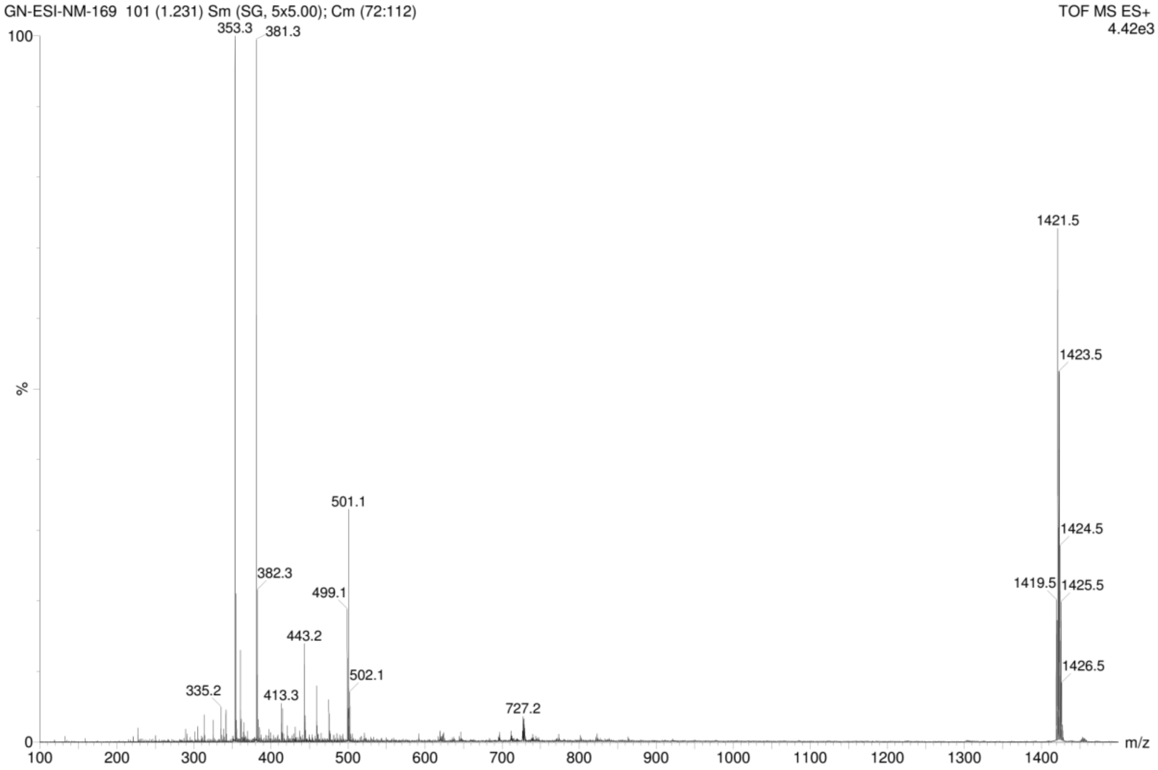

Figure S45. ESI(+)-MS spectrum of compound **5b**.

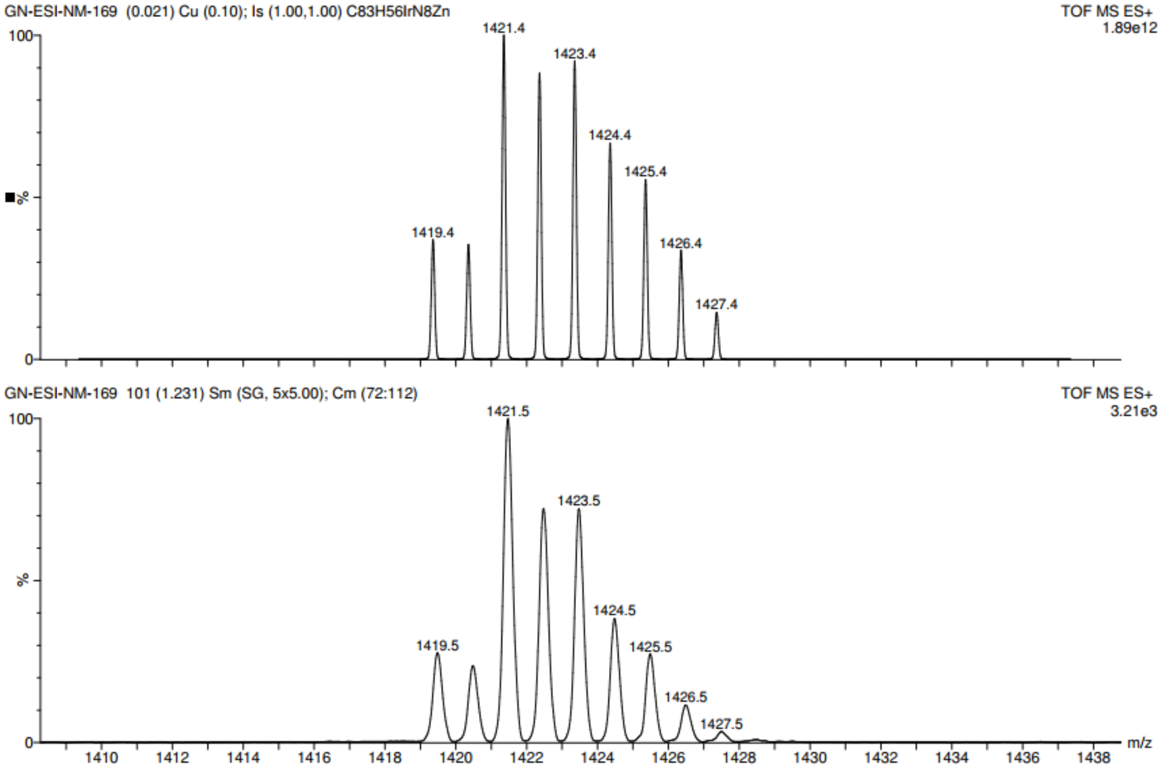

Figure S46. Isotopic pattern corresponding to the $[M]^{+\bullet}$ ion of compound **5b**.

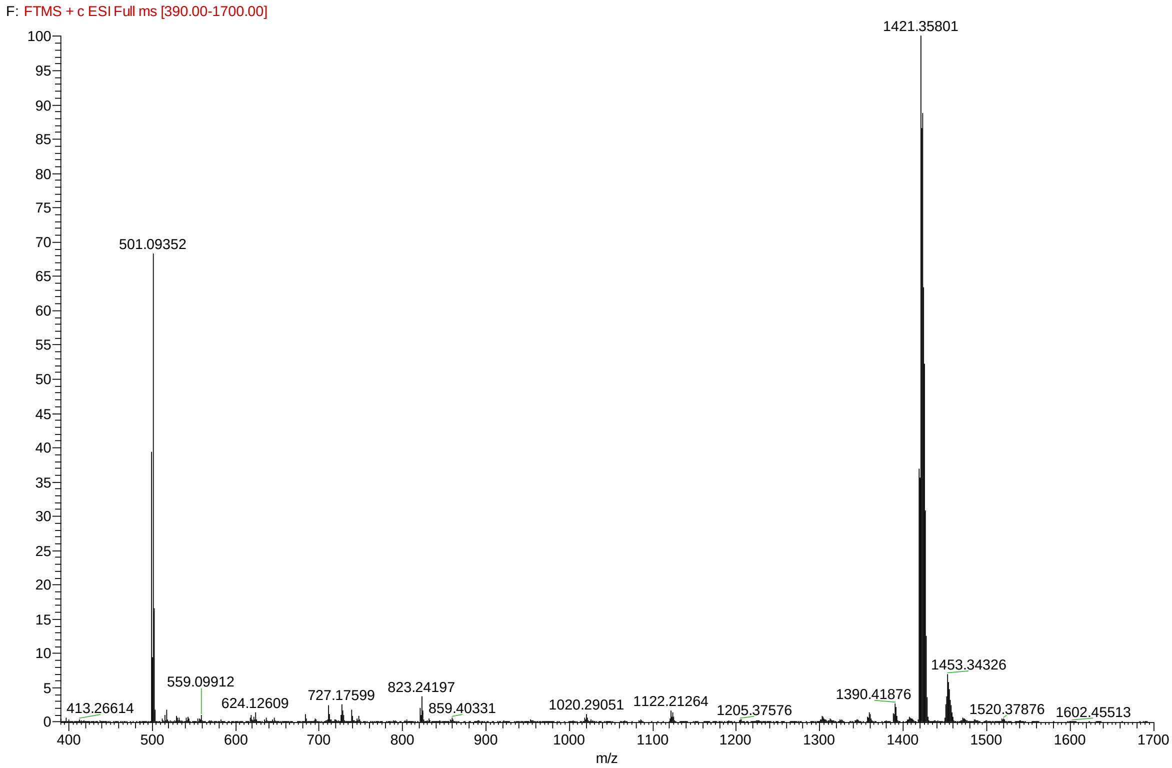

Figure S47. HRMS-ESI(+) spectrum of compound **5b**.

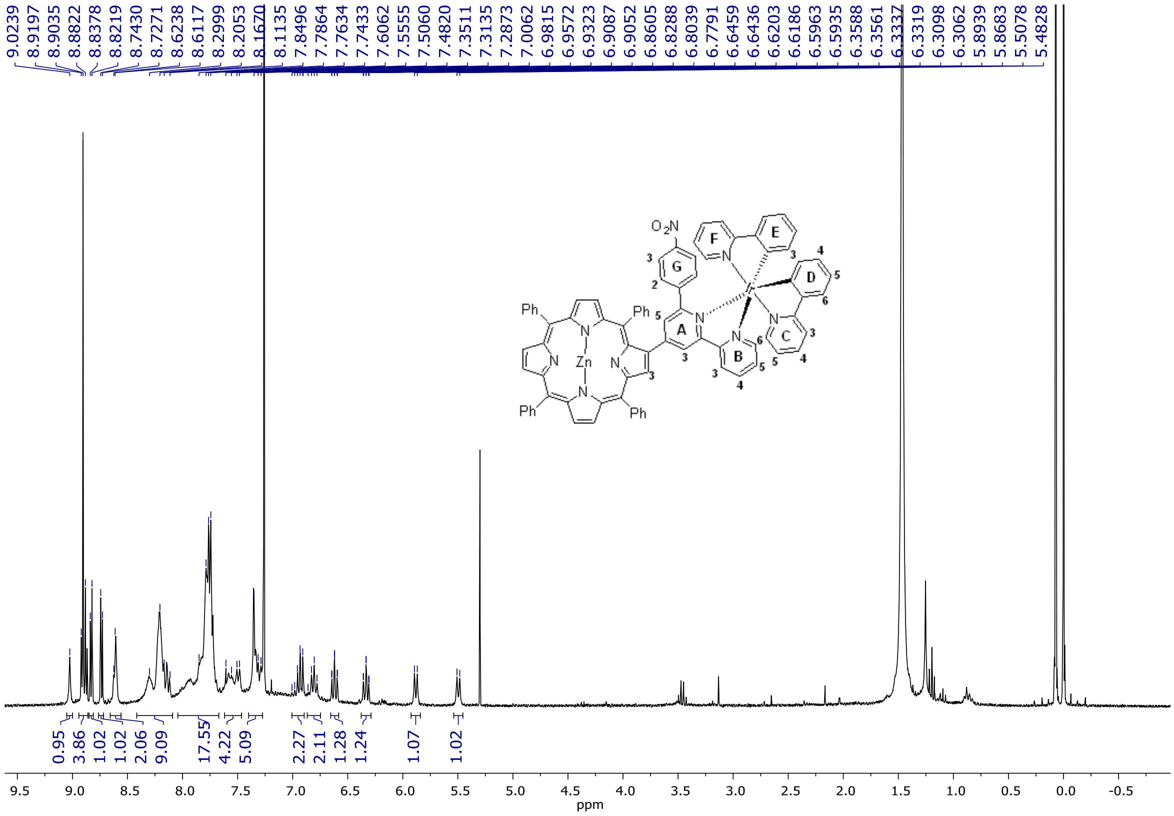

Figure S48. ^1H NMR spectrum of compound **5c** in $CDCl_3$.

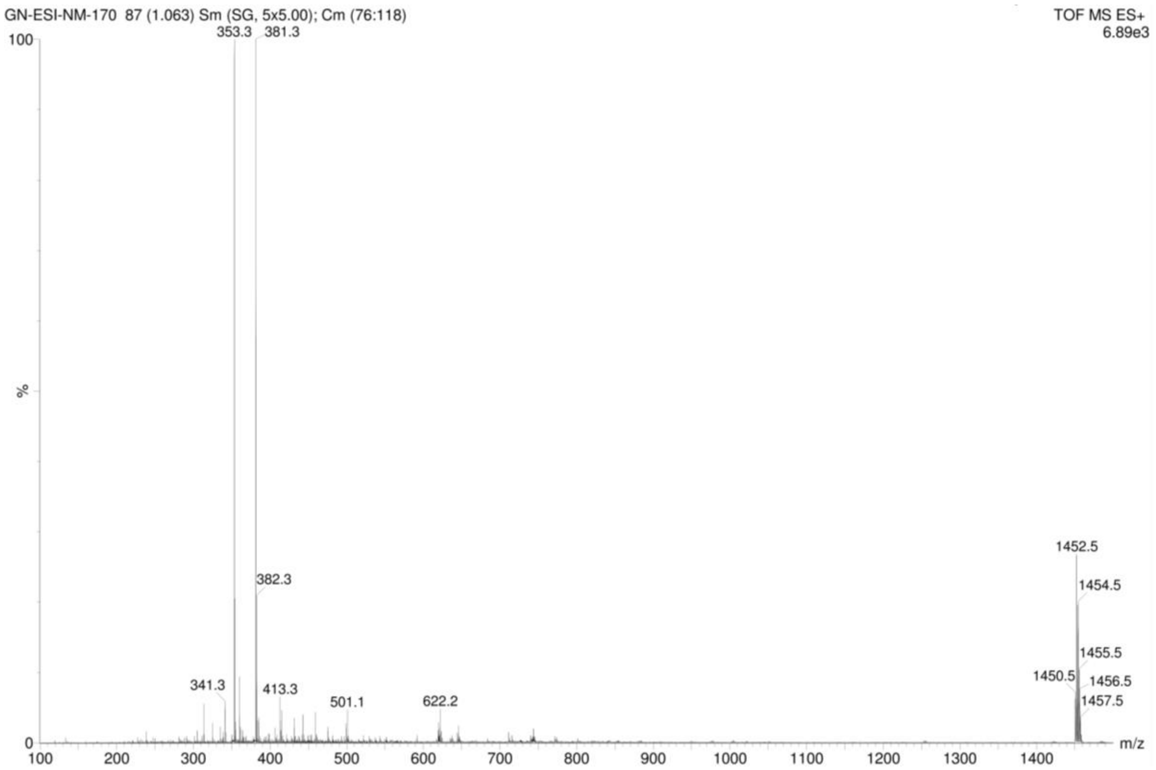

Figure S49. ESI(+)-MS spectrum of compound **5c**.

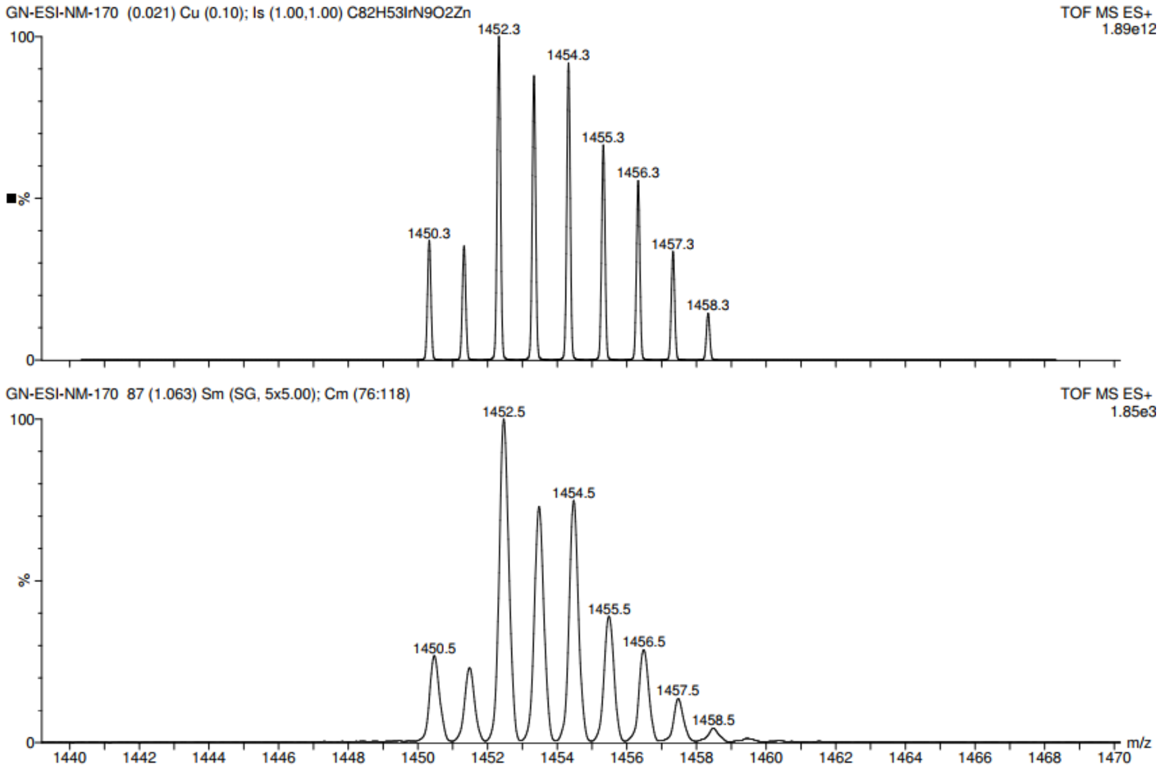

Figure S50. Isotopic pattern corresponding to the $[M]^{+\bullet}$ ion of compound **5c**.

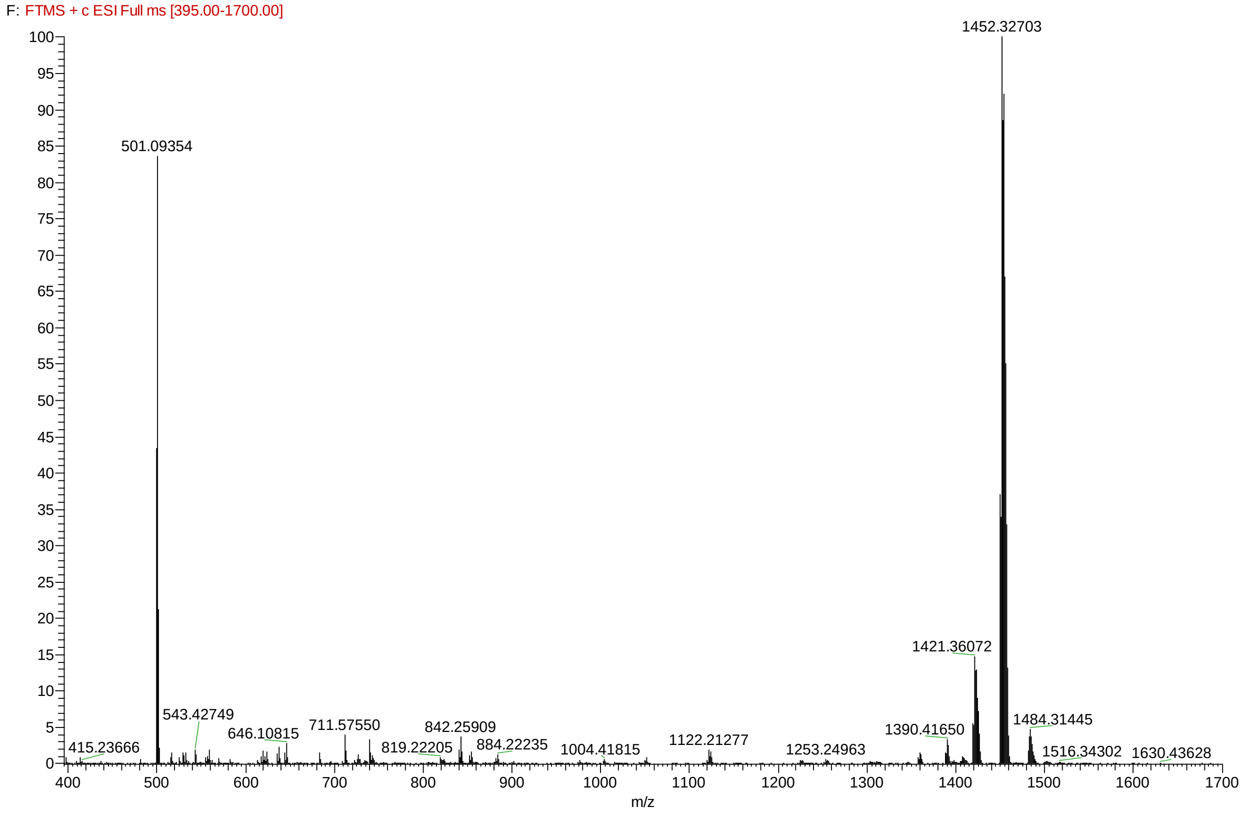

Figure S51. HRMS-ESI(+) spectrum of compound **5c**.

IV - *Cyclic voltammograms*

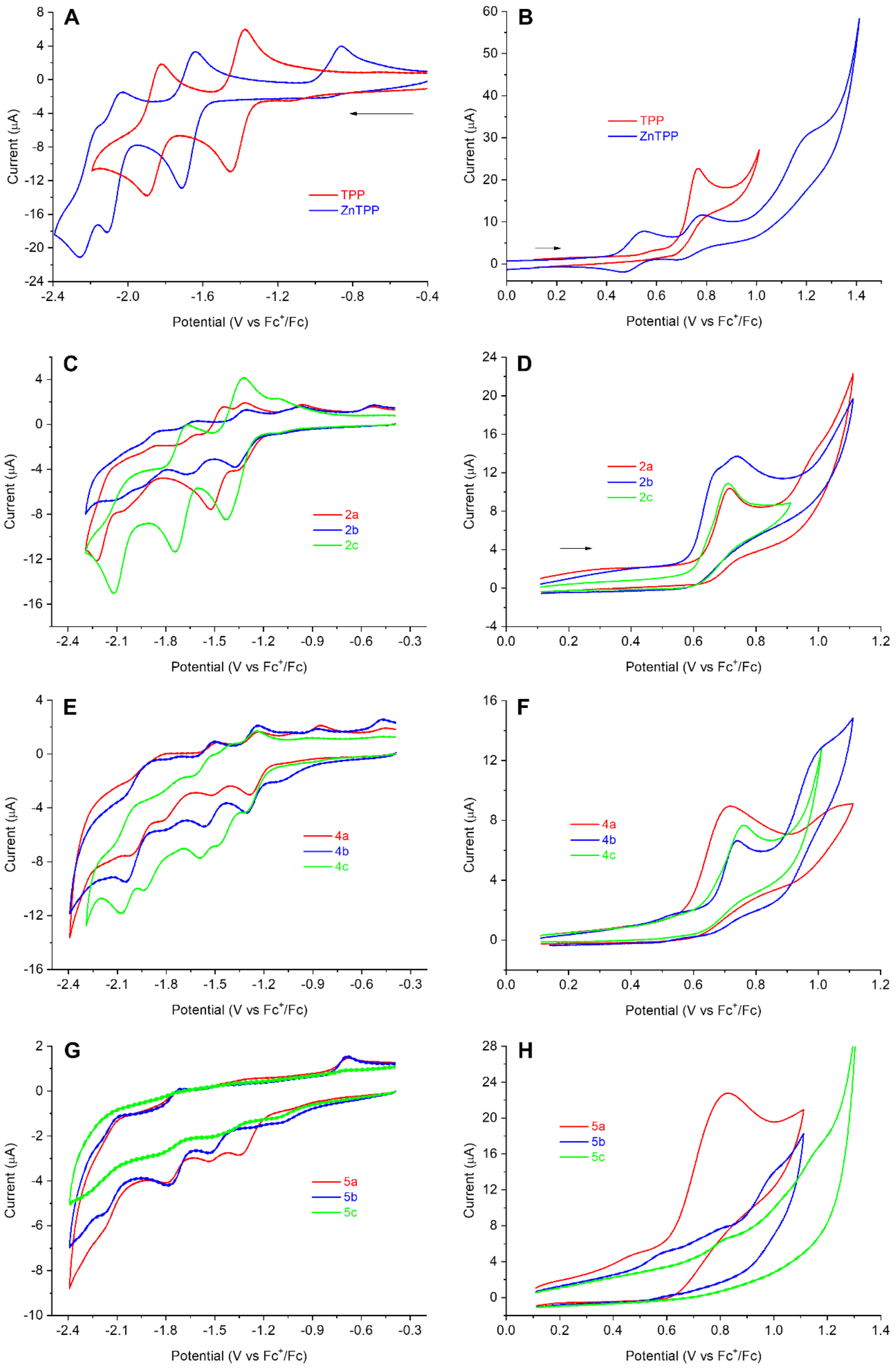

Figure S52. Cyclic voltammograms of **TPP** and **ZnTPP** (A,B), **2a-c** (C,D), **4a-c** (E,F) and **5a-c** (G,H); 1 mM in DMF with 0.1 M $TBAPF_6$ as supporting electrolyte obtained at a scan rate of 100 mV s^{-1}.

V - Absorption and emission spectra

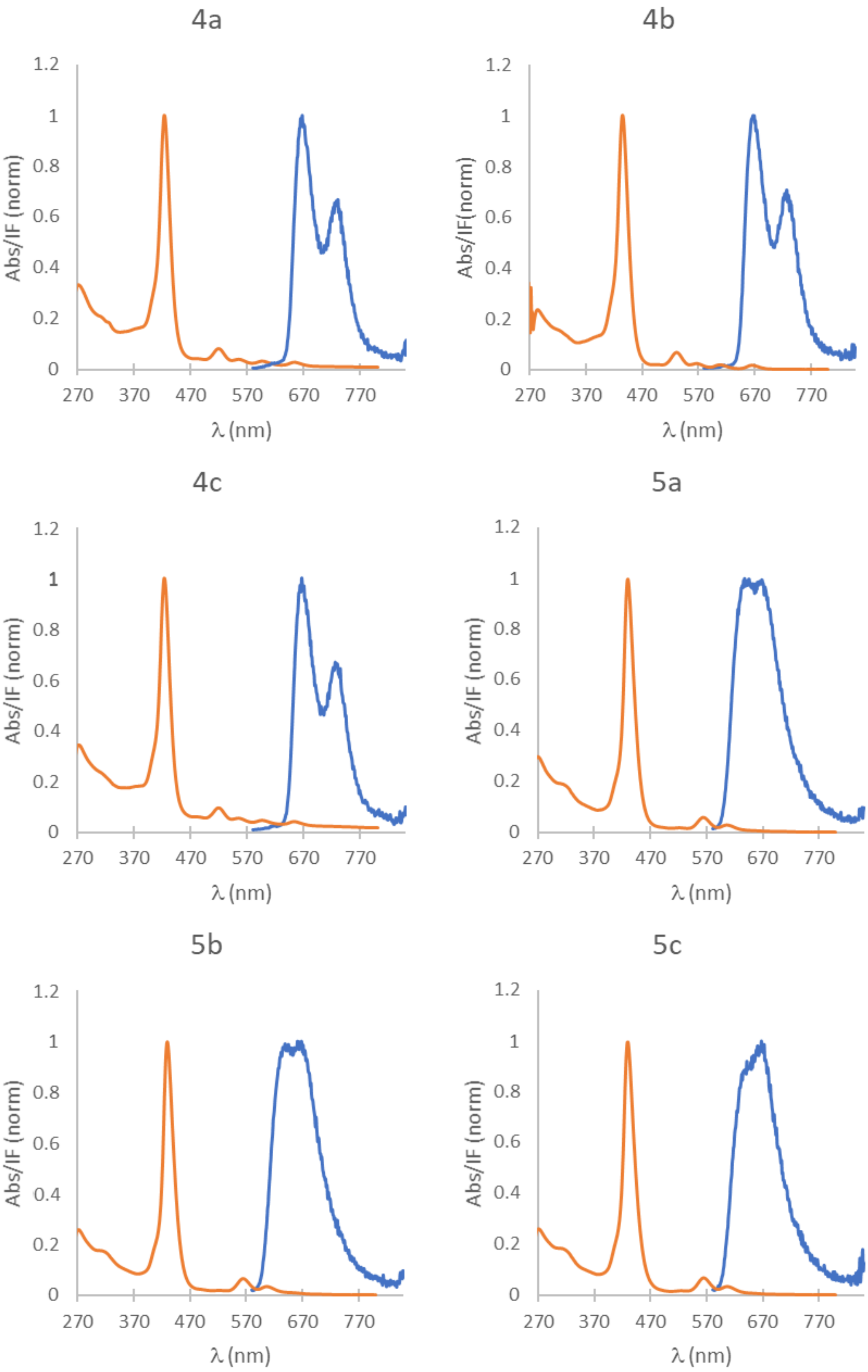

Figure S53. Normalized absorption and emission spectra of compounds **4a-c** and **5a-c** at 6x10^{-6} M in DMF at room temperature; λ_{exc}= 565 nm).

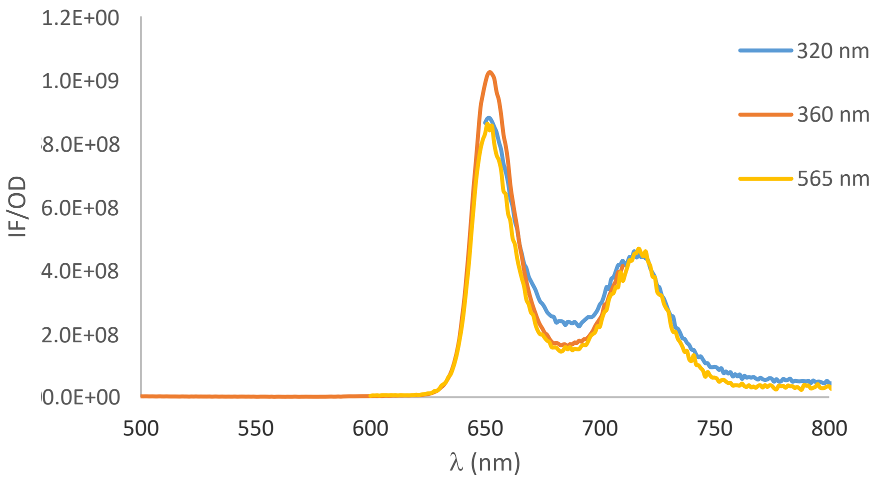

Figure S54. Emission spectra of **TPP** at different excitation wavelengths in DMF at room temperature.

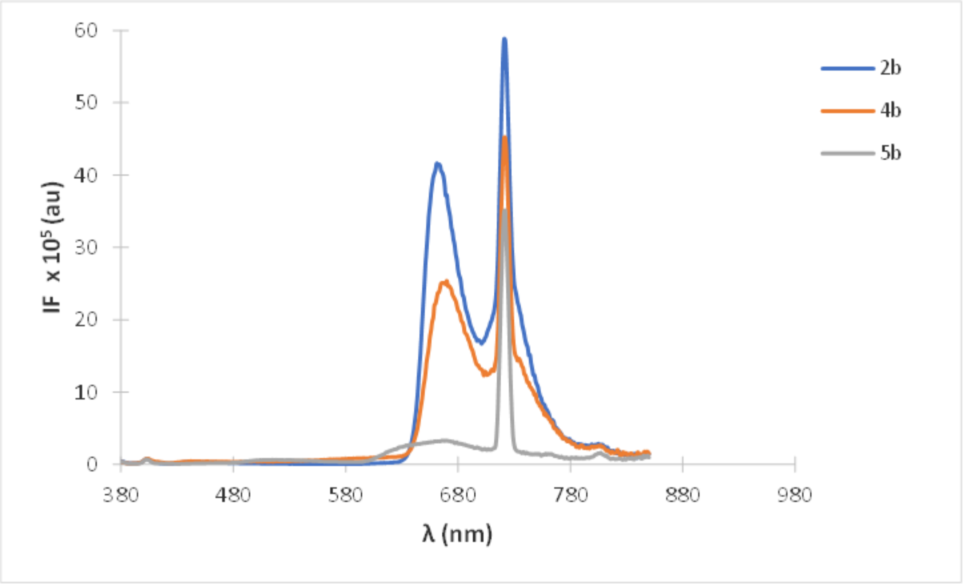

Figure S55. Raw data of fluorescence emission spectra of compounds **2b**, **4b** and **5b** in DMF at room temperature (non-degassed solutions) at a concentration of approximately 6 x 10^{-6} M and λ_{exc} = 360 nm, showing 2 x λ_{exc} at 720 nm.

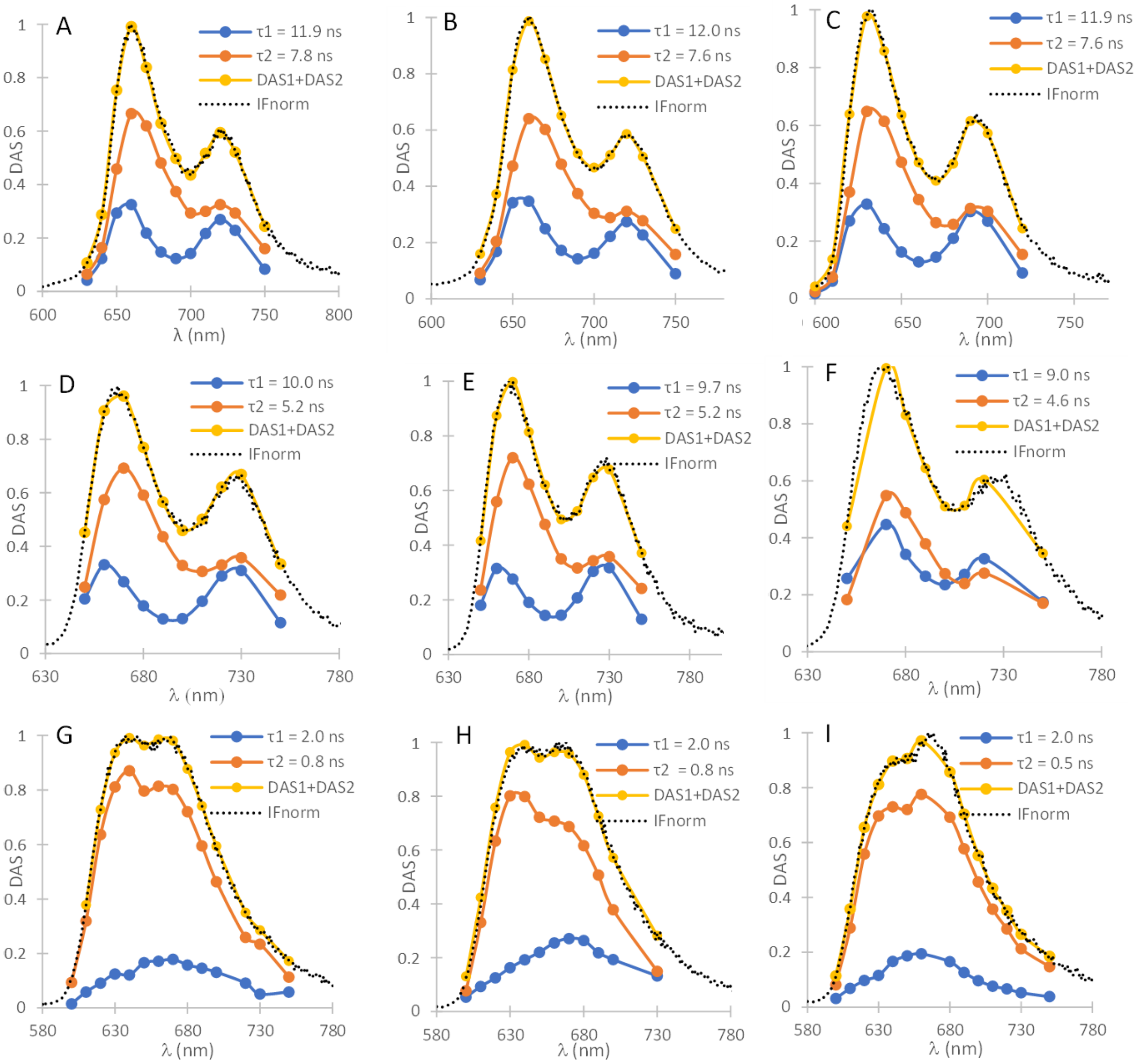

Figure S56. DAS spectra (in DMF) for compounds **2a** (A), **2b** (B), **2c** (C), **4a** (D), **4b** (E), **4c** (F), **5a** (G), **5b** (H) and **5c** (I). Excitation was done at 594 nm.

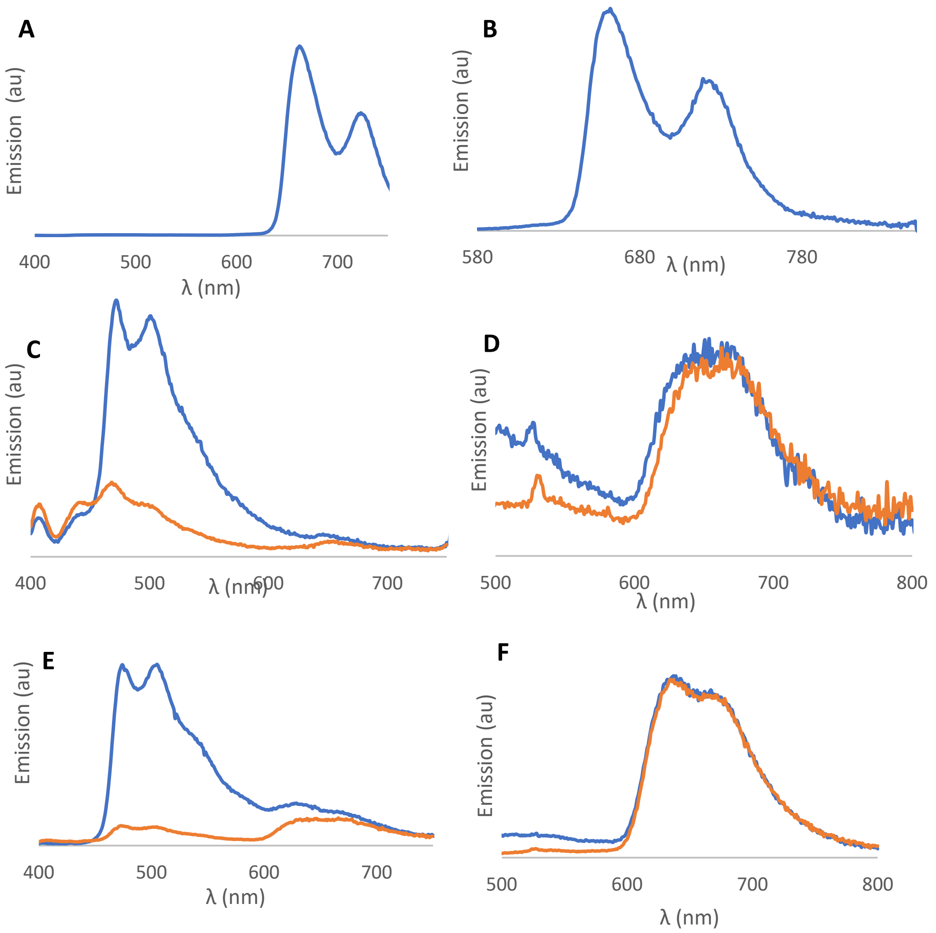

Figure S57. Representative emission spectra of compounds **2a** (**A**,**B**), **4a** (**C**,**D**) **and 5a** (**E**,**F**) 5 x 10^{-6} M in DMF degassed solutions (blue) and non-degassed solutions (orange) at room temperature; [(A, C and E) - λ_{exc} = 380 nm and (B, D and F) - λ_{exc} = 455 nm].

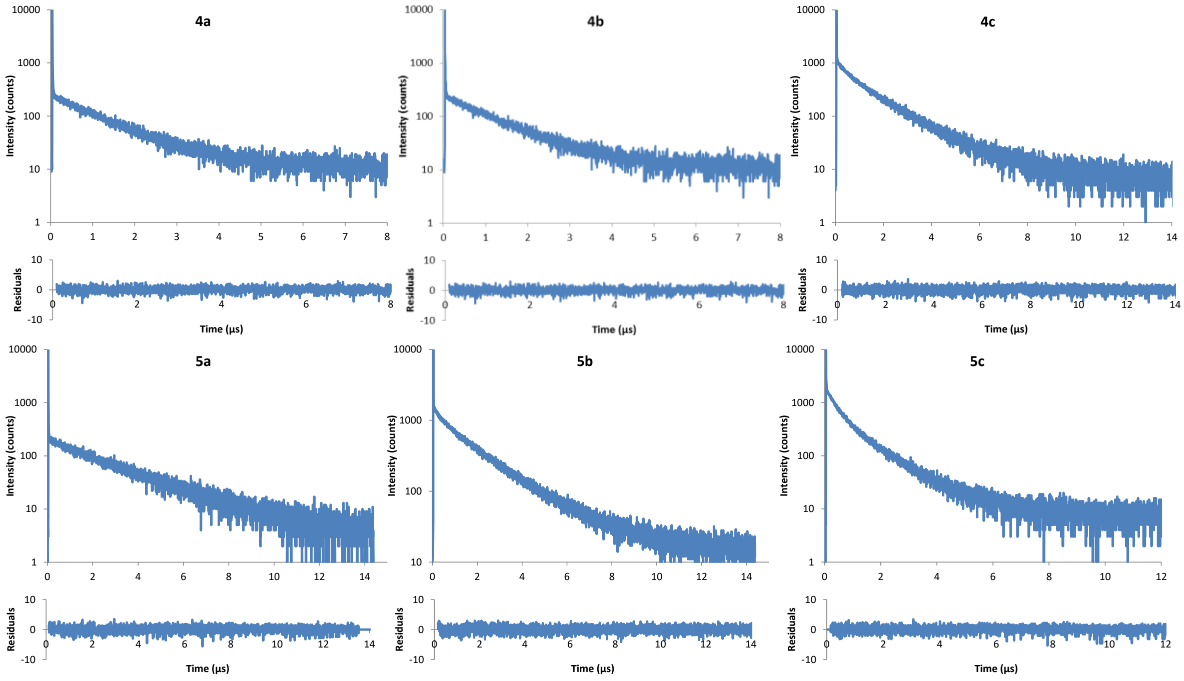

Figure S58. Emission lifetimes of Compounds **4a-c** and **5a-c** 5 x 10^{-6} M in DMF degassed solutions (λ_{exc} = 372 nm, λ_{em} = 508 nm)

Table S2. Emission lifetimes (τ_i) and respective pre-exponential factors (A_i) measured for porphyrins **4a-c** and **5a-c** in degassed DMF samples at room temperature.

Compound	τ_1(μs)	A_1 (%)	τ_2 (μs)	A_2 (%)	χ^2
**4a**	1.2	100	---	---	0.94
**4b**	1.2	100	---	---	1.00
**4c**	1.6	63.9	0.5	36.2	0.99
**5a**	2.6	100	---	---	0.06
**5b**	2.1	67.4	0.7	32.6	1.02
**5c**	1.3	63.5	0.4	36.5	1.02
